# Extended pharmacological thromboprophylaxis and clinically relevant venous thromboembolism after major abdominal and pelvic surgery: international, prospective, propensity score-weighted cohort study

**DOI:** 10.1093/bjs/znaf005

**Published:** 2025-03-11

**Authors:** EuroSurg Collaborative and STARSurg Collaborative, A Sgrò, R Blanco-Colino, N Brindl, D Chaudhry, K Gressmann, R R Gujjuri, A Hilder, E Matey, I S Pereira, A Turňa, C Varghese, W Xu, K A McLean, R Blanco-Colino, N Brindl, S Brown, W A Cambridge, D Chaudhry, K Gressmann, R R Gujjuri, A Hilder, A Jaffer, I Jakaityte, S K Kamarajah, M Kawka, O Kouli, E Matey, K A McLean, A Mergo, E C Mills, V Murray, Szy Ooi, I S Pereira, A M Riad, A Sgrò, S Q Shafi, I Trout, A Turňa, C Varghese, W Xu, B M Biccard, A Docherty, J Martin, K El-Boghdadly, M Phull, R Mouton, A Bhangu, J C Glasbey, E M Harrison, K A McLean, N Smart, S Moug, T Pinkney, T Richards, I Dajti, M de Cillia, G Van Ramshorst, S Delibegovic, H Mughal, J Mihanovic, N Gouvas, P Kocian, V Levesen, J Kaupilla, N Brindl, D Merz, M Joos, A Ioannidis, G Nnaji, F Pata, G Pellino, A Gori, M Podda, C Riboni, E Moggia, E Fekaj, S Oliver Senica, A Dauksa, J Psaila, F de Ruitjer, P Major, I Santos, M Sampaio Alves, J Simões, E A Bonci, A Pașca, A Novikova, B Kovačević, V Milosavljevic, B Tadic, J Kosir, R Blanco-Colino, S Pérez Ajates, M E Ossola Revilla, A Papadia, C Riboni, M L Gasparri, M K Aktas, B E Baki, M D Tepe, A U Mutlu, A Singal, J Osei-Bonsu, H Lacey, S W Chan, M Allison, K Duah-Asante, D Chen, N Ahmed, A Ejiz, C Takyi, M Mujeeb, N Ravikumar, M Khan, J Hayes, J Mckenna, J Wang, N Essa, H Xianghan, L Ko, Y Aldabbagh, J Plascevic, N Zia, R Ismail, Y Kamel, I Epanomeritakis, R Tan, N Chiu, A Naeem, M Kakwani, R Mehra, K Feeney, C L Yan Naing, A Qureshi, A Richens, H Li, R Ahmed, L Wilson, S Abraha, I Dajti, S Mikalauskas, J Kahn, D Kniepeiss, M Kušar, A Belarmino, J Frenk, I Mikalauskiene, A Angelis, J E Waha, S Al-Sharafy, M Sandano, P Schemmer, C Reiterer, K Horvath, A Taschner, S Riss, F Harpain, C Dawoud, N Hantáková, N Adamowitsch, S Schallmeiner, T Christian, V Xu, M M Kuhrn, K M Widmann, B Capek, B Kama, S Turgut, L Hauptmann, M de Cillia, M Grünbart, H Hoi, A Binder, T Gürtler, P Riedl, D Mayer, K Van Belle, O Wautelet, E Dekkers, E Van Daele, T Apers, G Van Ramshorst, F Berrevoet, N Rennie, P Dries, P Panta, A Denys, W Pype, T Bauwens, N Renard, S Violon, J Stijns, T Van De Winkel, E Van Eetvelde, M Salibasic, S Delibegovic, S Pusina, E Hodžić, M Kruščica, S Žilić, E Bicakcic, A Rovcanin, E Kulovic, E Halilovic, A Vinčević Hodžić, E Kalbić, S Delibegovic, A Mujić, M Mešan, E Matović, S Delibegovic, A Kesetovic, D Dardanov, E Arabadzhieva, B Markov, К Спасова, C Biji, N Oliveira, V Miroslavov, G Korukov, K Ravendran, N Shah, Y Mitkov, K Arivanandan, R Gaikwad, S Gotru, R Saraff, N Sain, D Stanchev, L Gaydarski, U Ali, L J Kandathil, A Krishnaveni, I Ivanov, I Ganev, Т Krapeshki, M Miza, Msk Mumu, I Bashliev, A Sajjad, L Chandel, D Yordanov, E Hristova, K Spassov, T Tsankov, T Ivanov, E Filipov, D Georgiev, V Neykov, E Daleva, I Ilieva, D Krasimirova, H Hristov, М Арсова, O Soladoye, A Biswakarma, R Raj, A Negi, A Dey, J K Adidela, U G Alionye, A Ndukwe, R Dimov, V Ivanov, S Sulcheva, S Mitrev, V Nikolov, M Karamanliev, D Dimitrov, A Yordanov, P Vladova, M Shoshkova, K Karakadieva, A Gabarski, A Shanker, H Ehtisham, S Parambi, L Tranchev, A Vasileva, J Kareem, J Ezeabasili, O L Madueke-Ediae, T Muradia, A Saini, I Rana, R Kanagaratnam, L S Thomas, M Galasyuk, J J Costa, N Majid, P Reddy, M Abdullahi, S Koshy Thomas, A Mehta, A Biswakarma, R Raj, S Shittu, J Theophilus, J Theophilus, R Ilyas-Uddin, I Ogunleye, E Naghavi, A Kiran, A Sundar, A Joy, F Ali, I Angelova, M Slavchev, N Belev, B Atanasov, P Krastev, T Yotsov, D Dimitrov, P Kamenova, I Mihaylov, A Stavrov, A Vricheva, G Šantak, L Penezić, Z Kastelan, Z Zimak, H Saić, N Knezevic, B Cikic, T Zekulić, T Hudolin, I Juric, J Anđelić, T Kulis, M Maric, J Mihanovic, T Soric, D Surić Grčić, L Blagus, P Kovačević, I Vidić, A Miočić Juran, C Ergatidou, S Gravas, A Yiallourou, S Achilleos, M Vardas, G Kokkinos, C Panagiotou, M Kyprianou, K Kyriacou, P Papatheodorou, K Konstantinou, D Onisiforou, F Tsoutsouki, A Kasapi, E Demetriou, A Stylianou, E Xenophontos, P Georgiou, P Makrides, I Pozotou, M Constantinou, T Evangelou, A Varavina, S Charitonos, M Lampi, M Attaalla, T Dušek, M Ndukwe, L Kuzmane, E Moorlata, H Sillah, J Kotek, J Fazal, E Ssali, V Steingauer, K Vinklerova, O Ahmad, Á Rodríguez Martínez, E Hiebert, M Srivastava, A S Ramesh Babu, O Dobra, A Akiba, M F Kalou, T Sokolová, N Nepeřená, P Novotný, J Sedlackova, F Philips, H Al Atassi, P Jose, A Sunil Nair, L Ntashamaje, A Kaddah, M A Antabi, P Kocián, R Fiala, T Harustiak, S Vesely, A Haluza, A Štekrtová, V Král, K Zdichová, J Pastor, A Gorchakov, H Novák, V Novák, J Hornak, M Havova, M Marková, T Fořtová, D Lérias Bento, R Novysedlák, J F Smetana, T Novák, T Zdobinska, M Švarc, E Hejduková, K Havlová, A Vaishevich, J Čorňáková, P Zajíc, P Francúz, O Příman, Š Havlíková, B Zemlickova, A Whitley, R Gurlich, P Balaz, I Tomyak, M Kútik, O Molva, D Šturc, M Belbl, T Vinklarkova, V Villefranque, A Police, E Mikhael, A Mabilia, L Charre, E Volpin, H Braham, R Montero Macías, D Krief, A Boyer De Latour, M A Angeles, A Martinez, A Navarro, G Ferron, M Del, M Ghiani, F Migliorelli, M Danguy Des Déserts, S Johan, B Olivier, C Andro, D Von Wedel, C Kamphues, M Thiele, J Felber, J Neudecker, C Schineis, J C Lauscher, R Stephan, L P Meyer, C Wolff, B Bombera, J Rolinger, A Kirschniak, P Wilhelm, S Göller, L Van Den Hil, D Reim, M Kießler, C Buschhaus, S Naisar, N Zahlmann, R L Walter, M Weber, K Müller, A Schweden, M Berlet, N A Jorek, S Seyfried, N Rahbari, E Birgin, C Reissfelder, A Reeg, P Téoule, E Rasbach, N Pyrgidis, G Hatzichristodoulou, I Sokolakis, J J Strotmann, T Fahlbusch, P Höhn, J Hinrichs, J Horn, S Jollet, J Binder, T Förtsch, M Winterstein, D Hackner, A Girard, M Wittmann, T O Vilz, F Recker, M Velten, G Massoth, A Delis, M I Brugués Villalba, M Willis, S Soltau, S Kudaliyanage, R L Eymael, I Syring, R Neubauer, N Dahmen, A Zimmermann, N Straßberger-Nerschbach, A Puskarevic, X Wang, L Peyman, R A Philippi, K Schmidt, U Bork, F Schepp, S Korn, O Radulova-Mauersberger, J Von Dem Bussche, F Von Bechtolsheim, C Praetorius, N Knobloch, F Oehme, J Weitz, M Mansfeld, M Distler, F Klingler, G Fluegen, S Dávid, M Schneider, K A Baba, U Ronellenfitsch, J Kleeff, J Ukkat, J Klose, O Bayram, M Sommerer, A Rebelo, I Gockel, E Gentsch, N Rayes, R Thieme, N Kreuser, B Aktas, D Branzan, D Seehofer, M R Mallmann, C Mallmann, S Woldu, F Balci, C Domröse, M Diekhöner, G Micha, K Stroumpoulis, L Dritsoulas, K Kalopita, G Bitzi, P Thessalonikefs, E Fradelos, D Korkolis, A Sarafi, D K Manatakis, N Tasis, A Prountzopoulou, E Tziava, K Pikoulas, D Paramythiotis, M Tishukov, K Papadopoulos, G Chatziantoniou, G Tzikos, S Papamichail, S Bareka, A Sarafis, D Haidopoulos, K Angelou, A Prodromidou, E Stamatakis, N Alexakis, V Pergialiotis, A Rodolakis, N Thomakos, N Memos, N Vlahos, I Koutalas, T Kotsis, E Kalampokas, L Chardalias, C Kontopoulou, C Lignou, P Lykourgioti, A Bagiasta, A Petrolekas, O Savranakis, T Kozonis, V Themelidi, M Mccormac-Prekeze, C Nikolaou, E Bletsa, N Melissaridou, K Flamourakis, P Rammos, T Hadjizacharias, N Provata, D Politis, A P Gkioulekas, D Massaras, I Vakos, A Antonoglou, P Dimitra, A Kotzadimitriou, C Evangelou, D Psychogios, M K Konstantinidis, K Apostolou, K Konstantinidis, N Patelis, S Konstantinidou, P Kokoropoulos, N Michalopoulos, Z Kratiras, P Drakakis, T Sidiropoulos, M Papadoliopoulou, P Vassiliu, N Arkadopoulos, C Koratzanis, N Vrachnis, E Dylja, I Chatzialis, D Sampanis, N Danias, A Fotiou, S Stavros, Z Petropoulou, V Tsaousis, D Papakonstantinou, P Lykoudis, C Nastos, A Charalampopoulos, I E Papiri, I Hatzaras, K I Paraskevas, G Petrakis, E Polenta, E Kaparounaki, M Sotiropoulou, S Kapiris, E Mavrodimitraki, A Paraskeva, A Kolinioti, M Psarologos, D Stergiou, P Metaxas, K Stamatis, C Kyzeridis, E Kefalou, G Z Vrakopoulou, A Larentzakis, E Menenakos, V R Maravgaki, C Georgiadou, M Koutrouli, A Papageorgiou, A Skandali, G Tzovaras, I Baloyiannis, V Tzortzis, G Christodoulidis, K Perivoliotis, E Arnaoutoglou, M P Ntalouka, A Chatzis, A Daponte, A Samara, C Donoudis, D Zacharoulis, F Mulita, K Bouchagier, G Verras, L Tchabashvili, O Ioannidis, A Koltsida, A Malliora, D Paparouni, S Bitsianis, L Loutzidou, E Anestiadou, Χ Χατζηανεστιάδου, S Simeonidis, A Tekelidis, K Zapsalis, S Skalidou, N Ouzounidis, G Ntampakis, A Kelepouri, F Kontidis, V Foutsitzis, O Kontaxi, G Barakakis, O M Valaroutsou, C Athanasiou, E Kotidis, M Pramateftakis, I Mantzoros, K Toutouzas, M Frountzas, T Triantafyllou, A Triantafyllou, T Dagklis, G Katsanos, S Petousis, A Athanasiadis, G Tsoulfas, K Dinas, I Tsakiridis, A Mamopoulos, I Kalogiannidis, F Rao, C Christou, S Vasileiadou, G Kapetanios, N Tsakiridis, S Kopatsaris, E Papanikolaou, K E Karakasi, T A Tataridou, N Antoniadis, F Zachomitros, A Arvanitaki, K Tsakiridis, M Anemoulis, S Neiros, K Ouranos, E C Tampaki, C Maltezos, K Maltezos, C Anastasiadou, A Chaveles, A Pachi, P Tsiantoula, K Roditis, A Antoniou, N Bessias, T Papas, S Tzamtzidou, D Maras, V Papaioannou, G Koukoulis, K Bouliaris, K Skriapas, G Kontopoulos, C Doudakmanis, C Kolla, M Efthimiou, C Kalfountzos, D Mitsakou, C Kardasi, S Zourntou, L I Fountarlis, A Bakalis, M Samarinas, P L Chatzilamprou, A Migdanis, K Karvouni, K Katsiafliaka, C Arvaniti, A R Papazisi, V Markatou, K Zervas, K Marsitopoulos, N Machairas, P Dorovinis, E Kotsifa, M D Keramida, D Schizas, M Vailas, A Syllaios, E Mela, N Hasemaki, A Skotsimara, A Katsargyris, S Kykalos, N Tomara, I Palios, I Karniadakis, A Charalabopoulos, P Sakarellos, S Davakis, N Kydonakis, G Tsourouflis, P Stamopoulos, K Laios, A Kozadinos, I Katsaros, E Kontis, C Iavazzo, L Katsiaras, E Kaouras, P Manikis, K Kokkali, G Theodorou, A Dragi, G Vorgias, L Tzelves, A Skolarikos, I Manolitsis, M Spartalis, I Tzima, A Anastasiou, P Pantelidis, G Schismenou, E Spartalis, G E Zakynthinos, K Lasithiotakis, G Petra, M Lampou, D Toth, Z Varga, J Pósán, L Illésy, C Váradi, M Santarelli, L Puca, L Brignone, R Lisa Marie, L Silvia, P Silvia, A Ferguglia, C Piceni, M Improta, F Assanti, E Montanari, G Ettore, F Cannone, L Cormaci, V Nicastro, R De Carlis, G Ferrari, A Giani, S Grimaldi, L Gregorio, M Mazzola, L Ripamonti, L Lorusso, G A Tartufari, C Magistro, A Benedetti, A Gonta, J Maesano, C L Bertoglio, I Giusti, O Quagli, F Brucchi, E Bevilacqua, L De Carlis, A Lauterio, R Cerchione, M Migliorini, N Incarbone, L Centonze, S S Darwish, I Vella, V Buscemi, A Podestà, M Desio, M Berselli, S Megna, E Cocozza, L Livraghi, V Quintodei, V Marchionini, G Borroni, C Peverelli, M Corbella, F Ferrari, F Odicino, E Gozzini, F Cisotto, G Baronio, R Del Giudice, G Sala, A Iacomino, G Vennarecci, D Ferraro, M Di Martino, D Pisaniello, F Falaschi, L Petagna, A Giuliani, P Di Lascio, M Coluzzi, G Pascale, P Gallicchio, G Frezza, G Dinatale, G Cerino, A Bottari, J Martellucci, G Maltinti, M Scheiterle, L Fortuna, F Staderini, F Coratti, M Veroux, A Giaquinta, G Roscitano, A Volpicelli, P Veroux, M Palumbo, G Riccioli, L Stella, C Distefano, R Gioco, G Lomeo, E Lomeo, M Cavallo, C Virgilio, C Molino, M M Giambra, D Zerbo, R Santini, S Costa, G G Incognito, D C Centonze, G Currò, M Ammendola, R Filippo, G Ammerata, R Balestri, L Morelli, D Tartaglia, M Puccini, V F Asta, V Conte, P Buccianti, B Sargenti, G Boni, S Signori, D Pezzati, L Urbani, G Casale, G Taddei, G Di Franco, M Bianchini, C Carpenito, N Furbetta, A Comandatore, F Tarasco, S Guadagni, M Mastrangelo, C Gianfaldoni, M Palmeri, L M Fatucchi, A B Boato, D Gianardi, M Stingone, L Sacco, S Giaquinto, L Lami, M Iuliano, E A Annunziata, M Chiarugi, S Strambi, F Coccolini, G Anania, M Chiozza, A Campagnaro, V Nevoso, L Carbone, L Marano, G Micheletti, A Fontani, S Malerba, M Gambelli, A Bombino, G Grassi, V D Mandato, L Aguzzoli, V Mastrofilippo, N Fabbri, C V Feo, M Ginestri, D Oppici, M Torchiaro, A Pesce, F Catena, C Vallicelli, V Murzi, M Podda, A Pisanu, F Campus, T Pilia, A Saba, C Piras, M Pisano, E Locci, P Marongiu, E Gessa, Mario D’Oria, A Biloslavo, S Lepidi, P Germani, C J Nappi, B Grando, A Lauretta, S Pollesel, P P Brollo, G Gallo, M Trompetto, A Realis Luc, V Tiesi, A Porcu, T Perra, M Madonia, A Tedde, F Scognamillo, P L Tilocca, D Delogu, G Mucci, G Drocchi, M Tedde, A M Scanu, G Rizzo, A Fancellu, C F Feo, G Farina, R Casu, M L Cossu, G C Ginesu, M Anania, S Dessole, G Capobianco, M Petrillo, F Dessole, I Angelone, A Barberis, A Azzinnaro, A Petrungaro, E Palli, E Mina, N S Pipitone Federico, A Muratore, M Calabrò, M De Zuanni, E Herranz Van Nood, L Licari, C Callari, D Di Miceli, S Viola, M Manigrasso, C Caricato, M Milone, P Anoldo, S Vertaldi, A D’amore, G D De Palma, A Marello, C G Cantore, A Chini, F Maione, G Luglio, G Pagano, F P Tropeano, M Cricrì, G Aprea, G Palomba, M Capuano, R Basile, U Bracale, R Peltrini, M Visconti, G Magno, A G Di Santo Albini, P Fransvea, G Sganga, G Tropeano, C Puccioni, G Altieri, V Fico, P Mirco, V Bianchi, V Cozza, G Pepe, S Alfieri, F Rosa, C A Schena, V Laterza, M M Pascale, S Agnes, G Bianco, F Frongillo, F Ferracci, F Santullo, A Balla, P Lepiane, F Saraceno, R Mastroianni, G Simone, G Tuderti, F Marino, G Lantone, G Lantone, R Isernia, F Pezzolla, F Aquilino, D Raimondo, A Gori, R Seracchioli, M Rottoli, A Belvedere, L Maurino, I S Russo, B Orsini, M Maletta, A Romano, P Bernante, T Violante, D Morezzi, E Degli Esposti, A Raffone, G Poggioli, A Canavese, G Dajti, S Cardelli, P Casadio, A Arena, G Sanna, D Cuicchi, L F Angelicchio, C Catalioto, B Torre, P De Iaco, M Tesei, E De Crescenzo, C Larotonda, F Greco, M Minghetti, L Sissa, A M Perrone, G Dondi, M Di Stanislao, L Gaetani, L Serafini, E Prosperi, D Perone, C Isopi, F Bruno, I D Alexa, P Milito, A Lovece, G Chiappini, C Froiio, T Panici Tonucci, G Saletta, A Scardino, A Belli, F Izzo, R Patrone, C Cutolo, R Palaia, V Granata, V Albino, M Leongito, M Piccirillo, G Pasta, P Delrio, D Rega, M Rho, A Aversano, I A Angelone, M D’amico, M Di Marzo, S De Franciscis, P Cianci, E Restini, I Conversano, G Scialandrone, S Di Saverio, L Cardinali, G Travaglini, E Sebastiani, I Marziali, A B Bellocchia, I Raimondo, D Di Giorgio, G Garganese, V Tondolo, A Dore, G Pacini, L C Turco, P Campennì, M A Amara, A Rubattu, D Verri, G Sole, S Bove, F Inzaina, F Franceschi, G Grande, D P Pili, F Campus, G Delogu, V Sula, O Ez Zinabi, M Congiu, E Pira, G Ercolani, F D’acapito, D Annunziata, L Solaini, A Giordano, S Cantafio, S Novello, M Romano, C Armellin, G Zanus, F Milardi, U Grossi, R Baldan, M Brizzolari, F Scolari, A Brun-Peressut, A Broglia, M Martorana, N Fazzini, C Marafante, M Garino, E Moggia, S C Agosti, A Battaglia, A Borello, S L Birolo, R Barone, C Mosca, K Shakhova, A L Apostu, S Giaccari, M Pavanello, E Migliori, D Sambucci, C Corbellini, G M Sampietro, G Germiniasi, M Bischeri, F Colombo, P Danelli, F Cammarata, R S Zingale, I Pezzoli, M Carbonaro, F Albanesi, F Cozzani, M Rossini, F De Gennaro, M Giuffrida, P Del Rio, M Inama, G Moretto, L Iudici, A Vitali, M Creciun, H Impellizzeri, M Piazza, A Biancafarina, M De Prizio, R Malatesti, R Sulce, G A Pellicano’, A D’ignazio, V Mariottini, G Mura, M Angelini, M Scricciolo, F Barbara, F Tofani, L Nenci, V Borgogni, C Vece, A Sagnotta, L Solinas, M Rossi, S Mancini, R Fruscio, C Ciulli, M Braga, F Romano, M Garancini, F Carissimi, E Vico, Cdg Delle Grazie, S Negri, J Corti, N Tamini, L Cigagna, R Giordano, A Davolio, G Trezzi, T Grassi, C Procaccianti, L C Nespoli, L Pitoni, A Fogliati, G Cordaro, P Passoni, M Fantauzzi, S Villa, M A Scotti, G M Di Lucca, F Benedetti, P Masseria, M Frigerio, M Barba, I Re, M Ceresoli, F Ferraina, S K Adjei Antwi, C Fumagalli, G Di Martino, M L Di Meo, L Bazzano, T Nelli, F Masciello, G Canonico, E Chisci, E Adinolfi, R Borreca, A Damigella, C Di Martino, Gil Mottola, G Fontani, S Michelagnoli, M Fedi, F Leo, R De Vincenti, C Cecchi, L Piombetti, S Giannessi, B Benedetta, M Pagani, L Epis, A Percivale, M Malerba, V Tonini, M Cervellera, J Shahu, L Sartarelli, A Luzzi, E Romairone, S Carrabetta, F Tuminello, F Floris, F Ré, S Marzorati, F Ballari, C Meola, A Filippelli, U G Ribeca, A Viacava, V Lizzi, M Montagna, A Giuliani, N Tartaglia, F Vovola, F Maffei, A Cotoia, M Pacilli, G Pavone, G Berardi, N Depalma, M Maruccio, M G Spampinato, S D’ugo, T Marchese, F Basurto, C De Giorgi, S Garritano, F Manoochehri, F Perrone, I Botrugno, A Rizzi, W Sergi, G I Russo, A Cappellani, S Cimino, M G Asmundo, M G Matarazzo, M Di Vita, P Venturelli, G Cocorullo, F Zarcone, D M Dominici, G Salamone, R Tutino, A Corigliano, R Guercio, C Giuseppe, F D’arpa, G Melfa, M P Proclamà, G Scerrino, I Canfora, M Marcianò, G Guercio, S Barbera, C Amato, A V Agostara, G Pantuso, G Orlando, N Finocchiaro, A Picciariello, D F Altomare, L Vincenti, G Tomasicchio, N Paradiso, A Dezi, E Pinotti, M Montuori, L Siragusa, C Pathirannehalage Don, G Sica, G Costanzo, V Usai, I Carrubba, M Pellicciaro, M Franceschilli, B M Pirozzi, F Santori, M B Busso, A Mariani Ivanikhin, A M Guida, B Sensi, A Divizia, F Blasi, V Giudice, L Orecchia, R Angelico, G Bacchiocchi, L Tariciotti, F Billeci, C Quaranta, C Cascone, M Materazzo, L Toti, T M Manzia, N Di Lorenzo, A Monaco, D Pedini, G Bagaglini, L Keçi, P Lapolla, A Mingoli, G Brachini, B Cirillo, P Sapienza, P M Cicerchia, C Leonardo, E Bologna, L C Licari, M Zambon, G Mazzarella, A Falasca, G Duranti, R S Flammia, V Asero, A Bernardotto, F Scarno, S Meneghini, S Giovampietro, B Binda, A Tufano, V Palombi, E De Meis, M Rocchetti, C De Padua, M Mansi, P Bruzzaniti, P Familiari, S A Nottola, F Fleres, E Cucinotta, S A Biondo, F Viscosi, N Catarsini, C Mazzeo, M Iannello, V Testa, A Gattolin, R Rimonda, F Riente, E Travaglio, F Allisiardi, P Guffanti, F Ferrara, M R Barbieri, A Puzziello, A Mollo, E Donnarumma, F Steccanella, A Oliviero, G Lopez, C M Di Maio, S Olmi, F Di Capua, A Camillo’, A Carresi, M Uccelli, C Rubicondo, R Rosati, U Elmore, F Puccetti, D Socci, M Clementi, A Giuliani, S Tontoli, A Nisi, A Grasso, S Derraj, J Di Biasi, I Tucceri Cimini, C De Nunzio, S D’annunzio, A Nacchia, G Gallo, G Lisi, D Spoletini, S Signore, R Menditto, M Carlini, D Sasia, E Dalmasso, A Trucco, E Olearo, M C Giuffrida, I Morra, M Maione, A Puppo, A Marano, G Preve, B Vercellone, E Frola, E Beltrami, I Peluttiero, G Giraudo, M Migliore, S Armentano, E Alessandria, A Daniele, P Elisa, L Bonino, M Borghese, D Bono, G Canova, M Zago, A Nicotera, L D Bonomo, L Gattoni, N Cillara, A Deserra, A De Lisa, E Coccollone, A Cannavera, R Cabula, F D’agostino, M Deplano, C Chillura, M L Robuschi, A Borzacchelli, C Margiani, M C Murru, G Pellino, E Lieto, F Cardella, A Auricchio, Smc Erario, G Del Sorbo, O Lidia, M D’ambrosio, F Menegon Tasselli, S Mastroianni, F Selvaggi, G Calini, G Terrosu, L Clocchiatti, V Morinelli, C Valotto, F Traunero, G Vizzielli, S Restaino, E De Gennaro, U Baccarani, A Andriani, J Mauro, P Frigatti, E Martin, S Pregnolato, L Driul, A Zullo, G Carcano, L Latham, D Inversini, A Vigezzi, G Ietto, R Marta, A Marzorati, E M Colombo, S Garbarino, N Palamara, M Tozzi, G Piffaretti, M Franchin, L F Festi, M Odeh, M C Fanelli, J Costa, V Pappalardo, A Scorza, M Cannavò, A Ballabio, A Barina, B Franzato, L Rubin, D Brugnolo, O De Simoni, S O Senica, A Ozoliņš, E Vjaters, N Jain, R R Apse, A Truskovs, I A Apse, P Jukonis, J Jukonis, A Tumova, H Sivapalan, Oss Piirtola, A E Berga, H Tumegård, N Samalavicius, V Nutautiene, O Aliosin, V Eismontas, A Dauksa, L Venclauskas, K Jasaitis, D Lazauskaitė, K Urbonas, V Šlenfuktas, I Grikytė, M Jokubauskas, V Simanaitis, Z Dauksa, R Gudaityte, R Riauka, U Krunkaitytė, S Svagzdys, A Šikarskė, J Pakrosnyte, E Stratilatovas, A Piscikaite, G Žaldokaite, T Poskus, V Olekaitė, J Psaila, P Andrejevic, C Cini, K Cassar, S Mattocks, M Sammut, S Mizzi, L Abela, A Micallef, K Pace, B Schembri, R Cini Custo, M Harmsworth, B Farrugia, F Theuma, R Gatt, G Montebello, N Spiteri, J Galea, L Vassallo, L Fava, A Ebejer, K Carabott, K Iles, L Casingena, G Apap Bologna, G Debono, J Galea, M Sammut, S Huisman, W Leclercq, F De Ruijter, J Konsten, D Adramanova, A Nikolovski, A Otljanski, M Kisielewski, K Richter, N Kłos, T Stefura, K Macheta, I Alsoubie, J Tempski, N Wolińska, W Wysocki, E Starek, N G Nowak, R Tarkowski, G Wallner, J Kobak, E Mączka, K Żak, A Ziółkiewicz, K Kułak, E Langa, Ł Łaba, I Krzesińska, M Bobiński, K Frankowska, S Dziurda, J Martyna, J Radulski, O Lulko, J Tomczyk, M Jasiński, P Major, J Rymarowicz, Z Zielińska, K Siuda, M Kęska, P Wites, P Błasiak, A Sierżęga, J Kuciel, D Tomczak, B Molasy, P Nieroda, E Buras, R Gonçalves Pereira, S Patrocínio, S Reis, C Rolo Santos, L Moniz, P Bernardo, C Osório, L Matos, L Carvalho, J Marques Antunes, N Alçada, A Marçal, N Tenreiro, D Martins, C Leal, C Ferreira, R Marques, F Freitas, C Marques, A Melo, S Silva, B Vieira, U Fernandes, B Freire, S Fonseca, M Reis, C Macedo Cardoso De Oliveira, J Mendes, M Carvalho, J Macedo, J Oliveira, C Mexedo, D Sousa Silva, J Davide, E Emanuel Do Vale Gonçalves De Castro Alves, M Ginestal, M D Santos, J Santos, C Robalo, V Valente, J P Fernandes Dos Santos, I Mesquita, B Teixeira, M Marques Monteiro, M I Silva, M Nunes Coelho, M Teixeira, R L Silva, L Lopes, A Ribeiro, C Lima Da Silva, T Correia De Sá, M Martins, M Costa, C G Gil, M Barros, R F Santos, A Silva, J Pedro, M Costa, T A Ventura Antunes, M Pascoal, R Andrade, M Duque, I Prior, M Lemos, P Pinto, P Guerreiro, S Castanheira Rodrigues, E Barbosa, A L Martins, A Pereira, S Vaz, A Fareleira, J Rocha-Neves, L Dias, F Girão De Caires, A Pereira-Neves, A I Oliveira, J P Araujo Teixeira, J Ferreira, M Almeida, M Vb Machado, J Nogueiro, F Gomes, E Campos, F Jácome, C Coutinho, R Ribeiro Dias, J P Vieira De Sousa, E Francisco, C Borges, S Pereira, C Pereira, N Machado, R Calaia, J Pinto, T Corvelo Pavão, L G Santos Sousa, F Cunha, D Melo Pinto, R Pereira, I Dinis, C Ferros, J L Pinheiro, A J Santos, B Barbosa, D Gaspar, M Pinto, D Pereira, N Araújo, B Alves, H Barbosa, D Silva Araújo, M Afonso, B Guimarães, M Campos Coroa, M J Susano, J Azevedo, M Pereira, P Miranda, R Garrido, M Oliveira Mourão, M Ferreira, A Duarte, P Félix, I Antunes, N Fernandes, C Gil Corrêa Figueira, I Figueiredo, M Gututui, S Oliveira, A Silva, F Madeira Martins, A Cabral, C S Rodrigues, B Gama, R Silva Borges, R Lourenço, A C Longras, R Araújo, I Peixoto, A Santos, D Matos, A Lopes, L Claro, D Cardoso, A Martins, M Silvestre, G Sousa, M Santos Bessa, V Cardoso, C Velez, A Machado, R Pedroso De Lima, M I Matias, S V Matos, M Rocha Melo, J Bolota, M Rente, A Santos, M L Antunes, A Bárbara, E Rosin, J Oliveira, S Leandro, M Damasio Cotovio, S Morgado, G Fialho, N Andrade, F Valente Costa Pinto, H Capote, S Morais, M Buruian, F Taré, G Santos, D Rosado, C Costa, T Mogne, N Pratas, B Cordeiro, M Brito, D Salvador, S Carvalho, V Capella, M Carracha, R Alves, C Miranda, F Rebelo, T Teles, M Ferreira, B Cismasiu, P M Costa, S Henriques, M Trindade, J Vaz, M Gomes Vieira, M André, A Macedo, J G Gonçalves Nobre, J Prosil Sampaio, A R Mira, A L Preto Barreira, P Botelho, D Melo Pinto, C Quintela, M J Quelhas, D Pereira, A Cruz, C Mesquita Guimarães, C Pinto, F Maldonado, F Sales, F Marrana, A R Reis Aguiar, L Freire, L Farias, G Faria-Costa, A Castro, T Moreira Marques, G Cardoso, J Ribeiro, M Fragoso, C Silva, M Vasconcelos, M Picciochi, A Sampaio Soares, J Frazão, R Miranda Pera, F Gaboleiro, F Ramalho De Almeida, A Mendes, F Afonso, J Fontaínhas, M Reia, M Angel, C Aguero, M Guerrero, J Dominguez, I Borges Da Costa, L Ramos, J Fernandes, C Assis, F Fonseca, S Carvalhal, A Caiado, F Brito Da Silva, B Miguel, J Moniz, M Pires, B Leal, J Nunes, F Matos Sousa, T Vieira Caroço, M Romano, M Ângelo, A Rodrigues Ferreira, A R Prata Saraiva, F Mano, F Rodrigues, T Branco, S Gaspar, F Neves, M Alves, A P Ferreira Pinto, M Peyroteo, C Baía, M Marques, A M Correia, J O Silva, R Monteiro, P Silva-Vaz, J Gomes, M J Moutinho Teixeira, T Brito Neves, F Meruje, C Sheahan, D Macnamara, J E Linares Gómez, S Clare, A Walmsley, A Tiwari, T Khan, G Dowling, K Hudak, E Craig, A Dhannoon, S Saeedi, J Shah, M Jordan, T Buckley, J Engler, M Reilly, J Ariaratnam, M A Hinn, K Benn, S Petropoulos, H S Hansen, S Browne, S Keelan, M Basta, R Pornsakulpaisal, G Tan, A N Niyaz, N Lakic, T Warhust, Y Q Chang, Y Qaoud, D Mwipatayi, D Kearney, C O’reilly, Y Gamper, R Dhillon, V Patel, K Gallo, H Mulvaney, P Edwards, S Tiller, E Gorecki, M Jordan, S Daswani, V Jones, I Asekomhe, N Clausen, M Khan, A Feldiorean, A Pezeshki, T Khedro, J Beyer, G Byrne, W Irfan, A Poluha, C Fager, L Gilligan-Steinberg, J Yarp, E Byrne, M Lorico-Rappa, A Hassan, T Keogh, W Denning, K Horton-Schleicher, T Deane, A Haren, A D Md Hamsan, N Mukerji, M Dwivedi, Sml Cheah, P X Kwek, S Arshad, A Alzaki, I Cornila, H M Feizal, M Barry, A Semar, P Gorman, A Gordon, C Mcsweeny, N Layyous, D Peiris, S Pan, J Larkin, M M Umar, C Doherty, C Mitchell, H Mcelvaney, Z Sabz Ali, S Adesokan, A Murphy, A Yeo, L Hayes, C Owens, N Crawley, E Macinnis, S Elekes, K E Oderoha, I Mac Mahon, B Moran, P Matreja, K Conlon, P Piankova, M Z Farran, S V Kashyap, M Puntambekar, M Sampat, S Heaslip Owens, A Maheshwari, A Soo, J Butt, K O’brien, M Almutairi, C Mcfeetors, M Kerin, M G Davey, W Murray, A Nasehi, A Graham, C Mathew, S Azam, K Chua Vi Long, S Stafford-Johnson, D Ejaz, Z Siddiqi, Amh Kon, M Parmar, C Peirce, D Shomoye, A Alabi, N Salzberg, V Gallippi, J Aziz, C Melly, H Gill, H W Yang, S E Eltahir Ibrahim, Jsk Rugber Singh, N Bacalbasa, I Balescu, M Muresan, V Dudric, I Imihteev, V Chelaru, T M Băiceanu, M Alexandra, A Bashimov, M Voda, D Muresan, R Varadi, M Muntean, Ș Macovei, R Checiu, D Buf, F Grama, A Chitul, C Bezede, E Ciofic, A Beuca, V Bintintan, D Muresan, A Raluca-Cristina, R Rad, S Șușman, C Suciu, J Abu Arif, A David, M Blaga, B Olesea, D A Butnariu, R R Scurtu, R Rad, P Claudia-Mihaela, M Girlovanu, D Costina, R Drasovean, G Tartiu, S Tache, N Irina, A Streang, A Trif, I Romascan, E Stănică, E Vass, C Cucoreanu, E A Toma, V Calu, O Enciu, A Laceanu, R Maria-Zamfirescu, I M Matache, I Mușat, D C Piriianu, A A Georgescu, A Miron, M Tartalea, N Al-Ugeily, B Bogdan-Gabriel, A Alexiadi, A Zaharescu, C Ciubotaru, I Negoi, M Paunescu, I Iancu, I V Pop, B Diaconescu, P Dao-Zamfirescu, N B Patel, S Rafi, D Popescu, M Girbaciu, A Rădoi, C Breuer, A C Braun, B Stoica, I Gîlia, A Gheorghe, E C Popa, F Brichius, A Bucurica, V M Negoita, M Radu, R Bianca Ștefania, C L Socol, A M Baiceanu, S I Bubenek Turconi, L Valeanu, M Girel, M R Gavrila, M Pirvu, B Morosanu, M Ioniana, A Barbu, R Cornel, B Bogdan, T Bute, L Madec, E Burla, C Balan, E Alexandru, D Tomescu, M Popescu, M Dumitru, A Marinescu, I Petrusca, A Pasca, P Achimas-Cadariu, A Irimie, A Petrusan, M Puscas, S Titu, N M Jiboc, I C Vlad, D T Eniu, P Ciubotariu, V A Gata, A Rancea, C Iulia, D S Morariu, C C Nistor-Ciurba, I Gale, G Lazar, E Bonci, F Ignat, C Lisencu, C Dumitraș, F I Faur, M O Butuza, M C Marian, A Paul Luchian, H Bocse, I C Puia, C Andra, P Pop, S Moldovan, S Ursu, M Sorin, S Lunca, M Dimofte, A Musina, N Velenciuc, C E Roata, S Morarasu, M Velescu, F Mureșan, I Zaha, I M Rusu, B Mircea, D G Tauberg, M Kirov, V Kuzkov, A Nikonov, A Tishchenko, D Kulin, V Mironov, A Litvin, I Gunko, I Mazhega, R Bilenko, G Khrykov, Н Манкевич, M Karpenko, E Zagaynov, A Shilyaev, A Butyrskii, A Tatidze, Z Seytnebieva, A Aliev, M Rumyantseva, A Yanishev, A Abelevich, S Doktorov, K Zuev, A Lazareva, A Malov, D Borisov, Е Борисов, И Андрийчук, Z Galchikova, M Shemetova, K Maltsagova, A Bedzhanyan, K Temirsultanova, D Efremov, A Volkova, Y Frolova, A Minenkova, K Petrenko, A Sumbaev, M Bredikhin, E Bedzhanyan, A Mikhailova, В Тен, Ю Кудрявцев, A Novikova, R Pavlov, M Chernykh, N Boiko, В Данилин, G Stanojevic, B Brankovic, M Nestorovic, N Milutinovic, A Vukadinović, A Vukadinović, B Stojanovic, D Radovanovic, A Cvetkovic, I Radosavljevic, M Sreckovic, B Milosevic, N Markovic, M Spasic, M Pavlovic, D Lazic, R Vucic, B Tadic, B Kajmaković, Z Vilendecic, D Knezevic, U Bumbasirevic, A Stefanovic, P Gregoric, Z Perišić, D Micic, S Ratkovic, J Vasiljević, P R Bulat, V Jovanovic, O Mitrović, J Gunjic, D Potkonjak, N Grubor, M Reljic, I Palibrk, V Milutinovic, M Zivkovic, D Škrijelj, Ž Grubač, S Kadija, R Cerovic Popovic, L Andric, L Aleksić, S Kmezić, I Pavlovic, M Djukanovic, B Milojevic, Z Dzamic, M Petrovic, K Jeremic, I Pilic, B Milosevic, J Krstic, V Šljukić, M Veselinović, N Ivanović, A Janičić, A Jovanovic, D Cvijanovic, I Ladjevic Likic, M Radojevic, J Ćupić, I Dimitrijevic, M Miladinov, A Sekulić, D Nektarijevic, S Pantovic, D Šljivančanin, S Dugalic, A Đermanović, Z Radovanovic, D Radovanovic, M Đurić, S Zahorjanski, V Milosavljevic, D Zdravkovic, B Toskovic, U Marjanović, M Zdravkovic, S Petricevic, B Crnokrak, S Maric, A Sekulic, N Colakovic, B Kovacevic, I Krdzic, J Bojičić, T Sparic, A Ostojic, A Cokan, J A Košir, M Pakiž, S Potrč, N Kavčič, A Ivanecz, M Horvat, T Jagric, U Kacjan, I Perić, E Timošek, I Plahuta, Š Turk, B Crnobrnja, R Kovačič, A Dovnik, L Al Mahdawi, J Knez, J Grosek, T Košir Božič, A Tomazic, L A Suarez Gonzalez, S Busto Suarez, C De La Infiesta García, O Arencibia, D Gonzalez Garcia-Cano, M Laseca, A Martin Martinez, J A Guijarro Guedes, A F Rave Ramirez, Y Gil Gonzalez, A M Hernández Socorro, D Ponce Arrocha, A Cruz García, C Mendoza Rodriguez, E Catala, C S Romero Garcia, A García Trueba, V Georgieva, A I Hernández Álvarez, C Martinez-Perez, E De Valera, J J Gascón, E Lopez Alcina, L Samper Monton, K Aghababyan, A Valero Tarin, C Salinas Lozano, A Cervera, J C Catalá Bauset, B Ramia, F Marques Peiro, J De Andres, S P Iserte Juan, M Peñalver Gaspar, J Gilabert-Estellés, M De La Rosa-Estadella, A Fernandez-Colorado, M Serrano-Martin, A Gasulla-Rodriguez, M Juarez-Pomes, M E Ossola, G Sanz Ortega, J Gómez, M Del Campo, R Corrochano, M Galan Hernandez, F J Molano Nogueira, L Ibañez Vazquez, J Dziakova, A Zarza Martín, S Infante, D Moro-Valdezate, L Pérez Santiago, A I Molina, A Vinuesa Huesca, R Gadea Mateo, L Navarro-Sanchis, P Aracil-Boigues, C Jezieniecki, B De Andrés-Asenjo, Á Zamora Horcajada, F Natal Álvarez, L A Cuellar Martin, M Montes-Manrique, G Cabezudo, T Gómez Sanz, A Herranz Arriero, N Sierrasesumaga, M Rodriguez-Lopez, M Ruiz Soriano, A Sánchez Gollarte, A M Minaya Bravo, C Guijarro Moreno, A Galvan, E González, M Á García Ureña, A Robin Valle De Lersundi, J Ruiz-Tovar, A López Campillo, M Jimenez Toscano, S Salvans, S M Jaume Böttcher, À Godó, G Busquet Raich, C Téllez, J A Sánchez García, M Ribas Ardanuy, P Saavedra, A Sánchez Cabrero, J Clivillé-Pérez, M Durante Escutia, A Rabasa Rodríguez, P Aguilera, C Giménez Francés, M Ruiz-Marín, D García Escudero, M Valero Soriano, M Ramirez Faraco, S Galán Jiménez, P Alcón Cerro, J M Rodríguez Lucas, M Carrasco Prats, E Peña Ros, Mdp López Sánchez, V J García Porcel, M Tamayo, J M Muñoz Camarena, R Albarracin Garcia, O Molina, P Moreno Sánchez, I Jiménez, P Pastor Perez, M B Agea Jiménez, D Candela Mas, M Artés Artés, J A Benavides Buleje, F M González Valverde, P López-Morales, A Sanchez, E R Bobadilla Romero, M Allue, L Ponchietti, R Latorre Tomey, D Torres Ramos, L M Jimenez-Gomez, S Valdés López-Linares, Y T Moreno Salazar, J Soto Galán, C Moreno De Alboran, A Prosperi, J J Osma, A Blanco, M Sánchez Rodríguez, E Valdivielso, Á Cejudo, C Perez Carpio, F Vasques Seabra Águas, E Sánchez Martín, S Pérez-Ajates, E Macarulla, A Álvarez Torrado, C Galmés, M Artigot, J Marin Garcia, J R Oliver Guillen, A López De Fernández, B F Fernández-Velilla San-José, D Ambrona Zafra, S Pérez Farré, J Ortega Alcaide, L Codina Corrons, E Sisó Soler, F D Gómez Báez, G López-Soler, E Gutiérrez Pérez, S Aix Molina, M Vallve-Bernal, A Varona Mancilla, Á M Aldama Martín, R Casanova, L Sevillano, J J Muela, M Rodrigo Rodrigo, I Goujon, C Fructuoso Iniesta, S Pérez-Bertólez, E Monclus, L Fernández, M G Silva Hernández, F Vicario, P Garcia Pimentel, R García Álvarez, N Bouzó Molina, Á Ramiro, Z A Calderon Barajas, R Sanz, Z Xia, P Rodríguez, C Sarrais Polo, A Martínez López, C Estrada, P E Gómez De Castro, D Fernández Martínez, L J García Flórez, I De Santiago Alvarez, L García Alonso, G García-Santos, L A García González, B Carrasco, D W Silva-Cano, P Del Val Ruiz, G Martínez Izquierdo, A Corteguera, J Iturbe, E López-Negrete Cueto, A Cembellin, C Ramos Montes, G P Ibero Casadiego, M Prieto, I Villalabeitia Ateca, T Pascual Vicente, B Villota Tamayo, A Perfecto, S Mambrilla, I L Ramos Del Moral, V Jiménez Carneros, P Pastor-Riquelme, A Franco Lozano, L Alonso-Lamberti, M Lozano, J García-Quijada, R Zhang, A García, M M Martín, M Yeh Ahumada, A Carreño Pallarés, Á Pérez Rubio, R E Goran, N Gómez Diez, G Zomeño Bravo, J C Bernal-Sprekelsen, G S Martínez Fernández, R Marquina González, C Toribio-Vazquez, A Eguibar, H Perez-Chrzanowska, P Serrano Méndez, S Valderrabano Gonzalez, F J Reinoso, A Yebes, M B Alonso Bartolomé, H R Ayllón Blanco, I Cristóbal, I Pellicer, R Arenal González, J D Zafra Angulo, V Duque Mallén, C Gracia-Roche, I Gascon Ferrer, D Aparicio-López, M Antonio, M Herrero Lopez, S Saudí-Moro, M Á Gascón Domínguez, T Gimenez Maurel, B Matías-García, N Morales Palacios, M Diez Alonso, A Gutiérrez, S Soto Schütte, M Bru-Aparicio, R Jiménez Martín, A Sanchez Pellejero, S Vázquez Valdés, C Vera Mansilla, A Quiroga, F Mendoza-Moreno, L Diego García, E Serrano Yébenes, F Mañes Jiménez, I Lasa, E Ovejero Merino, L Casalduero, Y Allaoua, A Blazquez Martin, D Córdova García, R Alvarado Hurtado, P Laguna Hernández, F Garcia-Moreno Nisa, H Juara, C Aldecoa, F J Tejero-Pintor, M García, E Aguado Saster, E Ruiz De Santos, A D Bueno Cañones, S Pérez Fernández, M G Alija Garcia, R Urruchi, R Rioja Garrido, M Madrid, A Bordell, I Arranz Chamorro, P Rodriguez Cañal, D Pacheco Sánchez, P Marcos-Santos, S Veleda Belanche, J L Maestro De Castro, M J Blanco García, E Laita Jiménez, L Leal, L Vaquero Perez, C Barbosa, M Ramos Carrasco, I Garcia-Saiz, P Rodicio, F Acebes García, A Lizarralde, A Sanchez Diez, E Ferrer-Inaebnit, J J Segura-Sampedro, M Alfonso Garcia, Ggc Gutiérrez-Cañadas, A Oseira, N Torres Marí, J Loyola, B Villalonga, P Camporro, N Pagès, E Colás-Ruiz, J A Cifuentes Rodenas, M Castro Suárez, R Moll-Amengual, J Mata, J Fernández Manzano, B Gómez Pérez, J Gil Martínez, Á Cerezuela Fernández De Palencia, A Aliaga Rodriguez, P Gómez Valles, A Delegido García, E Gil Gómez, M J Montoya, A Navarro-Barrios, V Cayuela, J J Ruiz Manzanera, M I Jiménez Mascuñán, A I Gutiérrez Fernández, I Sánchez Esquer, P J Gil Vázquez, D Ferreras, A Balaguer Román, F Gómez-Bosch, F Alconchel, A Romero, V M Durán Muñoz-Cruzado, C J García Sánchez, A Tejero Rosado, B Ruiz, Mdc Roman, J J Rubio Fernández, Á De Jesús, M Ostos Diaz, C Quintero-Pérez, A Sánchez Marín, M Alvarez Aguilera, M Leal Mérida, I Ager, J Sancho-Muriel, M Frasson, B Castro, P Guerrero, M Nieto-Sanchez, Q Cruz, H Cholewa, L Hurtado-Pardo, D Plazas, M Serrano-Navidad, J Castillo, R Ballestero, V Valbuena Jabares, C García, N Garcia Formoso, E Herrero Blanco, N Suarez Pazos, C Cagigas Fernandez, M Gomez Ruiz, Y T Benic Yoris, M Merayo, M E Gonzalez Fernandez, S Alonso Batanero, J Rodríguez Gómez, A Landaluce-Olavarria, B Estraviz, I Markinez, D Gómez, L Fernández Gómez Cruzado, M González De Miguel, J M De Francisco Rios, A Urigüen, A Gómez Del Pulgar, E Espin-Basany, A Gil-Moreno, S Bellmunt-Montoya, C Dopazo, S González-Suárez, R Blanco-Colino, P Olid Mayoral, M González Antúnez, E Del Barco Martínez, Y Bejar Dolcet, D Gil-Sala, C Marrero Eligio De La Puente, L Sánchez Besalduch, M Barrio Zaragoza, J F Lopez Lozano, C Olaria, S Catalan Sanz, E De Ciria, L Segura Farran, N Montes, J M Zanca Gómez, I Puig Escobar, M Armengol Alsina, M San Nicolas, M Escura, M Sánchez, M Rivas Agudo, L Aalouf, D Maya Salas, L Hernando Marín, N Umpiérrez Mayor, J L Sanchez Iglesias, Y Fernández Henriquez, I Feixa Molina, M J Gomez-Jurado, V Bebia, M Racine, M Sauvain, A Mujcinovic, D Bolla, Y Dimitrov, N Arenja, C Riboni, J N Marx, M Kalisvaart, C Andreou, P Brandts, U Pfefferkorn, L Eisner, U Dietz, J Gass, J Metzger, A Scheiwiller, V Kremo, D Gero, M Bueter, F M, R Schulz, F Mongelli, I Soave, D La Regina, C Canonica, F Sabbatini, P Gaffuri, S Spampatti, C Santarelli, M Di Giuseppe, M Marengo, S Passoni, G Dellaferrera, D Provenzi, L Bernardi, A Papadia, A Cristaudi, M L Gasparri, P Aurora, S G Popeskou, P Christian, M Antonilli, F A Scalvi, V Sevas, G Palumbo, L Maoloni, C Cencetti, G Pagnani, M Hitz, J Dürmüller, S Däster, G F Hess, Nle Aegerter, P Sedlaczek, S Soysal, J Zeindler, F Angehrn, L Müller, S Taha-Mehlitz, F Haak, F Nocera, T Karli, I Lazaridis, A Tampakis, A Lalos, U Friedrich, B Wiesler, N Varathan, R Frey, G K Kurtoglu, A Aghayeva, C Turam, G M Kurtoglu, M Maarouf, B Baca, S D Ilhan, Y O Koyluoglu, M E Seker, M Ulufi, M Erkaya, Fdb Kılıçkan, S Bayrakceken, B B Ozmen, B Togay, M Doğan, T Çetinkaya, E Tuzuner, T Karahasanoğlu, I E Yavuz, I A Bilgin, S S Kekik, C Turam, E Dönmez, M Dikeç, N Ramoğlu, N Yurtseven, O Takmaz, Ö Orhun, N Karadeniz, O Saylık, E M Uğur, E Ada, A Durbas, I Hamzaoglu, K Uzun, M Gungor, I Y Gebedek, E Ç Hayırlıoğlu, M Halıcı, G O Ceyhan, A U Mutlu, E Aytac, M Gulmez, E Capkinoglu, T U Yilmaz, A Ozer, S Keçeoğlu, Z C Eraslan, V Ozben, O Dülgeroğlu, C Saraçoğlu, M Erkan, C Uras, H D Copur, N U Dogan, E Topal, I Demirsoy, D Korkmaz, U Can, D Buğra, E Sobutay, H Çakıt, E Ergün, S Zenger, C Bilgic, B Gurbuz, K Sünter, I Gecim, S Sefer, B Inceöz, M B Genc, A K Uygun, E Yücel, A Barcin, Y Altinel, Y E Aktimur, S Meriç, K Özdoğan, A I Sayar, A G Durmaz, I Çakır, E S Ünlü, G Kiran, A Dal, E Ozkan, S Kalkan, M M Öncel, Ç Çetin, G Yılmaz, H Karimi, Y Iskurt, E Yardimci, M Eker, A O Balkan, H B Gönül, E Herdan, M Ertugrul, T Takmaz, S S Kilinç, M S Pinar, F Yıldıztekin, E C Coşkun, I Olgun, G Ince, O Isik, M Şen, R Özata, B Eroğlu, B Büyükpolat, G Ishakoğlu, D Özen, M O Aktaş, B Kılıç, T Yılmazlar, M S Koçbey, N Işıklı, R G Yıldız, T Baghirov, E Uygun, M Yugaç, E Keskin, B E Bozkurt, S Tas, Ö Ö Türkmen, B Ata, B Alan, N Işık, T Göver, T Bisgin, E Özalp, S Sökmen, B Manoğlu, C Bakar, O Bozbiyik, U Zorlu, N N Zengin, B E Baki, E S Akbulut, S Tunali, B Kutlu, Y A Oğuz, B Küçükateş, E Yildiz, A N Sakmar, T Yoldas, Z E Akgün, M A Korkut, C Çalışkan, E Aksit, E F Aksalman, A O Koçoğlu, F Basci, B Yigit, O Ilhan, R K Liman, M Uzun, I Ağaçkıran, C Şahin, S Leventoğlu, Ö Kubat, I Genç, A Oyanik, F I Gurbuzer, N Satilmis, S Cam, M Ekinci, M Gönül, D Chasan, Sgf Zara, Ş Sök, A Yalcinkaya, E T Acıpınar, U Timurçin, N B Afzal, Sha Gillani, S Gillani, S Yazıcı, O Cennet, M S Suer, Ö Kaya, E Z Uslu, A Tanrisever, C Yildirim, I N Gunenc, B Gül, I Özkal, H I Tileklioglu, M A Korkmaz, I Şirin, U Özbayrak, A Mıdık, E Domaç, A Karakoç, S Urek, A Yıldırım, M Ugur, M Doğru, E R Arslan, B Ular, M E Duymuş, A B Turhan, T Dogan, M B Ozkan, D E Benek, M Arslangilay, M B Yildirim, Y Yazgan, H N Tıraş, B I Şahin, M A Çapar, C Tatar, A Arı, O Batikan, M Güler, A E Naycı, M M Sevinc, H Şevik, C Oğuz, O O Karagülle, U O Idiz, A Demir, E Çakır, C Cakir, S Doğan, I Yıldız, M Gürlük, M B Cengiz, M Toptaş, O Akay, R Kaya, E Aydin, M Ç Çakıcı, A Yildirim, Ö Efiloğlu, B Demirtaş, A Iplikçi, G Atis, F Keser, M Culpan, M Çiçek, A Tahra, M K Akalin, A Izgiş, M Soytas, N Okkabaz, A Saylar, E Onaran, A E Askin, E Hashimov, S Bektas, K Sabuncu, A C Alagöz, I Karatas, Afk Gok, E Simsek, Z I Altunay, E Koç, Y Iscan, I C Sormaz, B I Yabaci, M S Ertürk, M Nazli, I E Saglam, E Erginöz, M F Ozcelik, M Gokden, C Guler, F I Gunaydin, S S Uludağ, B Ibis, H O Bozkir, M B Karaca, M C Ulucay, K B Oner, S Akbas, F Z Calikusu, H Akcan, S Ugural, A K Zengin, Z Ozdemir, I Demirbas, A Guner, M D Tepe, N Kıralı, B C Karabağ, D Pehlivan, A N Yuzgec, B Yıldız, B Akin, M Aktaş, E Karabulut, H Cepe, H Küçükaslan, S S Salih, M Uzun, Ö Yücesan, M E Reis, M Ulusahin, K Aşcioğlu, E Tufan, Q Saleem, K Saracoglu, M Ozbilen, A Kale, M E Geçici, Y Bulun, Ö Uysal, A Atay, B Şuataman, C Guducu, T N Çinar, H Kul, D Canpolat, M Ipekoğlu, M Aydemir, F Karahan, K Hizli, B Kaya, O C Yenen, B Gümüş, K Turmuş, S Vatansever, I Candan, S Sucu, E Balik, E Bozkurt, I H Özata, E Ozoran, T Tüfekçi, S N Karahan, A F Sarıkaya, O Agcaoglu, S Danacı, M E Ulutaş, I Hasirci, K Arslan, Ş H Metin, A Yılmaz, E Turan, G Şimşek, A Kılınç, M E Şahin, S Ozden, Y A Bayraktar, A S Maden, A Şahin, N Acar, O Erşen, E Balcı, H Yaldız, C E Guldogan, M M Ozmen, E Gundogdu, M Moran, B Celik, E Sivrikoz, K Karabulut, A C Dural, T Ikizoğlu, H Aydede, T Yalçın, A Dalkıran, H B Yapici, T K Uprak, Y Tatar, Y Aksahin, C Aral, S Z Cetin, D Artvin, Z Tatar, I S Karakuş, S Çatal, B Nalbantoğlu, S Bettar, Z Düzyol, B C Demir, M F Kırcalı, M E Taşcı, F Secil, E N Akkaya, I Kayılıoğlu, B Hekmatjou, A Çağlayan, M A Dadaşoğlu, C Fergar, M S Beden, E Kayhan, I Arslan, G Z Koçyiğit, B Beyoğlu, C Varan, U Sungurtekin, H Sungurtekin, U Özgen, B Çetin, A Pergel, M Uyan, E Erata, E Aldhahebi, B Aslan, Ş Kabalı, F Köse, I Keşaplı, U C Dulger, F Altintoprak, M Akçay, E S Cünük, M B Kamburoğlu, I F Küçük, M Yigit, E Sabuncu, A T Harmantepe, B U Aka, E Baş, T Yavuz Akça, A C Sarı, E Colak, M Candan, M E Kara, A B Ciftci, K Yemez, M S Uyanik, S Polat, G O Kucuk, S Ocak, Y Tosun, E Unal, C B Ofluoğlu, C Üstbaş, Ö Gangal, A N Sanli, S Sayır, C Karslioglu, I Ulusoy, A E Tufan, U Demir, M F Celayir, E Baran, A B Aksoy, D B Fırat, A Yüksel, O Güven, I Ertaş, Z S Kuzu, H M Köksal, M Gok, M N Görgün, P Yazici, S Ömeroğlu, B B Özdemir, E Kabul Gürbulak, M Ajredini, I E Cakcak, I H Atakan, A Göztepe, O Budak, E O Aydoğdu, E Erdem, C Yılmaz, J Özdemir, B Akin, D Alkan, E Cengiz, C N Donmez, E Akyüz, Y H Ergun, M G Ozer, Ç Ak, F Bolat, E Kasapoğlu, A Tatlisu, Y Yorulmaz, A Enez, B Ozkan, Z E Yilmaz, S N Altin, B Gunes, B Kaban, O Korkmaz, I B Demir, G A Öz, A B Tuluce, Z N Turkmen, F Yildiz, B Kandil, A Ulkucu, G Kıral, D Yavuz, Ü C Köksoy, Z C Kus, M B Erten, M Mutlu, F Hökelekli, T Aslan, Ş Orçan Akbuz, E Durmuş, T E Gökçek, H S Ulgur, O F Ozkan, H K Karakullukcu, A Yıldız, M Ceyhan, M Ş Çelik, M Kalın, E F Kirkan, A Demirbilek, M N Çelik, Ö Karbuş, S Demirli Atici, B Abud, H Erdinç, K B Ön, B Sevinç, N Damburacı, E Altiner, B Özdemir, Ö Manisalı, G Ural, M K Topal, T Can, S Ercan, S Kaynar, O Emanet, M Javadov, U B Demir, A Erdem, F Demircan, M Yelmenoğlu, E Seçen, B Alkışer, F Arık, E E Kaya, D Akkad, D Karaçam, U Karacam, M A Yücel, A Hatipoğlu, N Mumcu, O Karima, M B Dal, M Aljobbeh, J Montaser, M A Kara, F A Gultekin, O Deniz, N Kavak, B Kum, M M Ecir, Y G Yavuzer, Z Sezgin, U Koçdemir, B Yirmibeş, Ş Atalay, A May, S Varna, I Aydoğdu, A Abdel-Fattah, G Ramsay, M A Bin Badekruzaman, M Karvelyte, M Siamisang, S Clunie, M Mikalauskaite, A Nessa, C Joshi, N Jodeh, R Zafar, J Miller, R Basharat, T Singh, O Weston, R Loy, Ehc Tsoi, A Chandiramani, R J Sumarlie, L Mitchell, M Yousif, M Qadir, M Gannon, Eyh Tong, J Tsang, M Elmarghani, J Kaczmarek, J Luangboriboon, R Mcewen, B Tse, M C Wong, Z X Wong, T Diffley, O Jaruwattanapradid, N Ng, L Mchaffie, C Wright, D Joyce, V May, S Emmanuel, A D’costa, A Kumar, C Hanganu, S Gourgiotis, A Townson, C Y Williams, E Baines, L Smith, N Elks, N Simon, R Chintapalli, A Banerjee, S Bhogle, B Ryan, D Maghsoudi, E Clifton, T Nott, H Vickers, G D Stewart, Z X Zhong, K Ioannou, Ckh Ne, R Conci, E Fitzsimons-West, X L Ling, R Patel, R Sanghera, I Dokubo, I Seago, O Fairhurst, B Amin, M Aniq, E O’keeffe, S James, M Chowaniec, L Rutigliani, M Hu, A F Ferreira, M Kalogeropoulou, G Hui, L Coakham, S Healy, K Gilanliogullari, G Nishimura, M Choi, R Lunevicius, D Aje, R Mcnicholas, C Newman, E Bollen, A Kumar, I Ferreira Xavier, L G Baloch, N A Koduah-Sarpong, G O’sullivan, S Saseendran, A Bin Sahl, E Murphy, H Hussain, A Nicholls, S O’dolan, A Gidwani, E Patterson, L Loughrey, K Donaghy, K Hana, M Monaghan, S Ni Shandair, N Kulasingha, N Gormley, M Mcglinchey, E Dunlop, B Dunwoody, S H Lian, S C Jayasangaran, E Logue, P Goswami, P Ann Jacob, C O’kane, A S Ab Razak, K Govender, E J Mccann, S Perry, S Mckendry, A Pachchigar, A Singh, S Dindyal, S Pendyala, V Venkateswaran, L Alhoussan, F Feil, T Hughes, Y Gerçek, M Choudry, O Haidar, T Rujeedawa, T Speirs, J Odendaal, J Chu, A Shahab, S Ranjithkumar, V Pillai Rajendra Prasad, T Linn, J Clark, M Sharma, S Lockwood, S Chawla, R Deshpande, N Long, J Rees, M Kobetic, D Fawcett-Till, O Ferris, G Harrison, T Hibbs, Ctw Tsang, L Hurley, T Jichi, C Badrinath, L Applebee, K Ecott, T Sullivan, B Piecha, A Jagadish, Z Sajjad, R Griggs, S Joyce, R Spurgeon, M Ali Khan, M Hobrok, R Roberts, J Mckenna, D Davies, P Eaton, L Bishop, S Magaway, K Denholm, S Doyle, R Deshpande, M Salter, V Chan, K Mockford, J Heinz, I Ahmed, M Coverdale, R J McKillen, M Maguire, N Mclees, J Lau, L Mcguigan, A O’Neill, D O’Hare, C Beggs, O Owolabi, J Hunter, J Kinross, J Kotecha, R Doherty, V Patel, E Wagner, L Hodges, H Hassan, K Sribaskaran, K Pouris, S Jain, A J Kim, S Gill, S Nand, O Toutouza, A Joshi, L Thornley, R Chen, M Ogunjimi, A Mahmood, R Chong, M Yanai, D Bae, J Dhaliwal, A Lovejoy, A R Akhbari, N Mayor, C Kontovounisios, Shk Yap, M Damarla, B Ooi, M A Ng, A Vargas Zhang, S Limbu, K Nyamakope, Y Agarwala, N Zingas, C Cleasby, E Smyth, J Drmota, Qzc Yang, C Vedi, S Samarendra, V Nayak, S Rajendra, M Choudhry, K De Stadler, S Bandyopadhyay, G Bond-Smith, O Collart, K Baffour-Awuah, R Shah, J Mcnamara, G Tadikamalla, B Wilson, C Cossins, H Li, L Ismail, H Soleymani Majd, E Chang, A Jallow, S Baldelli, P Alberti, O Grant, M Doody, Z Borawska, A Bowman, H Clay, Y Petit, J G Kimani, M James, S F Hussain, A Nezhentsev, M Emmerson, R Vijjhalwar, G Shaw, C Holmes, Y Ying, L Farache Trajano, A Anis, J Dequaire, A Hunter, R Danvers, M El-Nemr, C Hammett, M Pikoula, S Thompson, B Lander, S Dierksmeier, N Sadeghi, R Suribhatla, R Ahmed, S Pandit, W Thornton, T Thornton-Swan, A Johal, M Khan, A More, Y Tilahun, A Hauperich, R Gidda, I Vorley, Z Khan, M Sintler, A Van Den Broeck, M Georgiou, N Eardley, H Salem, S Yemparala, L Whittle, C Jagger, K Turner, L Vernon, K Aldred, P Manokar, A Moore, N Mistry, J Zgliczyńska, C Haylett, A Adeyanju, E Headford, R Khanna, M Deef, T Durkin, E Trayling, R Cowell, Z Khan, A Turner, K Noureldin, W Down, A Cyril, C O’halloran, M Shirke, M Epanomeritakis, A Karnati, G Sreejith, C Sinton, L Oliver, H F Ali Azamatullah Khan, E O’kane, J Trouton, B French, I Campbell, R Curran, C Brines, A Mcdermott, N Yang, S Vig, S Chowdhury, R Valecha, R Lau, M Nafis, S Thavanesan, J Marks, L Akaje-Macauley, N Patel, E Beard, A Nanda, C Mills, A Cheung, J Amalendran, L Todd, S Smolarek, T Warrener, S Sen, A Mudehwe, S Khan, B Wisden, G Lau, L Schanzer, Z King, K Giridhar, M Reed-Embleton, L White, P Filippidis, L Lee-Smith, O Carless, C King, M Herriott, N Saju, Y M Tin Maung, A Deligianni, A Mumtaz, S Khan, H Hill, S Sahdev, I Thomas, A Shah, S Saji, J Hargreaves, R Khan, A Tharani, R Ullah, J Ludgate, S Shrestha, E Bota, A Abdullah, M Allison, Z Patel, A Clark, I Suchett-Kaye, B Stubbs, B Holmes, A Santaniello, T Watkinson, A Crimmins, M K Gupta, S Abraham, A Bellringer, R Clowes, D Chatzopoulou, A Kiran, J Eid, N Rao, J Caterson, F Soggiu, H Malik, I Jose, K Kavallieros, C Rizk, B Thummala, K Li, A Goel, R Pantula, J Toh, M X Fu, C C Ho, K Vivek, E Owen, A Day, A Jamieson, H Hassani, T Thavarajah, M Vivekanandan, A Muneer, M Chauhan, D Veeramootoo, S Ali, J Dosanjh, F Newing, E Jose, H Chauhan, A Kulshreshtha, E Thomas, P Patel, R Chhabra, B Sajan, S Ragavan, R Hariharan, I Lam, B Fleet, N O’hara, A Wright, V Reddy, H Darweesh, A Khan, S Handa, D Kewada, F Rana, H Williams, F Bombieri, N Shah, D Pestotnik Stres, A Menon, L Selvam, A Pusok, C Street, M Zohdy, B Aguirrezabala Armbruster, I Okoye, L Potts, M Fayyad, K Bajaj, H Alfa, N Sivakumar, N Duncan, C Roxburgh, L Huang, K Lukito, X Huang, S Baig, C Y Chan, R Philip, L Shaheen, J Dodds, H X Yeow, M Devindran, Z C Sia, J H Park, B Furze, N Yung, M Vipond, M Jones, F Asekun, A Dembinska-Kenner, R Saleh, E Larkai, J Harris, M Cherian, H Louden, V Bisbinas, A Behl, R Hughes, R Smith, Z Zaman, O Hoskyns, H Carlton, C Thorn, M Tourky, J Tan, K Sen, J Elsey, J Bevan, N Ko, S Kalidindi, M Rooney, K El-Boghdadly, S Zafar, A Wyncoll, P Morillon, M Sennaraj, H Mahfouz, A Afzal, R Sibal, T Suji, H Headon, A Abdul, S Ahmed, J P Mcnally-Reilly, K Omran, C Mulcahy, M Aftab, M Haghighat Ghahfarokhi, M Farhangi, W Y To, M Ho, T Patel, A Siu, H Choudhry, M Huntley, Z Hussain, G Wong, R Maguire, M Khan, A Potts, M Ahmed, G Ramesh, O Kolade, M H Siddique, E Griffiths, M Qureshi, M Hoque, S Laulloo, K Das, N Waheed, Tyt Tang, R Ahmed, R Habib, R Vyas, S Watson, K Theodoropoulou, M Siu, N Yu, N Islam, J Burnett, A Besso-Cowan, D Hirani, I Zsolnai, Y K Chen, O Dupere, L Keitley, C Park, I Verma, J Chan, J Tavner, M Nicolaou, Nys Lee, R Hegy, Rby Lee, Kyl Yi Lun, D Reyes, S Dong, J Thornton, P Eaton, L Bishop, S Magaway, K Denholm, S Doyle, R Deshpande, M Salter, M Weir, A Gibbs, A Al-Shaye, M Alwahid, A Tait, S Smith, A Doye, K L Law, T G Groot-Wassink, G Geller, K Seebah, O Cox, M Kalogeropoulou, A Shetty, Dcj Oh, E Lee, B Packham, M Aarons, K Saadeh, J Mui, R Huynh, M Eid, N Honey, J Kaur, R Hand, L Lai, C Koubaesh, H Maqsood-Shah, J Suresh, O Collart, G Bond-Smith, R Shah, J Mcnamara, G Tadikamalla, B Wilson, C Cossins, H Li, H Soleymani Majd, L John, E Chang, J G Kimani, A Jallow, S Bandyopadhyay, S Baldelli, O Grant, M Doody, Z Borawska, A Bowman, C Foster, H Clay, Y Petit, M James, S F Hussain, A Nezhentsev, M Emmerson, R Vijjhalwar, G Shaw, C Holmes, Y Ying, L Farache Trajano, A Anis, J Dequaire, A Hunter, R Danvers, M El-Nemr, C Hammett, M Pikoula, S Thompson, R Shah, S Dierksmeier, N Sadeghi, B Lander, R Suribhatla, R Ahmed, S Pandit, W Thornton, T Thornton-Swan, A Johal, M Khan, A More, Y Tilahun, A Hauperich, R Gidda, I Vorley, E Lewis-Orr, S Reilly, A Bhatia, K Goves, M Blesson, L Purser, L Cobb, M H Sarker, N De Sousa, S Syed, N Rajendran, J Tan Sue Wei, G Navakumar, V Butnari, M Senthilkumar, Z Q Chew, L Zekaite, S Paranietharan, M Haque, S Balenthiran, K J Chin, S Ahuja, T Moothathamby, F Moniati, Y Z Kong, Ejh Lee, L Sharma, P Found, Y Sivakumaar, H Senior, N Simeen, A Arora, A Chu, R Mizori, T Beazer, N Patel, J M Dudziak, T Squeri, F Weston, M Zhou, J Van Ross, S Tian, J Bedford, R Lam, F Karami Tireh Shabankareh, H Donkin Everton, K Wilson, A Richens, D Bragg, A Akbari, A Awodiya, S A Osula, F Gerges, I Gerogiannis, L Daniels, S Seth, Z Baxter, E Nour, L Fitchford, C Perrott, M Abuelgasim, J Flanagan, S Gillani, H Lewis, O Dunscombe, P Steven Goodwin Moughton, A Mahmood, J Hirniak, A Moses, P Kapsampelis, A Ahmed, D Burke, C Anderton, E Lee, M Conley, L Khan, R Surti, F Waseem, M Sharma, A Imran, R Motiwale, B Singh, S D Sa, S Charuvila, M Z Lorgat, R Chowdhury, P Gogineni, A Finney, A Tzortzi De Paz, S Panesar, A Sidki, C Warwick, S Kadambande, J Patel, A Rao, A Sharma, S Ramewal, P Ramesh, M Norwood, P Mehta, M Bhatia, S Tiwana, E Foote, F Rushton, S Asi, R Notaney, R Sinha, J Vibhishanan, S Gupta, R Conci, R Chintapalli, S Ahmed, J Sagar, N Ragge, S S Rizvi, M Ashraf, N Cirocchi, R West, E Obiri-Darko, Y Rai, S Hussain, N Ul Ain, F N Amir, C Smart, R Melomud, D Saad, S Bengeri, S Kuna, M Chaudhary, O G Olanite, F Khan, S Nausheen, R Dodds, H Boyden, D Donnelly, O Adegboye, L Osborne, S El-Barraj, J Shukla, N Raza, A Khalid, D Agrawal, K T Kyaw, S Borhan, G Pangrazi, I Ioannou, A N Ahmed, G Savvides, M White, S J Puthur, S Bridgewater, N Waraich, R Bryce, R Khosla, L Penhaligan, A Desai, Z Ehsan, O Mostafa, Y Kamel, B Keeler, R Rajivan, V Omar, S Gamadia, D Rana, S Srikumar, A Heidari, A Ahsan Akhtar, A Tarafdar, J Barry, D Davies, A Curr, I Jimenez-Reid, J Mckenna, E Garry, N Lallmahomed, K O’leary, E Barker, D R Jones, R Tabatabai, J Garg, E Henshaw, M Abdulshafea, S Prabaharan, H Wiles, R Bamford, F Blest, L Acquah, H Simpson, J Guerrero Enriquez, H Pringle, M Shahid, O Wharf, M Kumar, K Buadooh, M Hanson, I Mutanga, A Hardy, H Usman, S Shams, N Schottler, A Garg, V Sarodaya, S Ali, I Cullen, L Pregil, M Fernandes Silva Ramos, Y Hao, A C Das Chagas E Silva, S Sellahewa, J Li, J Cox, P Sinha, Ijj Lee, E Voniati, H Phillips, O Mooney, M Sockett, P Patil, M Elsllabi, Wkm Chan, L Grimble, H Richardson, A Johnston, T Kouli, Jfm De Sousa, Y K Goh, C Grant, L Martin, Z H Peh, T Yap, Etw Tang, Hym Lai, T Berry, M Rokia, E Tennant, I Lee, M J Rahman, A Kamal, S Ali, H X Hau, I Harten, M Eloofy, C Caldwell, R Leckie, F Tasnim, K Ravintharan, J Mcauley, F Cameron, R B Veerni, K Y Looi, A Ismaili, R Gresz, V Chong, L K Tan, K Singh, S Ashburn, R Shantha Kumar, M Kamal, H Kamal, I Aziz, P Stather, L Mylvaganam, E Deliyannis, B Tompkins, E Sikorski, F Harris, I Sanders, M Fakhrul-Aldeen, K Cross, A Tahir, P Yim, I Mayne, S Mahapatra, C E Ng, A Ortega, S Supparamaniam, M Bowman, M Sadler, M Rogger, A Gendia, J Ahmed, P Sivakumaran, C Brick, E Ansong, H Ha, A Sharma, N Wilson, B Manavi, L Zeze, N P Gupta, S Al-Hassani, C Williams, M Ramesh, B Shukla, E Drye, H Hussain, E Ghatauray, Acw Tan, F Adamu-Biu, J Arora, T Mcallister, S Fairclough, A Economou, S Utulu, K W Fung, I E Epanomeritakis, S S Zaman, E Olszewski, P Ballesteros, E C Okpii, E Walkeden, C Macklin, S Jamil, S Zhen, O Ahmed, A Saravana Kumar, H Uddin, R Hakim, M Mossanen Parsi, O Awoloto, K Rowe, R Mashadi, Z Mohammed, L C Chong, S Fairlie-Vogt, O Russell, W Ozarek, A Little, C O’farrell, R Miller, J Froud, K Thippeswamy, R Thomas, A Fitzpatrick, S Yoganathan, M Dunlop, K Ngai, T Liddell-Lowe, A Barrow, L Sharma, P Found, R Mizori, S Rabas, R Al-Housni, M Zhou, A Marton, P Chen, H Hasan, Cfb Chan, W W Win Mar, A Kisiel, E Griffiths, M Yusuf, A Sinha, J Blenkinsop, K Rawlings, T Chaudri, G Westland, H Umar, S Lee, J Cherian, Z Gurhan, T Chaudri, M S Sheraz, Dmi Khan, A Kucukmetin, D Malone, C Betts, M Ali, M Giblin, L Nowicki, P Korompelis, O Farley, M Fouweather, C Ioannides, H Yilmaz, R Landais, E X Ngeyu, C Erinjeri, D Badran, P Glen, J Aamir, A Aziz, P L Su, I Choong, M Reece, A Kulasekaran, Tns Tengku Saifudin, J Luckhurst, R O’hare, R Smith, Y T Siu, P H Kwok, V Sood, A Imran, K Fatima, Z Munir, T Kisova, N Rajendran, F Islam, E Teehan, H Sadik, U Patterson, N Rahman, H Harrisrhaj, D Govardhan, S Rajesh, Z Mumtaz, O S Ghori, H Nawaz, A Lysomirski, V Pikoula, Rmab Qadir, A Jones, Y Chiang, H Morsy, C Pazaiti, A N Asardag, I Ali, J Althonayan, A Badri, J Evans, E Chung, L Harriss, L Sinan, C Maxwell-Armstrong, A De, R Altman, F Varghese, W Chua, S Ahmed, M Javed, R Aljubure, A Bradley, S Moug, Oeh Kemmett, I Underwood, I Fitzpatrick, L S Wong, B Vakeesan, K Potter, T Varghese, S Daren, A Hazrin Fazail, S Farajzadeh Asl, H Nautiyal, A Saadeh, I Vial, R Karimi, A Akthar, A Egiz, R Masood, A Qadri, T Chawla, H Yoon, X Liu, N Battersby, E Davies, R Chhetri, R Mclean, E Leaper, C Taylor, C Nicolaisen, L Cochetti, E A Zoumi, D Annable, H Thomas, Z James-Knights, C Lee-Kim-Koon, E Akpinar, M J Coelho, E Darke, T Morris, F Mayer, J Forbes, S Gold, H Sinha, E Batchelor, L White, J Lund, S Forrest, H Dial, F Chishty, M Oliver, J Meyer, N Gokhare Viswanath, A Ammar, A Lopez, N Minhas, N Sunny, K Haynes, A Alexiadis, S Doski, R Arbuthnott, R Memon, K Bukhashem, A Chaudhary, A Sheik-Ali, A Sebastiao, C Taylor, E Clarke, S Misztal, D Raja, S K Chui, I Khalil, A Metsel, M Whelan, S Forrester, O Oboh, B Bayley-Skinner, R Eastwood, E Akapo, M Jamshaid, G Osborn, R Woods, O Wooler, S Chumley, E Cotton, N Jarrett, N Amanda, J Johnson, M Quhill, M Al-Ani, S Kandanearachchi, S Khan, A Barrow, M Dunlop, G Williams, S Phillips, F Faryad, A Hughes, S Wong Ching Hwai, C Johnston, R Guest, A Kaddouri, L Ernst, A Szasz, C Gafrey, G Loy, E Roberts, S Baker, A Davies, Z Khan, M Leonidou, J K Chow, R Murugan, A Baker, R Burns, Y C Foo, F Sikora, L Chong, C Chan Ah Song, C Cosgrove, O Reeve-Chen, J Ma, L Mccolm, M K Zhang, F Hussain, X Y Ng, B Ingabire, B Wagner, T Bain, R Kovacs, E Small, S Lam, L Yao, L Ho, H Paremes Sivam, K Dahal, A Stanley, M White, L Hayois, M Thillai, T Tay, E Davies, N Kamaruzaman, L Robinson, R Sherrington, Z Abid Sohail, S Lawrence, A Sohail, D Brown, S Parker, M Ohr, D Jasniak, B Crowther, F Jadoon, L J Melo, H Amin, Rqh Lim, M A Thaha, M Bath, S A Saadqain, Y Benallal, Z Jin, A Jackson, A Kuri, M Malik, H Jos, E T Goh, M E Paraskevopoulou, A Kythreotis, Y Naim Ahmed, S P Glynou, H Rehan, A Pereira Pai, A Georgiannakis, N Dworschak, S Cho, B J Chow, Y Khan, T Enthoven, A Malik, I Latif, A H Lakhani, N Rahman, K Morgan, S Gkolia, N Khalessi, Plz May, A Lwin, P Kanesaratnam, M Tudgee, R English, K Hu, N Pathanchaly, Dcs Chien, M Holloway, K Gao, G Lekka, R Srinivasan, S Zafar, M R Peris, K Keiarash, H Kanesan, B Chong, M Kobus, D Dominic, D A Do, D Dennis, S Frankland, V Shah, B Youssef, U Sadia, A Hassouneh, A Jaipersad, T Chen, M Fulford, A Dawson, K Kapur, T Jha, P Zope, M Wilkinson, V Bhatnagar, J Knight, T K Madhuri, T Katsiari, N Pasternak-Albert, E Moussa, L Emms, M Lamah, Z Batool, A Sabesan, S Islam, M Baig, M Dave, X Liu, L Stuart, S Harvey, V Dam, P Ezuma, J Lee, S Suntharalingam, S Singh, P Preston, F Rickard, T Gupta, S Madden, C Jones, R Gidwani, X Wang, M Mcconnell, S Hanna, A Mcgettigan, K Brown, A Cios, C Ward-Bradley, O Hurrell, V Paice, N Mutsonziwa, S Pogoson, R Coulter, J Corry, L Mcclean, F Keenan, S O’dolan, A Plonkowski, R Brady, R Ooi, D Iles, L S Guillemot, C Low, S El-Barraj, J Epstein, M Pressler, I Ioannou, N Soliman, A Ktayen, A Macconnachie, J Menendez Lorenzo, J Tooke, K Noman, L Wilkins, J J Warner-Levy, R Evans, A Torrance, C Fear, H Vidis Humphries, G Velayudham, Z Jefferson-Pillai, J Brady, R Oza, D Singh, A Hamid, P Hartop, P Shojaie, J Mano, T Rajah, Ymc Cabdi, A Singal, M Afzali, J M Lee, D Bandyopadhyay, J Bennett, P Mendenhall, A Millett, S Davidson, R Kallam, M Pishia, H Butler, S Isidore, D Boutsias, K Bryce, T De Rancourt, J Barry, H Henry, A Aspinall, L Smith, C Conway, H Shanmugathas, C Huang, M Doherty, K Ahmed, T Chung, T Benson, A Habiba, J Singh Bhangu, B Bingor, L Fice, H Premanandhan, J Mckenna, I Jimenez-Reid, M Hoque, E Griffiths, J Loughran, M Laskowski, A Mehmood, R O’kane, M Mullan, R Mcclenaghan, G Kettyle, F Hosty-Blaney, Xhf Chan, S Sunny, M Kaur, S Vose, V George, D Killoran, R Doherty, Z Zagorac, A Pullyblank, R Ismail, N Anderton, L Dwyer-Joyce, R Morgan, N Zhang, B French, H Cox, J Tetro, A Clarke, K Shaw, R Iau, E King, S Patel, M Ovakimian, A Zahid, G Rowley, R Tanner, K Davoudi, B Shear, M Xu, K Karan, A O’reilly, S Ahmed, T Iskrenova Kirilova, A Ezekiel, M Shapland, M Mitra, T Krum, G Higginbotham, R Winayak, J Mustow, L Yorke, C Gibb, A Ilyas, M Pound, A Abouharb, D Burke, A Khan, K Aimar, J Bennett, E Kerman-Fiore, S Rao, A Dayal, S Chawla, L Wadey, C Smith, K Desor, R Surti, Z Gul, V Gourgiotis, L M Kaselampao, C Nguik, R Agrawal, Q Y Liaw, E Mckeown, A Hassan, K Krishnan, H O Glover, M Devassy, D Ogunyanwo, G Dickenson, G Elder, G Valdez, B White, I Hamilton, S Shah, S Sinha, K Punwani, I Peat, M Hayat, N Khalid, E Frankel, H Macgowan, A Braka, A Byfield, J Brooks, A Kashif, R Archer, S Akkaya, M Sood, S Akther, O Birkett, T Kadri, S Abdulmula, P Psefteli, S Chadha, G Reese, C Mark, M Asunramu, T Hess, S Mehta, C Tsang, K Syed, A Alocious, M Steinruecke, M Ashley, R Newton, J Wainwright, J Ayathamattam, H Wong, Z Elahi, M Fleming, T Ali, D Lloyd, K Brooks, C Kwon, A Mckerrell, T Jamadar, M D Barcelona, D Polluk, J Kwon, F Henry, S Ayirookuzhi, M Mwipatayi, M Cheruiyot, S Adam, T Cruickshank, K Gollub, M Revell, S Taiwo, A Atchade, A Harewood, A Niina, D Bosanquet, O Mckeon-Williams, T Szakmany, E Badhams, N Christensen, J Sammut, N Tay, J M Pollok, D Raptis, M Varcada, S Ganesananthan, R Chandrasekar, A Deshpande, L P Ghoora, G Hogg, S Staubli, M Banerjee, S Sunil Menon, A Sanz Pena, R Rana, A Mohamed, P Crabtree, A Flower, A Banerjee, Y Talabi, N Bishop, N Kupfuwa, D Ilangovan, H Qureshi, I Minty, R Baron, L Greasley, P Birkenhead, R Baron, M Shahid, N Rajasivam, W L Chu, N Punnoose, V Sundaresan, M El-Galley, K Prasansapakit, S Mitra, M Garlick, M Prakash, H Gao, C Parmar, F Lee, A Aich, A Ismail, M Kirupaharan, S Khan, K Patel, D Mclaverty, G Hammerton, H Walford, H Roberts, P Cautivo, H Abdel Kader, A Kimble, K Manaf, R Mackonochie, V Mitchell, S Ghaznavi, A Taheem, E Firth, H Chandler, K Eldessouki, J Fyfe, M Bhat, J Cavlin, E Pearce, S Patterson, C Johnson, K El-Badawi, M Gariballa, E Hale, H Younis, E Brackenbury, A Chappell, T Poulton, E Dawson, A Murray, I Mcallister, A Hassan, B Cunningham, T Chan-A-Sue, N Rajpal, P Keane, K Walsh, L Mcgeoghan, M Neeson, H Brown, S M Adams, S Ladha, A Walsh, M Alradhawi, R Tarighi, M I Miah, K Dawas, A Mojadady, A Priestman-Degano, S K Vellore Sasikumar, B Suresh, M Fornasiero, D Csvila, A Kumar, S T Adil, H S Adil, U Kataria, R Jaibaji, N Patel, A Goch, P Quaye, A Gupte, H Mustafa, V Otti, D Dewantoro, M Ali, M Sood, H Wright, L Abusheba, L Wong, M Docksey, A Abdinasir, M O Karim, V Kolaityte, M Vasileiadou Pelling, A Abdel Basit, E Pearson, E Hughes, A S Millington, L Taylor, C Borg, K Jagic, A Waheed, K Singh, V James, T Chowdhury, A Saxena, A Georgiou, T Jones, C Carpenter, I Hughes, Z Aloul, C Hanna-Davies, A Jacob, A Puthiyakunnel Saji, A Qureshi, Z Ulfin, M Dunlop, R Woods, N Hill, N Mohamed, A Vora, H Asharaf, E Maye, U Arif, S Srinivasan, S Prasad, B John, V Shivanand, A Singh, A Zahid, E Stewart, M Raketla, O Kokoricha, D Karwa, J Crisp, R Shehadeh, J Lee, B Webb, S Lepping, F Yusuf, E Pak, B Soo, P Pemmasani, S Goyal, A B Binti Azad Bashir, S Komolafe, A Naeem, H R Anbananthan, L K Au Yong, S Mookerjee, N Ward, M Aniq, J Davis, A Al-Sukaini, J Anthony, J Boyle, A Laird, D Speake, C Beagan, S Stewart, O El-Koubani, R Chan, M Viswanath, S Janssens, I Shah, V Nguyen, E Mckee, C W Ng, M Balać, D I Suresh Kumar, S Seeva Balan, R Kirk, B Miles, L A Kovacic, V G Collins, J Low, W Sim, S J Chua, N Narayanan, T Y Lee, Cmn Lo, M Chauhan, J Elkafsi, S Rasul, R Yammine, Z Sattar, D Dixit, N Rahman, J Davies, S Dindyal, L Osborne, L Nip, N Wilding de Miranda, A Stevens, B Al-Diri, A Lim, G Kallikas, Y Kim, M Anjum, P Jeyapahan, M Hakim, S Singh, E Jamileh, C Sohrabi, S H Baek, N Sadik, A Mohammed, D Aje, R Clifford, C O’halloran, K Mahajan, N Darke, S Lloyd, M Mlotshwa, K Melhuish, J Derex-Briggs, M Bell, P Varma, K Fray, Y Garg, K Datta, T Mantel-Cooper, M Goodfellow, B Kazi, N Sievers, K Telford, Z R Almansoor, K Craddock, J L Tan, L St John, A Singhania, S Dosani, S Mughal, N Bokhari, L Brooks, I Laid, A Lala, C S Ong, S Wakefield, S Phillips, H Unwin

## Abstract

**Background:**

There is low-certainty evidence on the impact of extended pharmacological prophylaxis on venous thromboembolism-associated morbidity and mortality. The aim of this study was to determine the efficacy and safety of extended prophylaxis after major abdominopelvic surgery for the prevention of clinically relevant venous thromboembolism after hospital discharge.

**Methods:**

CArdiovaSCulAr outcomes after major abDominal surgEry (CASCADE) was a prospective, international, cohort study into which consecutive adult patients undergoing major abdominopelvic surgery were enrolled (January–May 2022). Extended prophylaxis was considered at least 28 days of anticoagulant prescription after surgery. The primary efficacy outcome was clinically relevant venous thromboembolism and the primary safety outcome was clinically relevant bleeding within 30 days after surgery (European Medicines Agency definitions). The independent association of these outcomes with extended prophylaxis was explored using mixed-effects logistic regression and propensity score weighting.

**Results:**

A total of 11 571 patients (median age of 58.0 years; 6399 (55.3%) women) from 29 countries were included. The extended prophylaxis prescription rate was 31.7% (3670 patients). The post-discharge venous thromboembolism and bleeding rates were 0.1% (12 patients) and 0.7% (85 patients) respectively. After weighting, extended prophylaxis was not significantly associated with increased bleeding risk (OR 1.07 (95% c.i. 0.64 to 1.81); *P* = 0.792) or decreased venous thromboembolism incidence, both in the overall cohort (OR 1.13 (95% c.i. 0.33 to 3.90); *P* = 0.848) and in a subgroup analysis of patients undergoing complex major surgery and with active cancer (OR: 1.36 (95% c.i. 0.33 to 5.57); *P* = 0.669).

**Conclusion:**

In modern practice, the incidence of postoperative venous thromboembolism is low. Extended prophylaxis appears safe, yet the clinical efficacy remains uncertain. Further work is required to define patients who stand to benefit.

## Teaser

Despite low-certainty evidence on the impact of extended pharmacological prophylaxis on venous thromboembolism-associated morbidity and mortality, current guidelines recommend a minimum of 28 days of postoperative pharmacological prophylaxis. The findings suggest that extended prophylaxis may not be beneficial for all patients undergoing major abdominal and pelvic surgery either for benign or malignant indications. A tailored approach that uses patient- and procedure-specific venous thromboembolism profiles should be adopted to identify those patients that stand to benefit.

## Introduction

Major abdominal and pelvic surgery is a well-established risk factor for the development of venous thromboembolism (VTE), which is associated with significant morbidity and mortality^1,2^. Current evidence suggests that VTE risk is not limited to the immediate postoperative interval and, for some patients, it can remain significant even 3–6 months after surgery^3,4^. To minimize this risk, current guidelines recommend a minimum of 28 days of postoperative pharmacological prophylaxis with low molecular weight heparin (LMWH), also known as extended pharmacological prophylaxis, for patients undergoing major abdominal and pelvic surgery, with some guidelines limiting this recommendation to cancer surgeries only^5–7^.

However, this recommendation is based on low-certainty evidence. The majority of the studies supporting the use of extended pharmacological prophylaxis were conducted before the introduction of Enhanced Recovery After Surgery (ERAS) protocols and the widespread adoption of minimally invasive surgery, resulting in study populations that are not comparable to contemporary surgical practices^8–10^. In addition, most RCTs investigating the use of extended prophylaxis adopted outcomes with limited clinical relevance and required the use of invasive diagnostic techniques, such as venography, that are not part of standard practice, all of which have implications as to whether their conclusions on the impact of extended prophylaxis on VTE-associated morbidity and mortality are clinically relevant^8–10^. The current validity of the guidelines is also questioned by surgeons, as evidenced by their limited compliance with the policy, due to concerns about LWMH costs, suboptimal patient adherence to daily subcutaneous injections, and the potential negative impact the widespread adoption of the policy might have on the finances and resources of healthcare systems^11–14^.

The aim of this analysis of the CArdiovaSCulAr outcomes after major abDominal surgEry (CASCADE) study was to determine the efficacy and safety of extended prophylaxis after major abdominal and pelvic surgery for the prevention of post-discharge clinically relevant VTE in a contemporary, international surgical population using pragmatic, clinically relevant outcomes.

## Methods

### Study design

CASCADE was an international, observational, prospective cohort study, which was conducted according to a published protocol^15^. This analysis was performed according to STROBE reporting guidelines for observational studies^16^.

CASCADE was delivered by the STARSurg and EuroSurg student- and trainee-led collaborative groups. Any centre conducting major abdominopelvic surgery in Europe was invited to participate, with prospective identification of patients by local collaborators across a predefined, 2-week data collection window between 23 January and 1 May 2022. Routine, anonymized data were collected with no change to clinical care pathways. Before data collection, confirmation of appropriate local and/or national regulatory approval, according to country-specific regulations, was required.

The protocol for the CASCADE project was pre-published; however, this study was not pre-registered in an institutional registry.

### Eligibility criteria

Consecutive adult patients (greater than or equal to 18 years of age) undergoing major abdominal and pelvic surgical procedures through any operative approach were eligible (abdominal and/or pelvic visceral resection; formation or reversal of stoma; or anterior abdominal wall hernia repair). Planned day-case procedures and those performed for traumatic indications or without visceral resection were not studied.

Specific to this pre-planned analysis of the CASCADE dataset, recruited eligible patients were excluded from the statistical analysis based on the following criteria: prescription for long-term therapeutic anticoagulation before admission; inpatient mortality or duration of hospital stay greater than or equal to 14 days; prescription for therapeutic anticoagulation on discharge; and inpatient clinically relevant bleeding event. These criteria were adopted to exclude from the analysis patients with contraindications to extended VTE prophylaxis or insufficient post-discharge time to observe the outcomes of interest. In addition, patients who were prescribed prophylactic LMWH beyond discharge, but for less than 28 days after surgery, were excluded from the analysis as they did not receive extended prophylaxis, according to the study’s definition of extended prophylaxis.

### Outcome measures

All patients were followed up for 30 days after surgery. The primary efficacy outcome was the rate of post-discharge VTE events. This was defined as a composite endpoint consisting of clinically relevant and radiologically diagnosed events: proximal deep-vein thrombosis (DVT; asymptomatic and symptomatic); symptomatic distal DVT; symptomatic non-fatal pulmonary embolism (PE); and VTE-related deaths (European Medicines Agency definition^17^). Given that this study did not modify clinical practice, routine imaging of all patients at predefined time points was not performed. Imaging was performed only if deemed necessary by the local teams based on clinical suspicion. The choice of the most appropriate imaging modality was left to the local teams. The primary safety outcome was the rate of post-discharge clinically relevant bleeding events, a composite endpoint of major bleeding and clinically relevant non-major bleeding, and defined as any bleeding that required medical attention and/or had clinical consequences for a patient (European Medicines Agency definition^17^).

### Explanatory variables

The main explanatory variable of interest was the prescription of extended pharmacological VTE prophylaxis. In this analysis, this was defined as at least 28 days of LMWH prescription after surgery, whereas conventional prophylaxis was defined as LMWH prescription until discharge. Given that this study did not modify clinical practice, the choice of the most appropriate prophylactic LMWH dosing (that is fixed dose or anti-factor Xa-based dose) was left to the local teams. Similarly, the most appropriate mechanical thromboprophylaxis devices were used by the local teams where clinically indicated.

Additional variables were collected to risk-adjust outcomes for the following potential confounding factors: age; sex; smoking status; BMI; ASA grade; respiratory, cardiovascular, inflammatory, and metabolic diseases (chronic obstructive pulmonary disease, ischaemic heart disease, cerebrovascular disease, inflammatory bowel disease, and diabetes mellitus); liver cirrhosis; renal disease (chronic kidney disease stage IIIB–V); previous VTE events; active cancer (defined as surgery for malignant indications and/or known malignancy at the time of surgery); operative approach (open or minimally invasive); operative urgency (elective or emergency); operative contamination (clean-contaminated, contaminated, or dirty); duration of operation; critical care (ICU/high dependency unit) admission; postoperative SARS-CoV-2 infection; and duration of hospital stay. Surgical procedures were stratified according to the anatomical region involved (abdominal or pelvic) and complexity (complex major or major). Complex major procedures were defined as procedures coded as major plus or complex major by the Bupa Schedule of Procedures^18^.

### Statistical analyses

Patient demographics, perioperative variables, and outcomes were compared for extended and conventional pharmacological VTE prophylaxis groups. Categorical variables are cross-tabulated and were compared using the chi-squared or Fisher’s exact tests. Continuous variables are summarized as median (interquartile range (i.q.r.)) and were compared using the Mann–Whitney test. Funnel plots with 95% and 99% confidence intervals are used to present extended VTE prophylaxis prescription rates and VTE rates (unadjusted and risk-adjusted) of participating centres. Adjustment was performed using mixed-effects logistic regression models. Clinically plausible perioperative factors associated with postoperative VTE, extended VTE prophylaxis prescription, and clinical outcomes were incorporated into the modelling approach as fixed effects and hospital was used as a random effect.

Mixed-effects multivariable logistic regression was performed to determine whether extended pharmacological VTE prophylaxis was independently associated with the occurrence of post-discharge clinically relevant VTE and bleeding events. The same modelling approach was adopted that was used to derive risk-adjusted extended VTE prophylaxis prescription rates. To minimize the risk of model overfitting, reduced models with limited numbers of explanatory variables were produced. Final model selection was guided by expert opinion; variables included in the reduced models were patients’ demographics and perioperative factors most clinically relevant to the outcomes of interest.

To investigate the association between extended prophylaxis and clinical outcomes, propensity score weighting (inverse probability of treatment weighting) was used to address confounding in those who did or did not receive extended prophylaxis. The propensity score was defined using multivariable logistic regression as the probability that a patient would be prescribed extended pharmacological VTE prophylaxis based on the following covariates: age, sex, BMI, ASA grade, previous VTE events, active cancer, abdominal or pelvic surgery, operative approach, operative urgency, and critical care admission. Due to group sizes, the hospital-level identifier (random effect) was not used in the weighting procedure. The balance in the perioperative factors between groups was assessed before and after using the absolute standardized mean difference and a value below 0.1 was considered to indicate that a variable was well balanced between groups. The weighted data set was then used to fit outcome models using logistic regression, based on the same variables used to generate the propensity score weights. G-computation was used to estimate the treatment effect.

For post-discharge clinically relevant VTE, two a priori subgroup analyses were performed to further limit the population heterogeneity and examine more closely the subgroups of patients that tend to be prescribed extended prophylaxis in clinical practice due to their higher VTE risk. The first included patients undergoing complex major surgery and the second included patients undergoing complex major surgery and with active cancer at the time of surgery.

All effect estimates are presented as OR (95% c.i.). The threshold for statistical significance was set a priori as *P* < 0.050. All analyses were performed using R version 4.1.1 (R Foundation for Statistical Computing, Vienna, Austria) with the tidyverse, finalfit, weightIt, and cobalt packages.

## Results

### Overall venous thromboembolism events

**Fig. 1:**
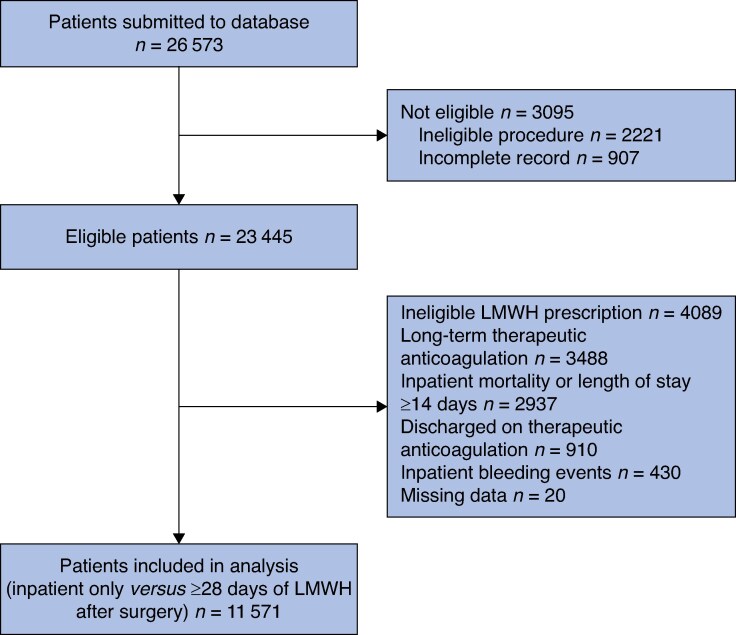
Flow diagram of patient inclusion and exclusion LMWH, low molecular weight heparin.

In total, 23 445 eligible patients were recruited from 445 hospitals across 29 European countries (median age of 61.0 years; 12 497 (53.3%) women) (*Fig. 1* and *Tables S1*, *S2*). The postoperative rate of clinically relevant VTE events was 0.7% (172 patients), with most patients diagnosed before discharge (81.4%, 140 patients) and a few patients diagnosed after discharge (18.6%, 32 patients) (*Fig. S1*). After adjusting for confounding variables using mixed-effects logistic regression, the median postoperative VTE rate per centre was 0.2% (i.q.r. 0.1%–0.5%) (*Fig. S2*). A full breakdown of baseline characteristics of the overall cohort stratified by VTE event and of operations performed is provided in *Tables S1*, *S3*.

### Extended pharmacological venous thromboembolism prophylaxis

Overall, 11 571 patients were included in the analysis (*Fig. 1*). Some 3670 patients (31.7%) were prescribed extended pharmacological VTE prophylaxis (greater than or equal to 28 days of LWMH after surgery) for a median duration of 32.0 (i.q.r. 29.0–36.0) days. Conventional prophylaxis (inpatient-only LWMH; 68.3%, 7901 patients) was given for a median duration of 3.0 (i.q.r. 1.0–5.0) days. See *Table 1*. The five most common operations were cholecystectomy (20.2%), appendicectomy (12.4%), hysterectomy (9.5%), right hemicolectomy (7.0%), and excision of the rectum (abdominoperineal resection, anterior resection, or Hartmann’s procedure; 6.6%). A full breakdown of operations included in the analysis is provided in *Table S4*.

**Table 1:** Perioperative variables stratified by extended pharmacological venous thromboembolism prophylaxis

	Extended pharmacological VTE prophylaxis (≥28 days of LWMH)			
	No (*n* = 7901)	Yes (*n* = 3670)	Total (*n* = 11 571)	P*
**Postoperative VTE prophylaxis (days)**				
Median (i.q.r.)	3.0 (1.0–5.0)	32.0 (29.0–36.0)	5.0 (2.0–29.0)	<0.001†
**Age (years)**				
Median (i.q.r.)	53.0 (39.0–65.0)	64.0 (55.0–73.0)	58.0 (44.0–69.0)	<0.001†
**Sex**				
Male	3393 (42.9)	1773 (48.3)	5166 (44.6)	<0.001
Female	4505 (57.0)	1894 (51.6)	6399 (55.3)	
Missing	3 (0.0)	3 (0.1)	6 (0.1)	
**BMI**				
Normal	2571 (32.5)	1200 (32.7)	3771 (32.6)	0.044
Underweight	136 (1.7)	82 (2.2)	218 (1.9)	
Overweight	2500 (31.6)	1274 (34.7)	3774 (32.6)	
Obese	1662 (21.0)	785 (21.4)	2447 (21.1)	
Morbidly obese	211 (2.7)	128 (3.5)	339 (2.9)	
Missing	821 (10.4)	201 (5.5)	1022 (8.8)	
**Smoking status**				
Never	3931 (49.8)	1863 (50.8)	5794 (50.1)	<0.001
Ex-smoker	1110 (14.0)	791 (21.6)	1901 (16.4)	
Current‡	1642 (20.8)	563 (15.3)	2205 (19.1)	
Missing	1218 (15.4)	453 (12.3)	1671 (14.4)	
**ASA grade**				
I–II	6239 (79.0)	2437 (66.4)	8676 (75.0)	<0.001
III–V	1437 (18.2)	1119 (30.5)	2556 (22.1)	
Missing	225 (2.8)	114 (3.1)	339 (2.9)	
**Diabetes mellitus**				
No	6978 (88.3)	3087 (84.1)	10 065 (87.0)	<0.001
Non-IDDM	741 (9.4)	467 (12.7)	1208 (10.4)	
IDDM	173 (2.2)	113 (3.1)	286 (2.5)	
Missing	9 (0.1)	3 (0.1)	12 (0.1)	
**Previous VTE**				
No	7808 (98.8)	3603 (98.2)	11 411 (98.6)	0.009
Yes	84 (1.1)	61 (1.7)	145 (1.3)	
Missing	9 (0.1)	6 (0.2)	15 (0.1)	
**Active cancer**				
No	5981 (75.7)	632 (17.2)	6613 (57.2)	<0.001
Yes	1902 (24.1)	3032 (82.6)	4934 (42.6)	
Missing	18 (0.2)	6 (0.2)	24 (0.2)	
**IBD**				
No	7605 (96.3)	3511 (95.7)	11 116 (96.1)	0.127
Yes	288 (3.6)	156 (4.3)	444 (3.8)	
Missing	8 (0.1)	3 (0.1)	11 (0.1)	
**COPD**				
No	7629 (96.6)	3466 (94.4)	11 095 (95.9)	<0.001
Yes	265 (3.4)	199 (5.4)	464 (4.0)	
Missing	7 (0.1)	5 (0.1)	12 (0.1)	
**Type of surgery**				
Abdominal	6149 (77.8)	1999 (54.5)	8148 (70.4)	<0.001
Pelvic	1741 (22.0)	1666 (45.4)	3407 (29.4)	
Missing	11 (0.1)	5 (0.1)	16 (0.1)	
**Operative complexity**				
Major	4610 (58.3)	329 (9.0)	4939 (42.7)	<0.001
Complex major	3291 (41.7)	3341 (91.0)	6632 (57.3)	
**Operative urgency**				
Elective	5142 (65.1)	3106 (84.6)	8248 (71.3)	<0.001
Emergency	2749 (34.8)	557 (15.2)	3306 (28.6)	
Missing	10 (0.1)	7 (0.2)	17 (0.1)	
**Operative contamination**				
Clean-contaminated	7340 (92.9)	3487 (95.0)	10 827 (93.6)	<0.001
Contaminated/dirty	543 (6.9)	166 (4.5)	709 (6.1)	
Missing	18 (0.2)	17 (0.5)	35 (0.3)	
**Operative approach**				
Open	2740 (34.7)	1691 (46.1)	4431 (38.3)	<0.001
Minimally invasive	5150 (65.2)	1976 (53.8)	7126 (61.6)	
Missing	11 (0.1)	3 (0.1)	14 (0.1)	
**Duration of operation (min)**				
Median (i.q.r.)	95.0 (60.0–150.0)	183.0 (135.0–255.0)	120.0 (75.0–190.0)	<0.001†
**Critical care admission**				
No	6831 (86.5)	2794 (76.1)	9625 (83.2)	<0.001
Yes	1069 (13.5)	875 (23.8)	1944 (16.8)	
Missing	1 (0.0)	1 (0.0)	2 (0.0)	
**Postoperative SARS-CoV-2 infection**				
No	7569 (95.8)	3538 (96.4)	11 107 (96.0)	0.128
Yes	312 (3.9)	123 (3.4)	435 (3.8)	
Missing	20 (0.3)	9 (0.2)	29 (0.3)	
**Duration of hospital stay (days)**				
Median (i.q.r.)	3.0 (1.0–5.0)	6.0 (4.0–8.0)	4.0 (2.0–7.0)	<0.001†

Values are *n* (%) unless otherwise indicated. *Chi-squared test or Fisher’s exact test unless otherwise indicated. †Mann–Whitney test. ‡Includes those who stopped smoking within 6 weeks of surgery. VTE, venous thromboembolism; LMWH, low molecular weight heparin; i.q.r., interquartile range; IDDM, insulin-dependent diabetes mellitus; IBD, inflammatory bowel disease; COPD, chronic obstructive pulmonary disease.

Compared with patients prescribed conventional prophylaxis, patients receiving extended prophylaxis were older (median age of 64.0 *versus* 53.0 years; *P* < 0.001), had a poorer physical status (ASA grade III–V: 30.5% *versus* 18.2%; *P* < 0.001), and had a greater prevalence of active cancer at the time of surgery (82.6% *versus* 24.1%; *P* < 0.001) (*Table 1*). Surgery for patients who received extended prophylaxis more frequently involved the pelvic region (45.4% *versus* 22.0%; *P* < 0.001), was more complex (complex major procedures: 91.0% *versus* 41.7%; *P* < 0.001), had a longer duration (median of 183.0 *versus* 95.0 min; *P* < 0.001), required critical care admission (23.8% *versus* 13.5%; *P* < 0.001), and required a longer hospital stay (median of 6.0 *versus* 3.0 days; *P* < 0.001) (*Table 1*). In contrast, surgery for patients who received conventional prophylaxis was more frequently performed in emergency settings (34.8% *versus* 15.2%; *P* < 0.001) and with a minimally invasive approach (65.2% *versus* 53.8%; *P* < 0.001) (*Table 1*).

**Fig. 2:**
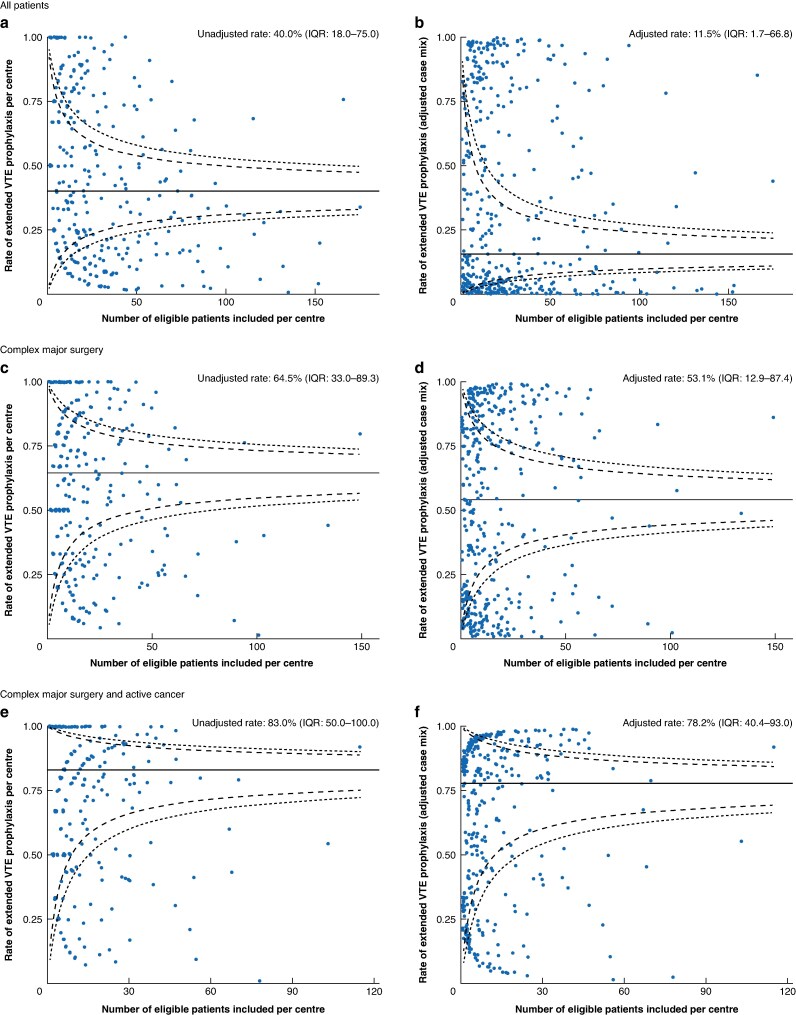
Funnel plots for rate of extended venous thromboembolism prophylaxis per centre (overall rate and adjusted for case mix) Circles, continuous lines, dashed lines, and dotted lines represent single centres, overall medians, 95% confidence intervals, and 99% confidence intervals respectively. a, c, e shows the rate of extended VTE prophylaxis per centre. b, d, f shows the adjusted rate. The extended venous thromboembolism prophylaxis rate was adjusted for: age, sex, BMI, ASA grade, previous VTE events, active cancer, type of procedure, operative urgency, operative approach, duration of operation, critical care admission, and hospital (random effect). VTE, venous thromboembolism.

Among all 445 centres, the median extended VTE prophylaxis prescription rate was 40.0% (i.q.r. 18.0%–75.0%) (*Fig. 2a*). This substantial variation in practice could not be explained based on case mix after adjustment using mixed-effects logistic regression (median of 11.5% (i.q.r. 1.7%–66.8%)) (*Fig. 2b*).

#### Complex major surgery subgroup analysis

When limiting the analysis to patients undergoing complex major surgery (6632 patients), extended VTE prophylaxis was prescribed for 3341 patients (50.4%) (*Table S7*). The five most common operations were hysterectomy (16.6%), right hemicolectomy (12.2%), excision of the rectum (11.5%), nephrectomy (7.0%), and prostatectomy (6.0%) (*Table S5*).

Similarly to the overall analysis, extended prophylaxis was prescribed for patients who were older, with poorer physical status and active cancer, and who underwent longer procedures that more frequently involved the pelvic region. Patients undergoing emergency surgery more frequently received prophylactic LMWH only whilst inpatients (20.0% *versus* 13.6%; *P* < 0.001) (*Table S7*).

A substantial variation amongst participating centres in the prescription rate of extended VTE prophylaxis was also observed in this subgroup analysis, even after adjusting for case mix (median extended VTE prophylaxis prescription rate of 53.1% (i.q.r. 12.9%–87.4%)) (*Fig. 2d*).

#### Complex major surgery and active cancer subgroup analysis

The second subgroup analysis only included patients undergoing complex major surgery and with active cancer (4427 patients) and 64.5% (2857 patients) received extended VTE prophylaxis (*Table S8*). The five most common operations were right hemicolectomy (15.3%), excision of the rectum (15.3%), hysterectomy (12.7%), nephrectomy (8.2%), and prostatectomy (7.9%) (*Table S6*).

Different from the previous analyses, in this subgroup analysis, patients in the extended and conventional prophylaxis groups were equally older and with more co-morbidities. Extended prophylaxis remained more frequently prescribed after surgery that involved the pelvic region. Approximately half of included procedures were performed with a minimally invasive approach (*Table S8*).

Despite a greater overall extended prophylaxis prescription rate, a substantial variation amongst participating centres was observed in this subgroup analysis as well (adjusted median extended VTE prophylaxis prescription rate of 78.2% (i.q.r. 40.4%–93.0%)) (*Fig. 2f*).

Propensity score weighting produced well-balanced treatment groups in the subgroup analyses. All variables bar two were well balanced in the overall analysis—operative approach and duration of operation, which were only marginally unbalanced. See *Tables S13–S15* and *Fig. S3*.

### Post-discharge clinically relevant venous thromboembolism

In the overall analysis, 12 patients (0.1%) were diagnosed with post-discharge clinically relevant VTE (5 patients with symptomatic PE, 1 patient with symptomatic proximal DVT, 2 patients with asymptomatic proximal DVT, and 4 patients with symptomatic distal DVT) (*Table 2*). No VTE-related deaths were observed. After adjusting for confounding factors using mixed-effects models and propensity score weighting, no significant association between extended VTE prophylaxis and the incidence of post-discharge VTE was shown in the overall and subgroup analyses (*Table 3* and *Tables S9–S11*).

**Table 2:** Post-discharge venous thromboembolism and bleeding events at 30 days, by extended pharmacological venous thromboembolism prophylaxis, before and after propensity score weighting

	Extended pharmacological VTE prophylaxis (≥28 days of LWMH)					
	Unweighted				PS weighted	
	No	Yes	Total	P*	No, %	Yes, %
**All patients**	*n* = 7901	*n* = 3670	*n* = 11 571			
Post-discharge clinically relevant VTE events (overall)						
No	7896 (99.9)	3663 (99.8)	11 559 (99.9)	0.062	99.9	99.9
Yes	5 (0.1)	7 (0.2)	12 (0.1)		0.1	0.1
Post-discharge clinically relevant VTE events (by type of event)						
No	7896 (99.9)	3663 (99.8)	11 559 (99.9)	0.041		
DVT	4 (0.1)	3 (0.1)	7 (0.1)			
PE	1 (0.0)	4 (0.1)	5 (0.0)			
Post-discharge clinically relevant bleeding events (overall)						
No	7850 (99.4)	3634 (99.0)	11 484 (99.2)	0.046	99.3	99.2
Yes	49 (0.6)	36 (1.0)	85 (0.7)		0.7	0.8
Missing	2 (0.0)	0 (0.0)	2 (0.0)			
Post-discharge clinically relevant bleeding events (by type of event)						
No	7850 (99.4)	3634 (99.0)	11 484 (99.2)	0.098		
Clinically relevant non-major bleeding	39 (0.5)	29 (0.8)	68 (0.6)			
Major bleeding	10 (0.1)	7 (0.2)	17 (0.1)			
Missing	2 (0.0)	0 (0.0)	2 (0.0)			
**Complex major surgery**	*n* = 3291	*n* = 3341	*n* = 6632			
Post-discharge clinically relevant VTE events (overall)						
No	3287 (99.9)	3334 (99.8)	6621 (99.8)	0.549	99.8	99.8
Yes	4 (0.1)	7 (0.2)	11 (0.2)		0.2	0.2
Post-discharge clinically relevant VTE events (by type of event)						
No	3287 (99.9)	3334 (99.8)	6621 (99.8)	0.512		
DVT	3 (0.1)	3 (0.1)	6 (0.1)			
PE	1 (0.0)	4 (0.1)	5 (0.1)			
**Complex major surgery and active cancer**	*n* = 1570	*n* = 2857	*n* = 4427			
Post-discharge clinically relevant VTE events (overall)						
No	1567 (99.8)	2851 (99.8)	4418 (99.8)	1.000	99.8	99.7
Yes	3 (0.2)	6 (0.2)	9 (0.2)		0.2	0.3
Post-discharge clinically relevant VTE events (by type of event)						
No	1567 (99.8)	2851 (99.8)	4418 (99.8)	0.157		
DVT	3 (0.2)	2 (0.1)	5 (0.1)			
PE	0 (0.0)	4 (0.1)	4 (0.1)			

Values are *n* (%) unless otherwise indicated. *Chi-squared test or Fisher’s exact test. VTE, venous thromboembolism; LMWH, low molecular weight heparin; PS, propensity score; DVT, deep-vein thrombosis; PE, pulmonary embolism.

**Table 3:** Summary of effect estimates of extended pharmacological prophylaxis (greater than or equal to 28 days of low molecular weight heparin) for 30-day post-discharge clinically relevant venous thromboembolism and bleeding events, before and after propensity score weighting

	Univariable analysis		Multilevel analysis		PS weighting analysis	
	OR (95% c.i.); *P*	Number in model	OR (95% c.i.); *P*	Number in model	OR (95% c.i.); *P*	Number in model
**Post-discharge clinically relevant VTE**						
All patients	3.02 (0.96,10.20); 0.059	11 571	1.61 (0.44,5.90); 0.473	11 545	1.13 (0.33,3.90); 0.848	9889
Complex major surgery	1.73 (0.52,6.59); 0.385	6632	1.40 (0.38,5.19); 0.613	6626	1.15 (0.32,4.21); 0.828	5769
Complex major surgery and active cancer	1.10 (0.29,5.21); 0.894	4427	1.14 (0.28,4.63); 0.851	4426	1.36 (0.33,5.57); 0.669	3907
**Post-discharge clinically relevant bleeding**						
All patients	1.59 (1.02,2.44); 0.036	11 569	1.34 (0.80,2.24); 0.265	10 536	1.07 (0.64,1.81); 0.792	9887

The reference group is pharmacological venous thromboembolism prophylaxis until discharge. PS, propensity score; VTE, venous thromboembolism.

### Post-discharge clinically relevant bleeding

Some 85 (0.7%) post-discharge clinically relevant bleeding events were observed (68 clinically relevant non-major bleeding events and 17 major bleeding events) (*Table 2*). After risk adjustment and accounting for treatment selection bias with propensity score weighting, no significant association between extended VTE prophylaxis and the incidence of post-discharge bleeding was shown (*Table 3* and *Table S12*).

## Discussion

Current guidelines recommend extended pharmacological prophylaxis for patients undergoing major abdominal and pelvic surgery to minimize the incidence of postoperative VTE^5–7^. However, this recommendation is based on low-certainty evidence. This international, prospective observational study with pragmatic, clinically relevant outcomes fails to demonstrate a significant association between extended pharmacological prophylaxis and decreased post-discharge VTE incidence.

In this heterogeneous cohort, the incidence of clinically relevant postoperative VTE within 30 days of surgery was low. The vast majority of recorded VTE events occurred before discharge, highlighting the importance of adequate inpatient mechanical and pharmacological prophylaxis, as well as early mobilization and adherence to enhanced recovery where possible^19^. The findings are comparable to those of a recent meta-analysis, which reported that almost 50% of postoperative symptomatic VTE events occurred within 7 days of surgery^20^.

Across participating centres, a substantial variation in extended pharmacological VTE prophylaxis prescription practices was observed, even after case-mix adjustment. Poor surgeon compliance with VTE prevention guidelines has been well documented in the literature^14,21,22^. The reluctance to prescribe extended LMWH courses has been attributed to the low-certainty evidence that the recommendations are based on, LMWH costs, and concerns regarding patient adherence to daily subcutaneous LMWH injections^23–25^. In the present study it was also observed that extended prophylaxis was less frequently prescribed after emergency surgery. Given that there is evidence to support emergency procedures having equal or greater postoperative VTE risk compared with their elective counterparts^26^, this finding, in combination with the abovementioned poor compliance with the VTE prevention guidelines, questions the current understanding of the surgical community regarding which patients would benefit from extended pharmacological VTE prophylaxis after major abdominal and pelvic surgery.

The primary efficacy outcome of the present study was the rate of post-discharge clinically relevant VTE. This analysis did not show a significant association between extended pharmacological prophylaxis and VTE incidence. Although most RCTs have shown a reduction in postoperative VTE events in patients receiving extended prophylaxis^8–10,27^, recent cohort studies and RCTs with clinically relevant outcomes have failed to replicate these results^22,28–30^, questioning whether the real-world effectiveness of extended VTE prophylaxis may differ compared with the findings of some RCTs.

Extended pharmacological VTE prophylaxis has the potential to increase postoperative bleeding risk. In the present cohort, after multivariable adjustment, there was no significant difference between patients who did and did not receive extended prophylaxis with regard to post-discharge clinically relevant bleeding events. This is consistent with the literature and reinforces the notion that extended prophylaxis is safe in patients with no contraindications^22,30–32^.

There are limitations to this study. As a result of its observational nature, limited conclusions can be drawn from the findings. Multivariable analyses and propensity score weighting were used to adjust for potentially confounding factors, but the statistical power of the analyses was limited by the relatively low number of events despite the large sample size. Another potential weakness is that VTE incidence might have been underestimated; the follow-up was limited to 30 rather than 90 days and data were collected from hospital electronic records and clinical notes, with the implication that VTE events diagnosed at different hospitals or in primary care might have been missed. However, this is unlikely to have affected the analysis in a significant way, as patients treated with extended VTE prophylaxis and those receiving conventional prophylaxis were equally affected by this limitation. Also, the present study did not capture patient adherence to extended LMWH prescription. It is plausible that not all patients who received a prescription for extended VTE prophylaxis were fully compliant with the treatment. In addition, as this study did not modify routine clinical practice, the choices of the most appropriate diagnostic imaging modality for VTE, prophylactic LMWH dosing, and mechanical thromboprophylaxis devices were left to the local teams, which could have introduced potential bias in the study.

The present study, which was conducted using pragmatic, clinically relevant outcomes in a contemporary, international surgical cohort, suggests that extended prophylaxis may not be beneficial for all patients undergoing major abdominal and pelvic surgery either for benign or malignant indications. Current clinical guidelines have questionable validity, given that they are based on old studies that do not reflect current surgical practices^8–10^. The introduction of minimally invasive techniques and the introduction of enhanced recovery protocols have significantly reduced the incidence of postoperative VTE to the point that the rigid use of extended prophylaxis, as dictated by current guidelines, is not cost-effective^33,34^. Intuitively, across all patients undergoing major abdominal and pelvic surgery, some carry a much greater VTE risk (for example those with prolonged immobility, those undergoing emergency and/or open surgery, those with severe frailty, those with cancer, and those with a history of previous VTE) and would most likely benefit from extended prophylaxis^35–37^. A tailored approach that uses patient- and procedure-specific VTE profiles, such as the risk score produced by Lavikainen *et al*.^2^, should be adopted to identify those patients that stand to benefit.

In conclusion, in modern practice the incidence of postoperative VTE is low. Extended prophylaxis appears safe, yet the clinical efficacy remains uncertain. Further work is required to define patients who stand to benefit.

## Collaborators

**EuroSurg Collaborative and STARSurg Collaborative**

A Sgrò, R Blanco-Colino, N Brindl, D Chaudhry, K Gressmann, R R Gujjuri, A Hilder, E Matey, I S Pereira, A Turňa, C Varghese, W Xu, K A McLean, R Blanco-Colino, N Brindl, S Brown, W A Cambridge, D Chaudhry, K Gressmann, R R Gujjuri, A Hilder, A Jaffer, I Jakaityte, S K Kamarajah, M Kawka, O Kouli, E Matey, K A McLean, A Mergo, E C Mills, V Murray, Szy Ooi, I S Pereira, A M Riad, A Sgrò, S Q Shafi, I Trout, A Turňa, C Varghese, W Xu, B M Biccard, A Docherty, J Martin, K El-Boghdadly, M Phull, R Mouton, A Bhangu, J C Glasbey, E M Harrison, K A McLean, N Smart, S Moug, T Pinkney, T Richards, I Dajti, M de Cillia, G Van Ramshorst, S Delibegovic, H Mughal, J Mihanovic, N Gouvas, P Kocian, V Levesen, J Kaupilla, N Brindl, D Merz, M Joos, A Ioannidis, G Nnaji, F Pata, G Pellino, A Gori, M Podda, C Riboni, E Moggia, E Fekaj, S Oliver Senica, A Dauksa, J Psaila, F de Ruitjer, P Major, I Santos, M Sampaio Alves, J Simões, E A Bonci, A Pașca, A Novikova, B Kovačević, V Milosavljevic, B Tadic, J Kosir, R Blanco-Colino, S Pérez Ajates, M E Ossola Revilla, A Papadia, C Riboni, M L Gasparri, M K Aktas, B E Baki, M D Tepe, A U Mutlu, A Singal, J Osei-Bonsu, H Lacey, S W Chan, M Allison, K Duah-Asante, D Chen, N Ahmed, A Ejiz, C Takyi, M Mujeeb, N Ravikumar, M Khan, J Hayes, J Mckenna, J Wang, N Essa, H Xianghan, L Ko, Y Aldabbagh, J Plascevic, N Zia, R Ismail, Y Kamel, I Epanomeritakis, R Tan, N Chiu, A Naeem, M Kakwani, R Mehra, K Feeney, C L Yan Naing, A Qureshi, A Richens, H Li, R Ahmed, L Wilson, S Abraha, I Dajti, S Mikalauskas, J Kahn, D Kniepeiss, M Kušar, A Belarmino, J Frenk, I Mikalauskiene, A Angelis, J E Waha, S Al-Sharafy, M Sandano, P Schemmer, C Reiterer, K Horvath, A Taschner, S Riss, F Harpain, C Dawoud, N Hantáková, N Adamowitsch, S Schallmeiner, T Christian, V Xu, M M Kuhrn, K M Widmann, B Capek, B Kama, S Turgut, L Hauptmann, M de Cillia, M Grünbart, H Hoi, A Binder, T Gürtler, P Riedl, D Mayer, K Van Belle, O Wautelet, E Dekkers, E Van Daele, T Apers, G Van Ramshorst, F Berrevoet, N Rennie, P Dries, P Panta, A Denys, W Pype, T Bauwens, N Renard, S Violon, J Stijns, T Van De Winkel, E Van Eetvelde, M Salibasic, S Delibegovic, S Pusina, E Hodžić, M Kruščica, S Žilić, E Bicakcic, A Rovcanin, E Kulovic, E Halilovic, A Vinčević Hodžić, E Kalbić, S Delibegovic, A Mujić, M Mešan, E Matović, S Delibegovic, A Kesetovic, D Dardanov, E Arabadzhieva, B Markov, К Спасова, C Biji, N Oliveira, V Miroslavov, G Korukov, K Ravendran, N Shah, Y Mitkov, K Arivanandan, R Gaikwad, S Gotru, R Saraff, N Sain, D Stanchev, L Gaydarski, U Ali, L J Kandathil, A Krishnaveni, I Ivanov, I Ganev, Т Krapeshki, M Miza, Msk Mumu, I Bashliev, A Sajjad, L Chandel, D Yordanov, E Hristova, K Spassov, T Tsankov, T Ivanov, E Filipov, D Georgiev, V Neykov, E Daleva, I Ilieva, D Krasimirova, H Hristov, М Арсова, O Soladoye, A Biswakarma, R Raj, A Negi, A Dey, J K Adidela, U G Alionye, A Ndukwe, R Dimov, V Ivanov, S Sulcheva, S Mitrev, V Nikolov, M Karamanliev, D Dimitrov, A Yordanov, P Vladova, M Shoshkova, K Karakadieva, A Gabarski, A Shanker, H Ehtisham, S Parambi, L Tranchev, A Vasileva, J Kareem, J Ezeabasili, O L Madueke-Ediae, T Muradia, A Saini, I Rana, R Kanagaratnam, L S Thomas, M Galasyuk, J J Costa, N Majid, P Reddy, M Abdullahi, S Koshy Thomas, A Mehta, A Biswakarma, R Raj, S Shittu, J Theophilus, J Theophilus, R Ilyas-Uddin, I Ogunleye, E Naghavi, A Kiran, A Sundar, A Joy, F Ali, I Angelova, M Slavchev, N Belev, B Atanasov, P Krastev, T Yotsov, D Dimitrov, P Kamenova, I Mihaylov, A Stavrov, A Vricheva, G Šantak, L Penezić, Z Kastelan, Z Zimak, H Saić, N Knezevic, B Cikic, T Zekulić, T Hudolin, I Juric, J Anđelić, T Kulis, M Maric, J Mihanovic, T Soric, D Surić Grčić, L Blagus, P Kovačević, I Vidić, A Miočić Juran, C Ergatidou, S Gravas, A Yiallourou, S Achilleos, M Vardas, G Kokkinos, C Panagiotou, M Kyprianou, K Kyriacou, P Papatheodorou, K Konstantinou, D Onisiforou, F Tsoutsouki, A Kasapi, E Demetriou, A Stylianou, E Xenophontos, P Georgiou, P Makrides, I Pozotou, M Constantinou, T Evangelou, A Varavina, S Charitonos, M Lampi, M Attaalla, T Dušek, M Ndukwe, L Kuzmane, E Moorlata, H Sillah, J Kotek, J Fazal, E Ssali, V Steingauer, K Vinklerova, O Ahmad, Á Rodríguez Martínez, E Hiebert, M Srivastava, A S Ramesh Babu, O Dobra, A Akiba, M F Kalou, T Sokolová, N Nepeřená, P Novotný, J Sedlackova, F Philips, H Al Atassi, P Jose, A Sunil Nair, L Ntashamaje, A Kaddah, M A Antabi, P Kocián, R Fiala, T Harustiak, S Vesely, A Haluza, A Štekrtová, V Král, K Zdichová, J Pastor, A Gorchakov, H Novák, V Novák, J Hornak, M Havova, M Marková, T Fořtová, D Lérias Bento, R Novysedlák, J F Smetana, T Novák, T Zdobinska, M Švarc, E Hejduková, K Havlová, A Vaishevich, J Čorňáková, P Zajíc, P Francúz, O Příman, Š Havlíková, B Zemlickova, A Whitley, R Gurlich, P Balaz, I Tomyak, M Kútik, O Molva, D Šturc, M Belbl, T Vinklarkova, V Villefranque, A Police, E Mikhael, A Mabilia, L Charre, E Volpin, H Braham, R Montero Macías, D Krief, A Boyer De Latour, M A Angeles, A Martinez, A Navarro, G Ferron, M Del, M Ghiani, F Migliorelli, M Danguy Des Déserts, S Johan, B Olivier, C Andro, D Von Wedel, C Kamphues, M Thiele, J Felber, J Neudecker, C Schineis, J C Lauscher, R Stephan, L P Meyer, C Wolff, B Bombera, J Rolinger, A Kirschniak, P Wilhelm, S Göller, L Van Den Hil, D Reim, M Kießler, C Buschhaus, S Naisar, N Zahlmann, R L Walter, M Weber, K Müller, A Schweden, M Berlet, N A Jorek, S Seyfried, N Rahbari, E Birgin, C Reissfelder, A Reeg, P Téoule, E Rasbach, N Pyrgidis, G Hatzichristodoulou, I Sokolakis, J J Strotmann, T Fahlbusch, P Höhn, J Hinrichs, J Horn, S Jollet, J Binder, T Förtsch, M Winterstein, D Hackner, A Girard, M Wittmann, T O Vilz, F Recker, M Velten, G Massoth, A Delis, M I Brugués Villalba, M Willis, S Soltau, S Kudaliyanage, R L Eymael, I Syring, R Neubauer, N Dahmen, A Zimmermann, N Straßberger-Nerschbach, A Puskarevic, X Wang, L Peyman, R A Philippi, K Schmidt, U Bork, F Schepp, S Korn, O Radulova-Mauersberger, J Von Dem Bussche, F Von Bechtolsheim, C Praetorius, N Knobloch, F Oehme, J Weitz, M Mansfeld, M Distler, F Klingler, G Fluegen, S Dávid, M Schneider, K A Baba, U Ronellenfitsch, J Kleeff, J Ukkat, J Klose, O Bayram, M Sommerer, A Rebelo, I Gockel, E Gentsch, N Rayes, R Thieme, N Kreuser, B Aktas, D Branzan, D Seehofer, M R Mallmann, C Mallmann, S Woldu, F Balci, C Domröse, M Diekhöner, G Micha, K Stroumpoulis, L Dritsoulas, K Kalopita, G Bitzi, P Thessalonikefs, E Fradelos, D Korkolis, A Sarafi, D K Manatakis, N Tasis, A Prountzopoulou, E Tziava, K Pikoulas, D Paramythiotis, M Tishukov, K Papadopoulos, G Chatziantoniou, G Tzikos, S Papamichail, S Bareka, A Sarafis, D Haidopoulos, K Angelou, A Prodromidou, E Stamatakis, N Alexakis, V Pergialiotis, A Rodolakis, N Thomakos, N Memos, N Vlahos, I Koutalas, T Kotsis, E Kalampokas, L Chardalias, C Kontopoulou, C Lignou, P Lykourgioti, A Bagiasta, A Petrolekas, O Savranakis, T Kozonis, V Themelidi, M Mccormac-Prekeze, C Nikolaou, E Bletsa, N Melissaridou, K Flamourakis, P Rammos, T Hadjizacharias, N Provata, D Politis, A P Gkioulekas, D Massaras, I Vakos, A Antonoglou, P Dimitra, A Kotzadimitriou, C Evangelou, D Psychogios, M K Konstantinidis, K Apostolou, K Konstantinidis, N Patelis, S Konstantinidou, P Kokoropoulos, N Michalopoulos, Z Kratiras, P Drakakis, T Sidiropoulos, M Papadoliopoulou, P Vassiliu, N Arkadopoulos, C Koratzanis, N Vrachnis, E Dylja, I Chatzialis, D Sampanis, N Danias, A Fotiou, S Stavros, Z Petropoulou, V Tsaousis, D Papakonstantinou, P Lykoudis, C Nastos, A Charalampopoulos, I E Papiri, I Hatzaras, K I Paraskevas, G Petrakis, E Polenta, E Kaparounaki, M Sotiropoulou, S Kapiris, E Mavrodimitraki, A Paraskeva, A Kolinioti, M Psarologos, D Stergiou, P Metaxas, K Stamatis, C Kyzeridis, E Kefalou, G Z Vrakopoulou, A Larentzakis, E Menenakos, V R Maravgaki, C Georgiadou, M Koutrouli, A Papageorgiou, A Skandali, G Tzovaras, I Baloyiannis, V Tzortzis, G Christodoulidis, K Perivoliotis, E Arnaoutoglou, M P Ntalouka, A Chatzis, A Daponte, A Samara, C Donoudis, D Zacharoulis, F Mulita, K Bouchagier, G Verras, L Tchabashvili, O Ioannidis, A Koltsida, A Malliora, D Paparouni, S Bitsianis, L Loutzidou, E Anestiadou, Χ Χατζηανεστιάδου, S Simeonidis, A Tekelidis, K Zapsalis, S Skalidou, N Ouzounidis, G Ntampakis, A Kelepouri, F Kontidis, V Foutsitzis, O Kontaxi, G Barakakis, O M Valaroutsou, C Athanasiou, E Kotidis, M Pramateftakis, I Mantzoros, K Toutouzas, M Frountzas, T Triantafyllou, A Triantafyllou, T Dagklis, G Katsanos, S Petousis, A Athanasiadis, G Tsoulfas, K Dinas, I Tsakiridis, A Mamopoulos, I Kalogiannidis, F Rao, C Christou, S Vasileiadou, G Kapetanios, N Tsakiridis, S Kopatsaris, E Papanikolaou, K E Karakasi, T A Tataridou, N Antoniadis, F Zachomitros, A Arvanitaki, K Tsakiridis, M Anemoulis, S Neiros, K Ouranos, E C Tampaki, C Maltezos, K Maltezos, C Anastasiadou, A Chaveles, A Pachi, P Tsiantoula, K Roditis, A Antoniou, N Bessias, T Papas, S Tzamtzidou, D Maras, V Papaioannou, G Koukoulis, K Bouliaris, K Skriapas, G Kontopoulos, C Doudakmanis, C Kolla, M Efthimiou, C Kalfountzos, D Mitsakou, C Kardasi, S Zourntou, L I Fountarlis, A Bakalis, M Samarinas, P L Chatzilamprou, A Migdanis, K Karvouni, K Katsiafliaka, C Arvaniti, A R Papazisi, V Markatou, K Zervas, K Marsitopoulos, N Machairas, P Dorovinis, E Kotsifa, M D Keramida, D Schizas, M Vailas, A Syllaios, E Mela, N Hasemaki, A Skotsimara, A Katsargyris, S Kykalos, N Tomara, I Palios, I Karniadakis, A Charalabopoulos, P Sakarellos, S Davakis, N Kydonakis, G Tsourouflis, P Stamopoulos, K Laios, A Kozadinos, I Katsaros, E Kontis, C Iavazzo, L Katsiaras, E Kaouras, P Manikis, K Kokkali, G Theodorou, A Dragi, G Vorgias, L Tzelves, A Skolarikos, I Manolitsis, M Spartalis, I Tzima, A Anastasiou, P Pantelidis, G Schismenou, E Spartalis, G E Zakynthinos, K Lasithiotakis, G Petra, M Lampou, D Toth, Z Varga, J Pósán, L Illésy, C Váradi, M Santarelli, L Puca, L Brignone, R Lisa Marie, L Silvia, P Silvia, A Ferguglia, C Piceni, M Improta, F Assanti, E Montanari, G Ettore, F Cannone, L Cormaci, V Nicastro, R De Carlis, G Ferrari, A Giani, S Grimaldi, L Gregorio, M Mazzola, L Ripamonti, L Lorusso, G A Tartufari, C Magistro, A Benedetti, A Gonta, J Maesano, C L Bertoglio, I Giusti, O Quagli, F Brucchi, E Bevilacqua, L De Carlis, A Lauterio, R Cerchione, M Migliorini, N Incarbone, L Centonze, S S Darwish, I Vella, V Buscemi, A Podestà, M Desio, M Berselli, S Megna, E Cocozza, L Livraghi, V Quintodei, V Marchionini, G Borroni, C Peverelli, M Corbella, F Ferrari, F Odicino, E Gozzini, F Cisotto, G Baronio, R Del Giudice, G Sala, A Iacomino, G Vennarecci, D Ferraro, M Di Martino, D Pisaniello, F Falaschi, L Petagna, A Giuliani, P Di Lascio, M Coluzzi, G Pascale, P Gallicchio, G Frezza, G Dinatale, G Cerino, A Bottari, J Martellucci, G Maltinti, M Scheiterle, L Fortuna, F Staderini, F Coratti, M Veroux, A Giaquinta, G Roscitano, A Volpicelli, P Veroux, M Palumbo, G Riccioli, L Stella, C Distefano, R Gioco, G Lomeo, E Lomeo, M Cavallo, C Virgilio, C Molino, M M Giambra, D Zerbo, R Santini, S Costa, G G Incognito, D C Centonze, G Currò, M Ammendola, R Filippo, G Ammerata, R Balestri, L Morelli, D Tartaglia, M Puccini, V F Asta, V Conte, P Buccianti, B Sargenti, G Boni, S Signori, D Pezzati, L Urbani, G Casale, G Taddei, G Di Franco, M Bianchini, C Carpenito, N Furbetta, A Comandatore, F Tarasco, S Guadagni, M Mastrangelo, C Gianfaldoni, M Palmeri, L M Fatucchi, A B Boato, D Gianardi, M Stingone, L Sacco, S Giaquinto, L Lami, M Iuliano, E A Annunziata, M Chiarugi, S Strambi, F Coccolini, G Anania, M Chiozza, A Campagnaro, V Nevoso, L Carbone, L Marano, G Micheletti, A Fontani, S Malerba, M Gambelli, A Bombino, G Grassi, V D Mandato, L Aguzzoli, V Mastrofilippo, N Fabbri, C V Feo, M Ginestri, D Oppici, M Torchiaro, A Pesce, F Catena, C Vallicelli, V Murzi, M Podda, A Pisanu, F Campus, T Pilia, A Saba, C Piras, M Pisano, E Locci, P Marongiu, E Gessa, Mario D’Oria, A Biloslavo, S Lepidi, P Germani, C J Nappi, B Grando, A Lauretta, S Pollesel, P P Brollo, G Gallo, M Trompetto, A Realis Luc, V Tiesi, A Porcu, T Perra, M Madonia, A Tedde, F Scognamillo, P L Tilocca, D Delogu, G Mucci, G Drocchi, M Tedde, A M Scanu, G Rizzo, A Fancellu, C F Feo, G Farina, R Casu, M L Cossu, G C Ginesu, M Anania, S Dessole, G Capobianco, M Petrillo, F Dessole, I Angelone, A Barberis, A Azzinnaro, A Petrungaro, E Palli, E Mina, N S Pipitone Federico, A Muratore, M Calabrò, M De Zuanni, E Herranz Van Nood, L Licari, C Callari, D Di Miceli, S Viola, M Manigrasso, C Caricato, M Milone, P Anoldo, S Vertaldi, A D’amore, G D De Palma, A Marello, C G Cantore, A Chini, F Maione, G Luglio, G Pagano, F P Tropeano, M Cricrì, G Aprea, G Palomba, M Capuano, R Basile, U Bracale, R Peltrini, M Visconti, G Magno, A G Di Santo Albini, P Fransvea, G Sganga, G Tropeano, C Puccioni, G Altieri, V Fico, P Mirco, V Bianchi, V Cozza, G Pepe, S Alfieri, F Rosa, C A Schena, V Laterza, M M Pascale, S Agnes, G Bianco, F Frongillo, F Ferracci, F Santullo, A Balla, P Lepiane, F Saraceno, R Mastroianni, G Simone, G Tuderti, F Marino, G Lantone, G Lantone, R Isernia, F Pezzolla, F Aquilino, D Raimondo, A Gori, R Seracchioli, M Rottoli, A Belvedere, L Maurino, I S Russo, B Orsini, M Maletta, A Romano, P Bernante, T Violante, D Morezzi, E Degli Esposti, A Raffone, G Poggioli, A Canavese, G Dajti, S Cardelli, P Casadio, A Arena, G Sanna, D Cuicchi, L F Angelicchio, C Catalioto, B Torre, P De Iaco, M Tesei, E De Crescenzo, C Larotonda, F Greco, M Minghetti, L Sissa, A M Perrone, G Dondi, M Di Stanislao, L Gaetani, L Serafini, E Prosperi, D Perone, C Isopi, F Bruno, I D Alexa, P Milito, A Lovece, G Chiappini, C Froiio, T Panici Tonucci, G Saletta, A Scardino, A Belli, F Izzo, R Patrone, C Cutolo, R Palaia, V Granata, V Albino, M Leongito, M Piccirillo, G Pasta, P Delrio, D Rega, M Rho, A Aversano, I A Angelone, M D’amico, M Di Marzo, S De Franciscis, P Cianci, E Restini, I Conversano, G Scialandrone, S Di Saverio, L Cardinali, G Travaglini, E Sebastiani, I Marziali, A B Bellocchia, I Raimondo, D Di Giorgio, G Garganese, V Tondolo, A Dore, G Pacini, L C Turco, P Campennì, M A Amara, A Rubattu, D Verri, G Sole, S Bove, F Inzaina, F Franceschi, G Grande, D P Pili, F Campus, G Delogu, V Sula, O Ez Zinabi, M Congiu, E Pira, G Ercolani, F D’acapito, D Annunziata, L Solaini, A Giordano, S Cantafio, S Novello, M Romano, C Armellin, G Zanus, F Milardi, U Grossi, R Baldan, M Brizzolari, F Scolari, A Brun-Peressut, A Broglia, M Martorana, N Fazzini, C Marafante, M Garino, E Moggia, S C Agosti, A Battaglia, A Borello, S L Birolo, R Barone, C Mosca, K Shakhova, A L Apostu, S Giaccari, M Pavanello, E Migliori, D Sambucci, C Corbellini, G M Sampietro, G Germiniasi, M Bischeri, F Colombo, P Danelli, F Cammarata, R S Zingale, I Pezzoli, M Carbonaro, F Albanesi, F Cozzani, M Rossini, F De Gennaro, M Giuffrida, P Del Rio, M Inama, G Moretto, L Iudici, A Vitali, M Creciun, H Impellizzeri, M Piazza, A Biancafarina, M De Prizio, R Malatesti, R Sulce, G A Pellicano’, A D’ignazio, V Mariottini, G Mura, M Angelini, M Scricciolo, F Barbara, F Tofani, L Nenci, V Borgogni, C Vece, A Sagnotta, L Solinas, M Rossi, S Mancini, R Fruscio, C Ciulli, M Braga, F Romano, M Garancini, F Carissimi, E Vico, Cdg Delle Grazie, S Negri, J Corti, N Tamini, L Cigagna, R Giordano, A Davolio, G Trezzi, T Grassi, C Procaccianti, L C Nespoli, L Pitoni, A Fogliati, G Cordaro, P Passoni, M Fantauzzi, S Villa, M A Scotti, G M Di Lucca, F Benedetti, P Masseria, M Frigerio, M Barba, I Re, M Ceresoli, F Ferraina, S K Adjei Antwi, C Fumagalli, G Di Martino, M L Di Meo, L Bazzano, T Nelli, F Masciello, G Canonico, E Chisci, E Adinolfi, R Borreca, A Damigella, C Di Martino, Gil Mottola, G Fontani, S Michelagnoli, M Fedi, F Leo, R De Vincenti, C Cecchi, L Piombetti, S Giannessi, B Benedetta, M Pagani, L Epis, A Percivale, M Malerba, V Tonini, M Cervellera, J Shahu, L Sartarelli, A Luzzi, E Romairone, S Carrabetta, F Tuminello, F Floris, F Ré, S Marzorati, F Ballari, C Meola, A Filippelli, U G Ribeca, A Viacava, V Lizzi, M Montagna, A Giuliani, N Tartaglia, F Vovola, F Maffei, A Cotoia, M Pacilli, G Pavone, G Berardi, N Depalma, M Maruccio, M G Spampinato, S D’ugo, T Marchese, F Basurto, C De Giorgi, S Garritano, F Manoochehri, F Perrone, I Botrugno, A Rizzi, W Sergi, G I Russo, A Cappellani, S Cimino, M G Asmundo, M G Matarazzo, M Di Vita, P Venturelli, G Cocorullo, F Zarcone, D M Dominici, G Salamone, R Tutino, A Corigliano, R Guercio, C Giuseppe, F D’arpa, G Melfa, M P Proclamà, G Scerrino, I Canfora, M Marcianò, G Guercio, S Barbera, C Amato, A V Agostara, G Pantuso, G Orlando, N Finocchiaro, A Picciariello, D F Altomare, L Vincenti, G Tomasicchio, N Paradiso, A Dezi, E Pinotti, M Montuori, L Siragusa, C Pathirannehalage Don, G Sica, G Costanzo, V Usai, I Carrubba, M Pellicciaro, M Franceschilli, B M Pirozzi, F Santori, M B Busso, A Mariani Ivanikhin, A M Guida, B Sensi, A Divizia, F Blasi, V Giudice, L Orecchia, R Angelico, G Bacchiocchi, L Tariciotti, F Billeci, C Quaranta, C Cascone, M Materazzo, L Toti, T M Manzia, N Di Lorenzo, A Monaco, D Pedini, G Bagaglini, L Keçi, P Lapolla, A Mingoli, G Brachini, B Cirillo, P Sapienza, P M Cicerchia, C Leonardo, E Bologna, L C Licari, M Zambon, G Mazzarella, A Falasca, G Duranti, R S Flammia, V Asero, A Bernardotto, F Scarno, S Meneghini, S Giovampietro, B Binda, A Tufano, V Palombi, E De Meis, M Rocchetti, C De Padua, M Mansi, P Bruzzaniti, P Familiari, S A Nottola, F Fleres, E Cucinotta, S A Biondo, F Viscosi, N Catarsini, C Mazzeo, M Iannello, V Testa, A Gattolin, R Rimonda, F Riente, E Travaglio, F Allisiardi, P Guffanti, F Ferrara, M R Barbieri, A Puzziello, A Mollo, E Donnarumma, F Steccanella, A Oliviero, G Lopez, C M Di Maio, S Olmi, F Di Capua, A Camillo’, A Carresi, M Uccelli, C Rubicondo, R Rosati, U Elmore, F Puccetti, D Socci, M Clementi, A Giuliani, S Tontoli, A Nisi, A Grasso, S Derraj, J Di Biasi, I Tucceri Cimini, C De Nunzio, S D’annunzio, A Nacchia, G Gallo, G Lisi, D Spoletini, S Signore, R Menditto, M Carlini, D Sasia, E Dalmasso, A Trucco, E Olearo, M C Giuffrida, I Morra, M Maione, A Puppo, A Marano, G Preve, B Vercellone, E Frola, E Beltrami, I Peluttiero, G Giraudo, M Migliore, S Armentano, E Alessandria, A Daniele, P Elisa, L Bonino, M Borghese, D Bono, G Canova, M Zago, A Nicotera, L D Bonomo, L Gattoni, N Cillara, A Deserra, A De Lisa, E Coccollone, A Cannavera, R Cabula, F D’agostino, M Deplano, C Chillura, M L Robuschi, A Borzacchelli, C Margiani, M C Murru, G Pellino, E Lieto, F Cardella, A Auricchio, Smc Erario, G Del Sorbo, O Lidia, M D’ambrosio, F Menegon Tasselli, S Mastroianni, F Selvaggi, G Calini, G Terrosu, L Clocchiatti, V Morinelli, C Valotto, F Traunero, G Vizzielli, S Restaino, E De Gennaro, U Baccarani, A Andriani, J Mauro, P Frigatti, E Martin, S Pregnolato, L Driul, A Zullo, G Carcano, L Latham, D Inversini, A Vigezzi, G Ietto, R Marta, A Marzorati, E M Colombo, S Garbarino, N Palamara, M Tozzi, G Piffaretti, M Franchin, L F Festi, M Odeh, M C Fanelli, J Costa, V Pappalardo, A Scorza, M Cannavò, A Ballabio, A Barina, B Franzato, L Rubin, D Brugnolo, O De Simoni, S O Senica, A Ozoliņš, E Vjaters, N Jain, R R Apse, A Truskovs, I A Apse, P Jukonis, J Jukonis, A Tumova, H Sivapalan, Oss Piirtola, A E Berga, H Tumegård, N Samalavicius, V Nutautiene, O Aliosin, V Eismontas, A Dauksa, L Venclauskas, K Jasaitis, D Lazauskaitė, K Urbonas, V Šlenfuktas, I Grikytė, M Jokubauskas, V Simanaitis, Z Dauksa, R Gudaityte, R Riauka, U Krunkaitytė, S Svagzdys, A Šikarskė, J Pakrosnyte, E Stratilatovas, A Piscikaite, G Žaldokaite, T Poskus, V Olekaitė, J Psaila, P Andrejevic, C Cini, K Cassar, S Mattocks, M Sammut, S Mizzi, L Abela, A Micallef, K Pace, B Schembri, R Cini Custo, M Harmsworth, B Farrugia, F Theuma, R Gatt, G Montebello, N Spiteri, J Galea, L Vassallo, L Fava, A Ebejer, K Carabott, K Iles, L Casingena, G Apap Bologna, G Debono, J Galea, M Sammut, S Huisman, W Leclercq, F De Ruijter, J Konsten, D Adramanova, A Nikolovski, A Otljanski, M Kisielewski, K Richter, N Kłos, T Stefura, K Macheta, I Alsoubie, J Tempski, N Wolińska, W Wysocki, E Starek, N G Nowak, R Tarkowski, G Wallner, J Kobak, E Mączka, K Żak, A Ziółkiewicz, K Kułak, E Langa, Ł Łaba, I Krzesińska, M Bobiński, K Frankowska, S Dziurda, J Martyna, J Radulski, O Lulko, J Tomczyk, M Jasiński, P Major, J Rymarowicz, Z Zielińska, K Siuda, M Kęska, P Wites, P Błasiak, A Sierżęga, J Kuciel, D Tomczak, B Molasy, P Nieroda, E Buras, R Gonçalves Pereira, S Patrocínio, S Reis, C Rolo Santos, L Moniz, P Bernardo, C Osório, L Matos, L Carvalho, J Marques Antunes, N Alçada, A Marçal, N Tenreiro, D Martins, C Leal, C Ferreira, R Marques, F Freitas, C Marques, A Melo, S Silva, B Vieira, U Fernandes, B Freire, S Fonseca, M Reis, C Macedo Cardoso De Oliveira, J Mendes, M Carvalho, J Macedo, J Oliveira, C Mexedo, D Sousa Silva, J Davide, E Emanuel Do Vale Gonçalves De Castro Alves, M Ginestal, M D Santos, J Santos, C Robalo, V Valente, J P Fernandes Dos Santos, I Mesquita, B Teixeira, M Marques Monteiro, M I Silva, M Nunes Coelho, M Teixeira, R L Silva, L Lopes, A Ribeiro, C Lima Da Silva, T Correia De Sá, M Martins, M Costa, C G Gil, M Barros, R F Santos, A Silva, J Pedro, M Costa, T A Ventura Antunes, M Pascoal, R Andrade, M Duque, I Prior, M Lemos, P Pinto, P Guerreiro, S Castanheira Rodrigues, E Barbosa, A L Martins, A Pereira, S Vaz, A Fareleira, J Rocha-Neves, L Dias, F Girão De Caires, A Pereira-Neves, A I Oliveira, J P Araujo Teixeira, J Ferreira, M Almeida, M Vb Machado, J Nogueiro, F Gomes, E Campos, F Jácome, C Coutinho, R Ribeiro Dias, J P Vieira De Sousa, E Francisco, C Borges, S Pereira, C Pereira, N Machado, R Calaia, J Pinto, T Corvelo Pavão, L G Santos Sousa, F Cunha, D Melo Pinto, R Pereira, I Dinis, C Ferros, J L Pinheiro, A J Santos, B Barbosa, D Gaspar, M Pinto, D Pereira, N Araújo, B Alves, H Barbosa, D Silva Araújo, M Afonso, B Guimarães, M Campos Coroa, M J Susano, J Azevedo, M Pereira, P Miranda, R Garrido, M Oliveira Mourão, M Ferreira, A Duarte, P Félix, I Antunes, N Fernandes, C Gil Corrêa Figueira, I Figueiredo, M Gututui, S Oliveira, A Silva, F Madeira Martins, A Cabral, C S Rodrigues, B Gama, R Silva Borges, R Lourenço, A C Longras, R Araújo, I Peixoto, A Santos, D Matos, A Lopes, L Claro, D Cardoso, A Martins, M Silvestre, G Sousa, M Santos Bessa, V Cardoso, C Velez, A Machado, R Pedroso De Lima, M I Matias, S V Matos, M Rocha Melo, J Bolota, M Rente, A Santos, M L Antunes, A Bárbara, E Rosin, J Oliveira, S Leandro, M Damasio Cotovio, S Morgado, G Fialho, N Andrade, F Valente Costa Pinto, H Capote, S Morais, M Buruian, F Taré, G Santos, D Rosado, C Costa, T Mogne, N Pratas, B Cordeiro, M Brito, D Salvador, S Carvalho, V Capella, M Carracha, R Alves, C Miranda, F Rebelo, T Teles, M Ferreira, B Cismasiu, P M Costa, S Henriques, M Trindade, J Vaz, M Gomes Vieira, M André, A Macedo, J G Gonçalves Nobre, J Prosil Sampaio, A R Mira, A L Preto Barreira, P Botelho, D Melo Pinto, C Quintela, M J Quelhas, D Pereira, A Cruz, C Mesquita Guimarães, C Pinto, F Maldonado, F Sales, F Marrana, A R Reis Aguiar, L Freire, L Farias, G Faria-Costa, A Castro, T Moreira Marques, G Cardoso, J Ribeiro, M Fragoso, C Silva, M Vasconcelos, M Picciochi, A Sampaio Soares, J Frazão, R Miranda Pera, F Gaboleiro, F Ramalho De Almeida, A Mendes, F Afonso, J Fontaínhas, M Reia, M Angel, C Aguero, M Guerrero, J Dominguez, I Borges Da Costa, L Ramos, J Fernandes, C Assis, F Fonseca, S Carvalhal, A Caiado, F Brito Da Silva, B Miguel, J Moniz, M Pires, B Leal, J Nunes, F Matos Sousa, T Vieira Caroço, M Romano, M Ângelo, A Rodrigues Ferreira, A R Prata Saraiva, F Mano, F Rodrigues, T Branco, S Gaspar, F Neves, M Alves, A P Ferreira Pinto, M Peyroteo, C Baía, M Marques, A M Correia, J O Silva, R Monteiro, P Silva-Vaz, J Gomes, M J Moutinho Teixeira, T Brito Neves, F Meruje, C Sheahan, D Macnamara, J E Linares Gómez, S Clare, A Walmsley, A Tiwari, T Khan, G Dowling, K Hudak, E Craig, A Dhannoon, S Saeedi, J Shah, M Jordan, T Buckley, J Engler, M Reilly, J Ariaratnam, M A Hinn, K Benn, S Petropoulos, H S Hansen, S Browne, S Keelan, M Basta, R Pornsakulpaisal, G Tan, A N Niyaz, N Lakic, T Warhust, Y Q Chang, Y Qaoud, D Mwipatayi, D Kearney, C O’reilly, Y Gamper, R Dhillon, V Patel, K Gallo, H Mulvaney, P Edwards, S Tiller, E Gorecki, M Jordan, S Daswani, V Jones, I Asekomhe, N Clausen, M Khan, A Feldiorean, A Pezeshki, T Khedro, J Beyer, G Byrne, W Irfan, A Poluha, C Fager, L Gilligan-Steinberg, J Yarp, E Byrne, M Lorico-Rappa, A Hassan, T Keogh, W Denning, K Horton-Schleicher, T Deane, A Haren, A D Md Hamsan, N Mukerji, M Dwivedi, Sml Cheah, P X Kwek, S Arshad, A Alzaki, I Cornila, H M Feizal, M Barry, A Semar, P Gorman, A Gordon, C Mcsweeny, N Layyous, D Peiris, S Pan, J Larkin, M M Umar, C Doherty, C Mitchell, H Mcelvaney, Z Sabz Ali, S Adesokan, A Murphy, A Yeo, L Hayes, C Owens, N Crawley, E Macinnis, S Elekes, K E Oderoha, I Mac Mahon, B Moran, P Matreja, K Conlon, P Piankova, M Z Farran, S V Kashyap, M Puntambekar, M Sampat, S Heaslip Owens, A Maheshwari, A Soo, J Butt, K O’brien, M Almutairi, C Mcfeetors, M Kerin, M G Davey, W Murray, A Nasehi, A Graham, C Mathew, S Azam, K Chua Vi Long, S Stafford-Johnson, D Ejaz, Z Siddiqi, Amh Kon, M Parmar, C Peirce, D Shomoye, A Alabi, N Salzberg, V Gallippi, J Aziz, C Melly, H Gill, H W Yang, S E Eltahir Ibrahim, Jsk Rugber Singh, N Bacalbasa, I Balescu, M Muresan, V Dudric, I Imihteev, V Chelaru, T M Băiceanu, M Alexandra, A Bashimov, M Voda, D Muresan, R Varadi, M Muntean, Ș Macovei, R Checiu, D Buf, F Grama, A Chitul, C Bezede, E Ciofic, A Beuca, V Bintintan, D Muresan, A Raluca-Cristina, R Rad, S Șușman, C Suciu, J Abu Arif, A David, M Blaga, B Olesea, D A Butnariu, R R Scurtu, R Rad, P Claudia-Mihaela, M Girlovanu, D Costina, R Drasovean, G Tartiu, S Tache, N Irina, A Streang, A Trif, I Romascan, E Stănică, E Vass, C Cucoreanu, E A Toma, V Calu, O Enciu, A Laceanu, R Maria-Zamfirescu, I M Matache, I Mușat, D C Piriianu, A A Georgescu, A Miron, M Tartalea, N Al-Ugeily, B Bogdan-Gabriel, A Alexiadi, A Zaharescu, C Ciubotaru, I Negoi, M Paunescu, I Iancu, I V Pop, B Diaconescu, P Dao-Zamfirescu, N B Patel, S Rafi, D Popescu, M Girbaciu, A Rădoi, C Breuer, A C Braun, B Stoica, I Gîlia, A Gheorghe, E C Popa, F Brichius, A Bucurica, V M Negoita, M Radu, R Bianca Ștefania, C L Socol, A M Baiceanu, S I Bubenek Turconi, L Valeanu, M Girel, M R Gavrila, M Pirvu, B Morosanu, M Ioniana, A Barbu, R Cornel, B Bogdan, T Bute, L Madec, E Burla, C Balan, E Alexandru, D Tomescu, M Popescu, M Dumitru, A Marinescu, I Petrusca, A Pasca, P Achimas-Cadariu, A Irimie, A Petrusan, M Puscas, S Titu, N M Jiboc, I C Vlad, D T Eniu, P Ciubotariu, V A Gata, A Rancea, C Iulia, D S Morariu, C C Nistor-Ciurba, I Gale, G Lazar, E Bonci, F Ignat, C Lisencu, C Dumitraș, F I Faur, M O Butuza, M C Marian, A Paul Luchian, H Bocse, I C Puia, C Andra, P Pop, S Moldovan, S Ursu, M Sorin, S Lunca, M Dimofte, A Musina, N Velenciuc, C E Roata, S Morarasu, M Velescu, F Mureșan, I Zaha, I M Rusu, B Mircea, D G Tauberg, M Kirov, V Kuzkov, A Nikonov, A Tishchenko, D Kulin, V Mironov, A Litvin, I Gunko, I Mazhega, R Bilenko, G Khrykov, Н Манкевич, M Karpenko, E Zagaynov, A Shilyaev, A Butyrskii, A Tatidze, Z Seytnebieva, A Aliev, M Rumyantseva, A Yanishev, A Abelevich, S Doktorov, K Zuev, A Lazareva, A Malov, D Borisov, Е Борисов, И Андрийчук, Z Galchikova, M Shemetova, K Maltsagova, A Bedzhanyan, K Temirsultanova, D Efremov, A Volkova, Y Frolova, A Minenkova, K Petrenko, A Sumbaev, M Bredikhin, E Bedzhanyan, A Mikhailova, В Тен, Ю Кудрявцев, A Novikova, R Pavlov, M Chernykh, N Boiko, В Данилин, G Stanojevic, B Brankovic, M Nestorovic, N Milutinovic, A Vukadinović, A Vukadinović, B Stojanovic, D Radovanovic, A Cvetkovic, I Radosavljevic, M Sreckovic, B Milosevic, N Markovic, M Spasic, M Pavlovic, D Lazic, R Vucic, B Tadic, B Kajmaković, Z Vilendecic, D Knezevic, U Bumbasirevic, A Stefanovic, P Gregoric, Z Perišić, D Micic, S Ratkovic, J Vasiljević, P R Bulat, V Jovanovic, O Mitrović, J Gunjic, D Potkonjak, N Grubor, M Reljic, I Palibrk, V Milutinovic, M Zivkovic, D Škrijelj, Ž Grubač, S Kadija, R Cerovic Popovic, L Andric, L Aleksić, S Kmezić, I Pavlovic, M Djukanovic, B Milojevic, Z Dzamic, M Petrovic, K Jeremic, I Pilic, B Milosevic, J Krstic, V Šljukić, M Veselinović, N Ivanović, A Janičić, A Jovanovic, D Cvijanovic, I Ladjevic Likic, M Radojevic, J Ćupić, I Dimitrijevic, M Miladinov, A Sekulić, D Nektarijevic, S Pantovic, D Šljivančanin, S Dugalic, A Đermanović, Z Radovanovic, D Radovanovic, M Đurić, S Zahorjanski, V Milosavljevic, D Zdravkovic, B Toskovic, U Marjanović, M Zdravkovic, S Petricevic, B Crnokrak, S Maric, A Sekulic, N Colakovic, B Kovacevic, I Krdzic, J Bojičić, T Sparic, A Ostojic, A Cokan, J A Košir, M Pakiž, S Potrč, N Kavčič, A Ivanecz, M Horvat, T Jagric, U Kacjan, I Perić, E Timošek, I Plahuta, Š Turk, B Crnobrnja, R Kovačič, A Dovnik, L Al Mahdawi, J Knez, J Grosek, T Košir Božič, A Tomazic, L A Suarez Gonzalez, S Busto Suarez, C De La Infiesta García, O Arencibia, D Gonzalez Garcia-Cano, M Laseca, A Martin Martinez, J A Guijarro Guedes, A F Rave Ramirez, Y Gil Gonzalez, A M Hernández Socorro, D Ponce Arrocha, A Cruz García, C Mendoza Rodriguez, E Catala, C S Romero Garcia, A García Trueba, V Georgieva, A I Hernández Álvarez, C Martinez-Perez, E De Valera, J J Gascón, E Lopez Alcina, L Samper Monton, K Aghababyan, A Valero Tarin, C Salinas Lozano, A Cervera, J C Catalá Bauset, B Ramia, F Marques Peiro, J De Andres, S P Iserte Juan, M Peñalver Gaspar, J Gilabert-Estellés, M De La Rosa-Estadella, A Fernandez-Colorado, M Serrano-Martin, A Gasulla-Rodriguez, M Juarez-Pomes, M E Ossola, G Sanz Ortega, J Gómez, M Del Campo, R Corrochano, M Galan Hernandez, F J Molano Nogueira, L Ibañez Vazquez, J Dziakova, A Zarza Martín, S Infante, D Moro-Valdezate, L Pérez Santiago, A I Molina, A Vinuesa Huesca, R Gadea Mateo, L Navarro-Sanchis, P Aracil-Boigues, C Jezieniecki, B De Andrés-Asenjo, Á Zamora Horcajada, F Natal Álvarez, L A Cuellar Martin, M Montes-Manrique, G Cabezudo, T Gómez Sanz, A Herranz Arriero, N Sierrasesumaga, M Rodriguez-Lopez, M Ruiz Soriano, A Sánchez Gollarte, A M Minaya Bravo, C Guijarro Moreno, A Galvan, E González, M Á García Ureña, A Robin Valle De Lersundi, J Ruiz-Tovar, A López Campillo, M Jimenez Toscano, S Salvans, S M Jaume Böttcher, À Godó, G Busquet Raich, C Téllez, J A Sánchez García, M Ribas Ardanuy, P Saavedra, A Sánchez Cabrero, J Clivillé-Pérez, M Durante Escutia, A Rabasa Rodríguez, P Aguilera, C Giménez Francés, M Ruiz-Marín, D García Escudero, M Valero Soriano, M Ramirez Faraco, S Galán Jiménez, P Alcón Cerro, J M Rodríguez Lucas, M Carrasco Prats, E Peña Ros, Mdp López Sánchez, V J García Porcel, M Tamayo, J M Muñoz Camarena, R Albarracin Garcia, O Molina, P Moreno Sánchez, I Jiménez, P Pastor Perez, M B Agea Jiménez, D Candela Mas, M Artés Artés, J A Benavides Buleje, F M González Valverde, P López-Morales, A Sanchez, E R Bobadilla Romero, M Allue, L Ponchietti, R Latorre Tomey, D Torres Ramos, L M Jimenez-Gomez, S Valdés López-Linares, Y T Moreno Salazar, J Soto Galán, C Moreno De Alboran, A Prosperi, J J Osma, A Blanco, M Sánchez Rodríguez, E Valdivielso, Á Cejudo, C Perez Carpio, F Vasques Seabra Águas, E Sánchez Martín, S Pérez-Ajates, E Macarulla, A Álvarez Torrado, C Galmés, M Artigot, J Marin Garcia, J R Oliver Guillen, A López De Fernández, B F Fernández-Velilla San-José, D Ambrona Zafra, S Pérez Farré, J Ortega Alcaide, L Codina Corrons, E Sisó Soler, F D Gómez Báez, G López-Soler, E Gutiérrez Pérez, S Aix Molina, M Vallve-Bernal, A Varona Mancilla, Á M Aldama Martín, R Casanova, L Sevillano, J J Muela, M Rodrigo Rodrigo, I Goujon, C Fructuoso Iniesta, S Pérez-Bertólez, E Monclus, L Fernández, M G Silva Hernández, F Vicario, P Garcia Pimentel, R García Álvarez, N Bouzó Molina, Á Ramiro, Z A Calderon Barajas, R Sanz, Z Xia, P Rodríguez, C Sarrais Polo, A Martínez López, C Estrada, P E Gómez De Castro, D Fernández Martínez, L J García Flórez, I De Santiago Alvarez, L García Alonso, G García-Santos, L A García González, B Carrasco, D W Silva-Cano, P Del Val Ruiz, G Martínez Izquierdo, A Corteguera, J Iturbe, E López-Negrete Cueto, A Cembellin, C Ramos Montes, G P Ibero Casadiego, M Prieto, I Villalabeitia Ateca, T Pascual Vicente, B Villota Tamayo, A Perfecto, S Mambrilla, I L Ramos Del Moral, V Jiménez Carneros, P Pastor-Riquelme, A Franco Lozano, L Alonso-Lamberti, M Lozano, J García-Quijada, R Zhang, A García, M M Martín, M Yeh Ahumada, A Carreño Pallarés, Á Pérez Rubio, R E Goran, N Gómez Diez, G Zomeño Bravo, J C Bernal-Sprekelsen, G S Martínez Fernández, R Marquina González, C Toribio-Vazquez, A Eguibar, H Perez-Chrzanowska, P Serrano Méndez, S Valderrabano Gonzalez, F J Reinoso, A Yebes, M B Alonso Bartolomé, H R Ayllón Blanco, I Cristóbal, I Pellicer, R Arenal González, J D Zafra Angulo, V Duque Mallén, C Gracia-Roche, I Gascon Ferrer, D Aparicio-López, M Antonio, M Herrero Lopez, S Saudí-Moro, M Á Gascón Domínguez, T Gimenez Maurel, B Matías-García, N Morales Palacios, M Diez Alonso, A Gutiérrez, S Soto Schütte, M Bru-Aparicio, R Jiménez Martín, A Sanchez Pellejero, S Vázquez Valdés, C Vera Mansilla, A Quiroga, F Mendoza-Moreno, L Diego García, E Serrano Yébenes, F Mañes Jiménez, I Lasa, E Ovejero Merino, L Casalduero, Y Allaoua, A Blazquez Martin, D Córdova García, R Alvarado Hurtado, P Laguna Hernández, F Garcia-Moreno Nisa, H Juara, C Aldecoa, F J Tejero-Pintor, M García, E Aguado Saster, E Ruiz De Santos, A D Bueno Cañones, S Pérez Fernández, M G Alija Garcia, R Urruchi, R Rioja Garrido, M Madrid, A Bordell, I Arranz Chamorro, P Rodriguez Cañal, D Pacheco Sánchez, P Marcos-Santos, S Veleda Belanche, J L Maestro De Castro, M J Blanco García, E Laita Jiménez, L Leal, L Vaquero Perez, C Barbosa, M Ramos Carrasco, I Garcia-Saiz, P Rodicio, F Acebes García, A Lizarralde, A Sanchez Diez, E Ferrer-Inaebnit, J J Segura-Sampedro, M Alfonso Garcia, Ggc Gutiérrez-Cañadas, A Oseira, N Torres Marí, J Loyola, B Villalonga, P Camporro, N Pagès, E Colás-Ruiz, J A Cifuentes Rodenas, M Castro Suárez, R Moll-Amengual, J Mata, J Fernández Manzano, B Gómez Pérez, J Gil Martínez, Á Cerezuela Fernández De Palencia, A Aliaga Rodriguez, P Gómez Valles, A Delegido García, E Gil Gómez, M J Montoya, A Navarro-Barrios, V Cayuela, J J Ruiz Manzanera, M I Jiménez Mascuñán, A I Gutiérrez Fernández, I Sánchez Esquer, P J Gil Vázquez, D Ferreras, A Balaguer Román, F Gómez-Bosch, F Alconchel, A Romero, V M Durán Muñoz-Cruzado, C J García Sánchez, A Tejero Rosado, B Ruiz, Mdc Roman, J J Rubio Fernández, Á De Jesús, M Ostos Diaz, C Quintero-Pérez, A Sánchez Marín, M Alvarez Aguilera, M Leal Mérida, I Ager, J Sancho-Muriel, M Frasson, B Castro, P Guerrero, M Nieto-Sanchez, Q Cruz, H Cholewa, L Hurtado-Pardo, D Plazas, M Serrano-Navidad, J Castillo, R Ballestero, V Valbuena Jabares, C García, N Garcia Formoso, E Herrero Blanco, N Suarez Pazos, C Cagigas Fernandez, M Gomez Ruiz, Y T Benic Yoris, M Merayo, M E Gonzalez Fernandez, S Alonso Batanero, J Rodríguez Gómez, A Landaluce-Olavarria, B Estraviz, I Markinez, D Gómez, L Fernández Gómez Cruzado, M González De Miguel, J M De Francisco Rios, A Urigüen, A Gómez Del Pulgar, E Espin-Basany, A Gil-Moreno, S Bellmunt-Montoya, C Dopazo, S González-Suárez, R Blanco-Colino, P Olid Mayoral, M González Antúnez, E Del Barco Martínez, Y Bejar Dolcet, D Gil-Sala, C Marrero Eligio De La Puente, L Sánchez Besalduch, M Barrio Zaragoza, J F Lopez Lozano, C Olaria, S Catalan Sanz, E De Ciria, L Segura Farran, N Montes, J M Zanca Gómez, I Puig Escobar, M Armengol Alsina, M San Nicolas, M Escura, M Sánchez, M Rivas Agudo, L Aalouf, D Maya Salas, L Hernando Marín, N Umpiérrez Mayor, J L Sanchez Iglesias, Y Fernández Henriquez, I Feixa Molina, M J Gomez-Jurado, V Bebia, M Racine, M Sauvain, A Mujcinovic, D Bolla, Y Dimitrov, N Arenja, C Riboni, J N Marx, M Kalisvaart, C Andreou, P Brandts, U Pfefferkorn, L Eisner, U Dietz, J Gass, J Metzger, A Scheiwiller, V Kremo, D Gero, M Bueter, F M, R Schulz, F Mongelli, I Soave, D La Regina, C Canonica, F Sabbatini, P Gaffuri, S Spampatti, C Santarelli, M Di Giuseppe, M Marengo, S Passoni, G Dellaferrera, D Provenzi, L Bernardi, A Papadia, A Cristaudi, M L Gasparri, P Aurora, S G Popeskou, P Christian, M Antonilli, F A Scalvi, V Sevas, G Palumbo, L Maoloni, C Cencetti, G Pagnani, M Hitz, J Dürmüller, S Däster, G F Hess, Nle Aegerter, P Sedlaczek, S Soysal, J Zeindler, F Angehrn, L Müller, S Taha-Mehlitz, F Haak, F Nocera, T Karli, I Lazaridis, A Tampakis, A Lalos, U Friedrich, B Wiesler, N Varathan, R Frey, G K Kurtoglu, A Aghayeva, C Turam, G M Kurtoglu, M Maarouf, B Baca, S D Ilhan, Y O Koyluoglu, M E Seker, M Ulufi, M Erkaya, Fdb Kılıçkan, S Bayrakceken, B B Ozmen, B Togay, M Doğan, T Çetinkaya, E Tuzuner, T Karahasanoğlu, I E Yavuz, I A Bilgin, S S Kekik, C Turam, E Dönmez, M Dikeç, N Ramoğlu, N Yurtseven, O Takmaz, Ö Orhun, N Karadeniz, O Saylık, E M Uğur, E Ada, A Durbas, I Hamzaoglu, K Uzun, M Gungor, I Y Gebedek, E Ç Hayırlıoğlu, M Halıcı, G O Ceyhan, A U Mutlu, E Aytac, M Gulmez, E Capkinoglu, T U Yilmaz, A Ozer, S Keçeoğlu, Z C Eraslan, V Ozben, O Dülgeroğlu, C Saraçoğlu, M Erkan, C Uras, H D Copur, N U Dogan, E Topal, I Demirsoy, D Korkmaz, U Can, D Buğra, E Sobutay, H Çakıt, E Ergün, S Zenger, C Bilgic, B Gurbuz, K Sünter, I Gecim, S Sefer, B Inceöz, M B Genc, A K Uygun, E Yücel, A Barcin, Y Altinel, Y E Aktimur, S Meriç, K Özdoğan, A I Sayar, A G Durmaz, I Çakır, E S Ünlü, G Kiran, A Dal, E Ozkan, S Kalkan, M M Öncel, Ç Çetin, G Yılmaz, H Karimi, Y Iskurt, E Yardimci, M Eker, A O Balkan, H B Gönül, E Herdan, M Ertugrul, T Takmaz, S S Kilinç, M S Pinar, F Yıldıztekin, E C Coşkun, I Olgun, G Ince, O Isik, M Şen, R Özata, B Eroğlu, B Büyükpolat, G Ishakoğlu, D Özen, M O Aktaş, B Kılıç, T Yılmazlar, M S Koçbey, N Işıklı, R G Yıldız, T Baghirov, E Uygun, M Yugaç, E Keskin, B E Bozkurt, S Tas, Ö Ö Türkmen, B Ata, B Alan, N Işık, T Göver, T Bisgin, E Özalp, S Sökmen, B Manoğlu, C Bakar, O Bozbiyik, U Zorlu, N N Zengin, B E Baki, E S Akbulut, S Tunali, B Kutlu, Y A Oğuz, B Küçükateş, E Yildiz, A N Sakmar, T Yoldas, Z E Akgün, M A Korkut, C Çalışkan, E Aksit, E F Aksalman, A O Koçoğlu, F Basci, B Yigit, O Ilhan, R K Liman, M Uzun, I Ağaçkıran, C Şahin, S Leventoğlu, Ö Kubat, I Genç, A Oyanik, F I Gurbuzer, N Satilmis, S Cam, M Ekinci, M Gönül, D Chasan, Sgf Zara, Ş Sök, A Yalcinkaya, E T Acıpınar, U Timurçin, N B Afzal, Sha Gillani, S Gillani, S Yazıcı, O Cennet, M S Suer, Ö Kaya, E Z Uslu, A Tanrisever, C Yildirim, I N Gunenc, B Gül, I Özkal, H I Tileklioglu, M A Korkmaz, I Şirin, U Özbayrak, A Mıdık, E Domaç, A Karakoç, S Urek, A Yıldırım, M Ugur, M Doğru, E R Arslan, B Ular, M E Duymuş, A B Turhan, T Dogan, M B Ozkan, D E Benek, M Arslangilay, M B Yildirim, Y Yazgan, H N Tıraş, B I Şahin, M A Çapar, C Tatar, A Arı, O Batikan, M Güler, A E Naycı, M M Sevinc, H Şevik, C Oğuz, O O Karagülle, U O Idiz, A Demir, E Çakır, C Cakir, S Doğan, I Yıldız, M Gürlük, M B Cengiz, M Toptaş, O Akay, R Kaya, E Aydin, M Ç Çakıcı, A Yildirim, Ö Efiloğlu, B Demirtaş, A Iplikçi, G Atis, F Keser, M Culpan, M Çiçek, A Tahra, M K Akalin, A Izgiş, M Soytas, N Okkabaz, A Saylar, E Onaran, A E Askin, E Hashimov, S Bektas, K Sabuncu, A C Alagöz, I Karatas, Afk Gok, E Simsek, Z I Altunay, E Koç, Y Iscan, I C Sormaz, B I Yabaci, M S Ertürk, M Nazli, I E Saglam, E Erginöz, M F Ozcelik, M Gokden, C Guler, F I Gunaydin, S S Uludağ, B Ibis, H O Bozkir, M B Karaca, M C Ulucay, K B Oner, S Akbas, F Z Calikusu, H Akcan, S Ugural, A K Zengin, Z Ozdemir, I Demirbas, A Guner, M D Tepe, N Kıralı, B C Karabağ, D Pehlivan, A N Yuzgec, B Yıldız, B Akin, M Aktaş, E Karabulut, H Cepe, H Küçükaslan, S S Salih, M Uzun, Ö Yücesan, M E Reis, M Ulusahin, K Aşcioğlu, E Tufan, Q Saleem, K Saracoglu, M Ozbilen, A Kale, M E Geçici, Y Bulun, Ö Uysal, A Atay, B Şuataman, C Guducu, T N Çinar, H Kul, D Canpolat, M Ipekoğlu, M Aydemir, F Karahan, K Hizli, B Kaya, O C Yenen, B Gümüş, K Turmuş, S Vatansever, I Candan, S Sucu, E Balik, E Bozkurt, I H Özata, E Ozoran, T Tüfekçi, S N Karahan, A F Sarıkaya, O Agcaoglu, S Danacı, M E Ulutaş, I Hasirci, K Arslan, Ş H Metin, A Yılmaz, E Turan, G Şimşek, A Kılınç, M E Şahin, S Ozden, Y A Bayraktar, A S Maden, A Şahin, N Acar, O Erşen, E Balcı, H Yaldız, C E Guldogan, M M Ozmen, E Gundogdu, M Moran, B Celik, E Sivrikoz, K Karabulut, A C Dural, T Ikizoğlu, H Aydede, T Yalçın, A Dalkıran, H B Yapici, T K Uprak, Y Tatar, Y Aksahin, C Aral, S Z Cetin, D Artvin, Z Tatar, I S Karakuş, S Çatal, B Nalbantoğlu, S Bettar, Z Düzyol, B C Demir, M F Kırcalı, M E Taşcı, F Secil, E N Akkaya, I Kayılıoğlu, B Hekmatjou, A Çağlayan, M A Dadaşoğlu, C Fergar, M S Beden, E Kayhan, I Arslan, G Z Koçyiğit, B Beyoğlu, C Varan, U Sungurtekin, H Sungurtekin, U Özgen, B Çetin, A Pergel, M Uyan, E Erata, E Aldhahebi, B Aslan, Ş Kabalı, F Köse, I Keşaplı, U C Dulger, F Altintoprak, M Akçay, E S Cünük, M B Kamburoğlu, I F Küçük, M Yigit, E Sabuncu, A T Harmantepe, B U Aka, E Baş, T Yavuz Akça, A C Sarı, E Colak, M Candan, M E Kara, A B Ciftci, K Yemez, M S Uyanik, S Polat, G O Kucuk, S Ocak, Y Tosun, E Unal, C B Ofluoğlu, C Üstbaş, Ö Gangal, A N Sanli, S Sayır, C Karslioglu, I Ulusoy, A E Tufan, U Demir, M F Celayir, E Baran, A B Aksoy, D B Fırat, A Yüksel, O Güven, I Ertaş, Z S Kuzu, H M Köksal, M Gok, M N Görgün, P Yazici, S Ömeroğlu, B B Özdemir, E Kabul Gürbulak, M Ajredini, I E Cakcak, I H Atakan, A Göztepe, O Budak, E O Aydoğdu, E Erdem, C Yılmaz, J Özdemir, B Akin, D Alkan, E Cengiz, C N Donmez, E Akyüz, Y H Ergun, M G Ozer, Ç Ak, F Bolat, E Kasapoğlu, A Tatlisu, Y Yorulmaz, A Enez, B Ozkan, Z E Yilmaz, S N Altin, B Gunes, B Kaban, O Korkmaz, I B Demir, G A Öz, A B Tuluce, Z N Turkmen, F Yildiz, B Kandil, A Ulkucu, G Kıral, D Yavuz, Ü C Köksoy, Z C Kus, M B Erten, M Mutlu, F Hökelekli, T Aslan, Ş Orçan Akbuz, E Durmuş, T E Gökçek, H S Ulgur, O F Ozkan, H K Karakullukcu, A Yıldız, M Ceyhan, M Ş Çelik, M Kalın, E F Kirkan, A Demirbilek, M N Çelik, Ö Karbuş, S Demirli Atici, B Abud, H Erdinç, K B Ön, B Sevinç, N Damburacı, E Altiner, B Özdemir, Ö Manisalı, G Ural, M K Topal, T Can, S Ercan, S Kaynar, O Emanet, M Javadov, U B Demir, A Erdem, F Demircan, M Yelmenoğlu, E Seçen, B Alkışer, F Arık, E E Kaya, D Akkad, D Karaçam, U Karacam, M A Yücel, A Hatipoğlu, N Mumcu, O Karima, M B Dal, M Aljobbeh, J Montaser, M A Kara, F A Gultekin, O Deniz, N Kavak, B Kum, M M Ecir, Y G Yavuzer, Z Sezgin, U Koçdemir, B Yirmibeş, Ş Atalay, A May, S Varna, I Aydoğdu, A Abdel-Fattah, G Ramsay, M A Bin Badekruzaman, M Karvelyte, M Siamisang, S Clunie, M Mikalauskaite, A Nessa, C Joshi, N Jodeh, R Zafar, J Miller, R Basharat, T Singh, O Weston, R Loy, Ehc Tsoi, A Chandiramani, R J Sumarlie, L Mitchell, M Yousif, M Qadir, M Gannon, Eyh Tong, J Tsang, M Elmarghani, J Kaczmarek, J Luangboriboon, R Mcewen, B Tse, M C Wong, Z X Wong, T Diffley, O Jaruwattanapradid, N Ng, L Mchaffie, C Wright, D Joyce, V May, S Emmanuel, A D’costa, A Kumar, C Hanganu, S Gourgiotis, A Townson, C Y Williams, E Baines, L Smith, N Elks, N Simon, R Chintapalli, A Banerjee, S Bhogle, B Ryan, D Maghsoudi, E Clifton, T Nott, H Vickers, G D Stewart, Z X Zhong, K Ioannou, Ckh Ne, R Conci, E Fitzsimons-West, X L Ling, R Patel, R Sanghera, I Dokubo, I Seago, O Fairhurst, B Amin, M Aniq, E O’keeffe, S James, M Chowaniec, L Rutigliani, M Hu, A F Ferreira, M Kalogeropoulou, G Hui, L Coakham, S Healy, K Gilanliogullari, G Nishimura, M Choi, R Lunevicius, D Aje, R Mcnicholas, C Newman, E Bollen, A Kumar, I Ferreira Xavier, L G Baloch, N A Koduah-Sarpong, G O’sullivan, S Saseendran, A Bin Sahl, E Murphy, H Hussain, A Nicholls, S O’dolan, A Gidwani, E Patterson, L Loughrey, K Donaghy, K Hana, M Monaghan, S Ni Shandair, N Kulasingha, N Gormley, M Mcglinchey, E Dunlop, B Dunwoody, S H Lian, S C Jayasangaran, E Logue, P Goswami, P Ann Jacob, C O’kane, A S Ab Razak, K Govender, E J Mccann, S Perry, S Mckendry, A Pachchigar, A Singh, S Dindyal, S Pendyala, V Venkateswaran, L Alhoussan, F Feil, T Hughes, Y Gerçek, M Choudry, O Haidar, T Rujeedawa, T Speirs, J Odendaal, J Chu, A Shahab, S Ranjithkumar, V Pillai Rajendra Prasad, T Linn, J Clark, M Sharma, S Lockwood, S Chawla, R Deshpande, N Long, J Rees, M Kobetic, D Fawcett-Till, O Ferris, G Harrison, T Hibbs, Ctw Tsang, L Hurley, T Jichi, C Badrinath, L Applebee, K Ecott, T Sullivan, B Piecha, A Jagadish, Z Sajjad, R Griggs, S Joyce, R Spurgeon, M Ali Khan, M Hobrok, R Roberts, J Mckenna, D Davies, P Eaton, L Bishop, S Magaway, K Denholm, S Doyle, R Deshpande, M Salter, V Chan, K Mockford, J Heinz, I Ahmed, M Coverdale, R J McKillen, M Maguire, N Mclees, J Lau, L Mcguigan, A O’Neill, D O’Hare, C Beggs, O Owolabi, J Hunter, J Kinross, J Kotecha, R Doherty, V Patel, E Wagner, L Hodges, H Hassan, K Sribaskaran, K Pouris, S Jain, A J Kim, S Gill, S Nand, O Toutouza, A Joshi, L Thornley, R Chen, M Ogunjimi, A Mahmood, R Chong, M Yanai, D Bae, J Dhaliwal, A Lovejoy, A R Akhbari, N Mayor, C Kontovounisios, Shk Yap, M Damarla, B Ooi, M A Ng, A Vargas Zhang, S Limbu, K Nyamakope, Y Agarwala, N Zingas, C Cleasby, E Smyth, J Drmota, Qzc Yang, C Vedi, S Samarendra, V Nayak, S Rajendra, M Choudhry, K De Stadler, S Bandyopadhyay, G Bond-Smith, O Collart, K Baffour-Awuah, R Shah, J Mcnamara, G Tadikamalla, B Wilson, C Cossins, H Li, L Ismail, H Soleymani Majd, E Chang, A Jallow, S Baldelli, P Alberti, O Grant, M Doody, Z Borawska, A Bowman, H Clay, Y Petit, J G Kimani, M James, S F Hussain, A Nezhentsev, M Emmerson, R Vijjhalwar, G Shaw, C Holmes, Y Ying, L Farache Trajano, A Anis, J Dequaire, A Hunter, R Danvers, M El-Nemr, C Hammett, M Pikoula, S Thompson, B Lander, S Dierksmeier, N Sadeghi, R Suribhatla, R Ahmed, S Pandit, W Thornton, T Thornton-Swan, A Johal, M Khan, A More, Y Tilahun, A Hauperich, R Gidda, I Vorley, Z Khan, M Sintler, A Van Den Broeck, M Georgiou, N Eardley, H Salem, S Yemparala, L Whittle, C Jagger, K Turner, L Vernon, K Aldred, P Manokar, A Moore, N Mistry, J Zgliczyńska, C Haylett, A Adeyanju, E Headford, R Khanna, M Deef, T Durkin, E Trayling, R Cowell, Z Khan, A Turner, K Noureldin, W Down, A Cyril, C O’halloran, M Shirke, M Epanomeritakis, A Karnati, G Sreejith, C Sinton, L Oliver, H F Ali Azamatullah Khan, E O’kane, J Trouton, B French, I Campbell, R Curran, C Brines, A Mcdermott, N Yang, S Vig, S Chowdhury, R Valecha, R Lau, M Nafis, S Thavanesan, J Marks, L Akaje-Macauley, N Patel, E Beard, A Nanda, C Mills, A Cheung, J Amalendran, L Todd, S Smolarek, T Warrener, S Sen, A Mudehwe, S Khan, B Wisden, G Lau, L Schanzer, Z King, K Giridhar, M Reed-Embleton, L White, P Filippidis, L Lee-Smith, O Carless, C King, M Herriott, N Saju, Y M Tin Maung, A Deligianni, A Mumtaz, S Khan, H Hill, S Sahdev, I Thomas, A Shah, S Saji, J Hargreaves, R Khan, A Tharani, R Ullah, J Ludgate, S Shrestha, E Bota, A Abdullah, M Allison, Z Patel, A Clark, I Suchett-Kaye, B Stubbs, B Holmes, A Santaniello, T Watkinson, A Crimmins, M K Gupta, S Abraham, A Bellringer, R Clowes, D Chatzopoulou, A Kiran, J Eid, N Rao, J Caterson, F Soggiu, H Malik, I Jose, K Kavallieros, C Rizk, B Thummala, K Li, A Goel, R Pantula, J Toh, M X Fu, C C Ho, K Vivek, E Owen, A Day, A Jamieson, H Hassani, T Thavarajah, M Vivekanandan, A Muneer, M Chauhan, D Veeramootoo, S Ali, J Dosanjh, F Newing, E Jose, H Chauhan, A Kulshreshtha, E Thomas, P Patel, R Chhabra, B Sajan, S Ragavan, R Hariharan, I Lam, B Fleet, N O’hara, A Wright, V Reddy, H Darweesh, A Khan, S Handa, D Kewada, F Rana, H Williams, F Bombieri, N Shah, D Pestotnik Stres, A Menon, L Selvam, A Pusok, C Street, M Zohdy, B Aguirrezabala Armbruster, I Okoye, L Potts, M Fayyad, K Bajaj, H Alfa, N Sivakumar, N Duncan, C Roxburgh, L Huang, K Lukito, X Huang, S Baig, C Y Chan, R Philip, L Shaheen, J Dodds, H X Yeow, M Devindran, Z C Sia, J H Park, B Furze, N Yung, M Vipond, M Jones, F Asekun, A Dembinska-Kenner, R Saleh, E Larkai, J Harris, M Cherian, H Louden, V Bisbinas, A Behl, R Hughes, R Smith, Z Zaman, O Hoskyns, H Carlton, C Thorn, M Tourky, J Tan, K Sen, J Elsey, J Bevan, N Ko, S Kalidindi, M Rooney, K El-Boghdadly, S Zafar, A Wyncoll, P Morillon, M Sennaraj, H Mahfouz, A Afzal, R Sibal, T Suji, H Headon, A Abdul, S Ahmed, J P Mcnally-Reilly, K Omran, C Mulcahy, M Aftab, M Haghighat Ghahfarokhi, M Farhangi, W Y To, M Ho, T Patel, A Siu, H Choudhry, M Huntley, Z Hussain, G Wong, R Maguire, M Khan, A Potts, M Ahmed, G Ramesh, O Kolade, M H Siddique, E Griffiths, M Qureshi, M Hoque, S Laulloo, K Das, N Waheed, Tyt Tang, R Ahmed, R Habib, R Vyas, S Watson, K Theodoropoulou, M Siu, N Yu, N Islam, J Burnett, A Besso-Cowan, D Hirani, I Zsolnai, Y K Chen, O Dupere, L Keitley, C Park, I Verma, J Chan, J Tavner, M Nicolaou, Nys Lee, R Hegy, Rby Lee, Kyl Yi Lun, D Reyes, S Dong, J Thornton, P Eaton, L Bishop, S Magaway, K Denholm, S Doyle, R Deshpande, M Salter, M Weir, A Gibbs, A Al-Shaye, M Alwahid, A Tait, S Smith, A Doye, K L Law, T G Groot-Wassink, G Geller, K Seebah, O Cox, M Kalogeropoulou, A Shetty, Dcj Oh, E Lee, B Packham, M Aarons, K Saadeh, J Mui, R Huynh, M Eid, N Honey, J Kaur, R Hand, L Lai, C Koubaesh, H Maqsood-Shah, J Suresh, O Collart, G Bond-Smith, R Shah, J Mcnamara, G Tadikamalla, B Wilson, C Cossins, H Li, H Soleymani Majd, L John, E Chang, J G Kimani, A Jallow, S Bandyopadhyay, S Baldelli, O Grant, M Doody, Z Borawska, A Bowman, C Foster, H Clay, Y Petit, M James, S F Hussain, A Nezhentsev, M Emmerson, R Vijjhalwar, G Shaw, C Holmes, Y Ying, L Farache Trajano, A Anis, J Dequaire, A Hunter, R Danvers, M El-Nemr, C Hammett, M Pikoula, S Thompson, R Shah, S Dierksmeier, N Sadeghi, B Lander, R Suribhatla, R Ahmed, S Pandit, W Thornton, T Thornton-Swan, A Johal, M Khan, A More, Y Tilahun, A Hauperich, R Gidda, I Vorley, E Lewis-Orr, S Reilly, A Bhatia, K Goves, M Blesson, L Purser, L Cobb, M H Sarker, N De Sousa, S Syed, N Rajendran, J Tan Sue Wei, G Navakumar, V Butnari, M Senthilkumar, Z Q Chew, L Zekaite, S Paranietharan, M Haque, S Balenthiran, K J Chin, S Ahuja, T Moothathamby, F Moniati, Y Z Kong, Ejh Lee, L Sharma, P Found, Y Sivakumaar, H Senior, N Simeen, A Arora, A Chu, R Mizori, T Beazer, N Patel, J M Dudziak, T Squeri, F Weston, M Zhou, J Van Ross, S Tian, J Bedford, R Lam, F Karami Tireh Shabankareh, H Donkin Everton, K Wilson, A Richens, D Bragg, A Akbari, A Awodiya, S A Osula, F Gerges, I Gerogiannis, L Daniels, S Seth, Z Baxter, E Nour, L Fitchford, C Perrott, M Abuelgasim, J Flanagan, S Gillani, H Lewis, O Dunscombe, P Steven Goodwin Moughton, A Mahmood, J Hirniak, A Moses, P Kapsampelis, A Ahmed, D Burke, C Anderton, E Lee, M Conley, L Khan, R Surti, F Waseem, M Sharma, A Imran, R Motiwale, B Singh, S D Sa, S Charuvila, M Z Lorgat, R Chowdhury, P Gogineni, A Finney, A Tzortzi De Paz, S Panesar, A Sidki, C Warwick, S Kadambande, J Patel, A Rao, A Sharma, S Ramewal, P Ramesh, M Norwood, P Mehta, M Bhatia, S Tiwana, E Foote, F Rushton, S Asi, R Notaney, R Sinha, J Vibhishanan, S Gupta, R Conci, R Chintapalli, S Ahmed, J Sagar, N Ragge, S S Rizvi, M Ashraf, N Cirocchi, R West, E Obiri-Darko, Y Rai, S Hussain, N Ul Ain, F N Amir, C Smart, R Melomud, D Saad, S Bengeri, S Kuna, M Chaudhary, O G Olanite, F Khan, S Nausheen, R Dodds, H Boyden, D Donnelly, O Adegboye, L Osborne, S El-Barraj, J Shukla, N Raza, A Khalid, D Agrawal, K T Kyaw, S Borhan, G Pangrazi, I Ioannou, A N Ahmed, G Savvides, M White, S J Puthur, S Bridgewater, N Waraich, R Bryce, R Khosla, L Penhaligan, A Desai, Z Ehsan, O Mostafa, Y Kamel, B Keeler, R Rajivan, V Omar, S Gamadia, D Rana, S Srikumar, A Heidari, A Ahsan Akhtar, A Tarafdar, J Barry, D Davies, A Curr, I Jimenez-Reid, J Mckenna, E Garry, N Lallmahomed, K O’leary, E Barker, D R Jones, R Tabatabai, J Garg, E Henshaw, M Abdulshafea, S Prabaharan, H Wiles, R Bamford, F Blest, L Acquah, H Simpson, J Guerrero Enriquez, H Pringle, M Shahid, O Wharf, M Kumar, K Buadooh, M Hanson, I Mutanga, A Hardy, H Usman, S Shams, N Schottler, A Garg, V Sarodaya, S Ali, I Cullen, L Pregil, M Fernandes Silva Ramos, Y Hao, A C Das Chagas E Silva, S Sellahewa, J Li, J Cox, P Sinha, Ijj Lee, E Voniati, H Phillips, O Mooney, M Sockett, P Patil, M Elsllabi, Wkm Chan, L Grimble, H Richardson, A Johnston, T Kouli, Jfm De Sousa, Y K Goh, C Grant, L Martin, Z H Peh, T Yap, Etw Tang, Hym Lai, T Berry, M Rokia, E Tennant, I Lee, M J Rahman, A Kamal, S Ali, H X Hau, I Harten, M Eloofy, C Caldwell, R Leckie, F Tasnim, K Ravintharan, J Mcauley, F Cameron, R B Veerni, K Y Looi, A Ismaili, R Gresz, V Chong, L K Tan, K Singh, S Ashburn, R Shantha Kumar, M Kamal, H Kamal, I Aziz, P Stather, L Mylvaganam, E Deliyannis, B Tompkins, E Sikorski, F Harris, I Sanders, M Fakhrul-Aldeen, K Cross, A Tahir, P Yim, I Mayne, S Mahapatra, C E Ng, A Ortega, S Supparamaniam, M Bowman, M Sadler, M Rogger, A Gendia, J Ahmed, P Sivakumaran, C Brick, E Ansong, H Ha, A Sharma, N Wilson, B Manavi, L Zeze, N P Gupta, S Al-Hassani, C Williams, M Ramesh, B Shukla, E Drye, H Hussain, E Ghatauray, Acw Tan, F Adamu-Biu, J Arora, T Mcallister, S Fairclough, A Economou, S Utulu, K W Fung, I E Epanomeritakis, S S Zaman, E Olszewski, P Ballesteros, E C Okpii, E Walkeden, C Macklin, S Jamil, S Zhen, O Ahmed, A Saravana Kumar, H Uddin, R Hakim, M Mossanen Parsi, O Awoloto, K Rowe, R Mashadi, Z Mohammed, L C Chong, S Fairlie-Vogt, O Russell, W Ozarek, A Little, C O’farrell, R Miller, J Froud, K Thippeswamy, R Thomas, A Fitzpatrick, S Yoganathan, M Dunlop, K Ngai, T Liddell-Lowe, A Barrow, L Sharma, P Found, R Mizori, S Rabas, R Al-Housni, M Zhou, A Marton, P Chen, H Hasan, Cfb Chan, W W Win Mar, A Kisiel, E Griffiths, M Yusuf, A Sinha, J Blenkinsop, K Rawlings, T Chaudri, G Westland, H Umar, S Lee, J Cherian, Z Gurhan, T Chaudri, M S Sheraz, Dmi Khan, A Kucukmetin, D Malone, C Betts, M Ali, M Giblin, L Nowicki, P Korompelis, O Farley, M Fouweather, C Ioannides, H Yilmaz, R Landais, E X Ngeyu, C Erinjeri, D Badran, P Glen, J Aamir, A Aziz, P L Su, I Choong, M Reece, A Kulasekaran, Tns Tengku Saifudin, J Luckhurst, R O’hare, R Smith, Y T Siu, P H Kwok, V Sood, A Imran, K Fatima, Z Munir, T Kisova, N Rajendran, F Islam, E Teehan, H Sadik, U Patterson, N Rahman, H Harrisrhaj, D Govardhan, S Rajesh, Z Mumtaz, O S Ghori, H Nawaz, A Lysomirski, V Pikoula, Rmab Qadir, A Jones, Y Chiang, H Morsy, C Pazaiti, A N Asardag, I Ali, J Althonayan, A Badri, J Evans, E Chung, L Harriss, L Sinan, C Maxwell-Armstrong, A De, R Altman, F Varghese, W Chua, S Ahmed, M Javed, R Aljubure, A Bradley, S Moug, Oeh Kemmett, I Underwood, I Fitzpatrick, L S Wong, B Vakeesan, K Potter, T Varghese, S Daren, A Hazrin Fazail, S Farajzadeh Asl, H Nautiyal, A Saadeh, I Vial, R Karimi, A Akthar, A Egiz, R Masood, A Qadri, T Chawla, H Yoon, X Liu, N Battersby, E Davies, R Chhetri, R Mclean, E Leaper, C Taylor, C Nicolaisen, L Cochetti, E A Zoumi, D Annable, H Thomas, Z James-Knights, C Lee-Kim-Koon, E Akpinar, M J Coelho, E Darke, T Morris, F Mayer, J Forbes, S Gold, H Sinha, E Batchelor, L White, J Lund, S Forrest, H Dial, F Chishty, M Oliver, J Meyer, N Gokhare Viswanath, A Ammar, A Lopez, N Minhas, N Sunny, K Haynes, A Alexiadis, S Doski, R Arbuthnott, R Memon, K Bukhashem, A Chaudhary, A Sheik-Ali, A Sebastiao, C Taylor, E Clarke, S Misztal, D Raja, S K Chui, I Khalil, A Metsel, M Whelan, S Forrester, O Oboh, B Bayley-Skinner, R Eastwood, E Akapo, M Jamshaid, G Osborn, R Woods, O Wooler, S Chumley, E Cotton, N Jarrett, N Amanda, J Johnson, M Quhill, M Al-Ani, S Kandanearachchi, S Khan, A Barrow, M Dunlop, G Williams, S Phillips, F Faryad, A Hughes, S Wong Ching Hwai, C Johnston, R Guest, A Kaddouri, L Ernst, A Szasz, C Gafrey, G Loy, E Roberts, S Baker, A Davies, Z Khan, M Leonidou, J K Chow, R Murugan, A Baker, R Burns, Y C Foo, F Sikora, L Chong, C Chan Ah Song, C Cosgrove, O Reeve-Chen, J Ma, L Mccolm, M K Zhang, F Hussain, X Y Ng, B Ingabire, B Wagner, T Bain, R Kovacs, E Small, S Lam, L Yao, L Ho, H Paremes Sivam, K Dahal, A Stanley, M White, L Hayois, M Thillai, T Tay, E Davies, N Kamaruzaman, L Robinson, R Sherrington, Z Abid Sohail, S Lawrence, A Sohail, D Brown, S Parker, M Ohr, D Jasniak, B Crowther, F Jadoon, L J Melo, H Amin, Rqh Lim, M A Thaha, M Bath, S A Saadqain, Y Benallal, Z Jin, A Jackson, A Kuri, M Malik, H Jos, E T Goh, M E Paraskevopoulou, A Kythreotis, Y Naim Ahmed, S P Glynou, H Rehan, A Pereira Pai, A Georgiannakis, N Dworschak, S Cho, B J Chow, Y Khan, T Enthoven, A Malik, I Latif, A H Lakhani, N Rahman, K Morgan, S Gkolia, N Khalessi, Plz May, A Lwin, P Kanesaratnam, M Tudgee, R English, K Hu, N Pathanchaly, Dcs Chien, M Holloway, K Gao, G Lekka, R Srinivasan, S Zafar, M R Peris, K Keiarash, H Kanesan, B Chong, M Kobus, D Dominic, D A Do, D Dennis, S Frankland, V Shah, B Youssef, U Sadia, A Hassouneh, A Jaipersad, T Chen, M Fulford, A Dawson, K Kapur, T Jha, P Zope, M Wilkinson, V Bhatnagar, J Knight, T K Madhuri, T Katsiari, N Pasternak-Albert, E Moussa, L Emms, M Lamah, Z Batool, A Sabesan, S Islam, M Baig, M Dave, X Liu, L Stuart, S Harvey, V Dam, P Ezuma, J Lee, S Suntharalingam, S Singh, P Preston, F Rickard, T Gupta, S Madden, C Jones, R Gidwani, X Wang, M Mcconnell, S Hanna, A Mcgettigan, K Brown, A Cios, C Ward-Bradley, O Hurrell, V Paice, N Mutsonziwa, S Pogoson, R Coulter, J Corry, L Mcclean, F Keenan, S O’dolan, A Plonkowski, R Brady, R Ooi, D Iles, L S Guillemot, C Low, S El-Barraj, J Epstein, M Pressler, I Ioannou, N Soliman, A Ktayen, A Macconnachie, J Menendez Lorenzo, J Tooke, K Noman, L Wilkins, J J Warner-Levy, R Evans, A Torrance, C Fear, H Vidis Humphries, G Velayudham, Z Jefferson-Pillai, J Brady, R Oza, D Singh, A Hamid, P Hartop, P Shojaie, J Mano, T Rajah, Ymc Cabdi, A Singal, M Afzali, J M Lee, D Bandyopadhyay, J Bennett, P Mendenhall, A Millett, S Davidson, R Kallam, M Pishia, H Butler, S Isidore, D Boutsias, K Bryce, T De Rancourt, J Barry, H Henry, A Aspinall, L Smith, C Conway, H Shanmugathas, C Huang, M Doherty, K Ahmed, T Chung, T Benson, A Habiba, J Singh Bhangu, B Bingor, L Fice, H Premanandhan, J Mckenna, I Jimenez-Reid, M Hoque, E Griffiths, J Loughran, M Laskowski, A Mehmood, R O’kane, M Mullan, R Mcclenaghan, G Kettyle, F Hosty-Blaney, Xhf Chan, S Sunny, M Kaur, S Vose, V George, D Killoran, R Doherty, Z Zagorac, A Pullyblank, R Ismail, N Anderton, L Dwyer-Joyce, R Morgan, N Zhang, B French, H Cox, J Tetro, A Clarke, K Shaw, R Iau, E King, S Patel, M Ovakimian, A Zahid, G Rowley, R Tanner, K Davoudi, B Shear, M Xu, K Karan, A O’reilly, S Ahmed, T Iskrenova Kirilova, A Ezekiel, M Shapland, M Mitra, T Krum, G Higginbotham, R Winayak, J Mustow, L Yorke, C Gibb, A Ilyas, M Pound, A Abouharb, D Burke, A Khan, K Aimar, J Bennett, E Kerman-Fiore, S Rao, A Dayal, S Chawla, L Wadey, C Smith, K Desor, R Surti, Z Gul, V Gourgiotis, L M Kaselampao, C Nguik, R Agrawal, Q Y Liaw, E Mckeown, A Hassan, K Krishnan, H O Glover, M Devassy, D Ogunyanwo, G Dickenson, G Elder, G Valdez, B White, I Hamilton, S Shah, S Sinha, K Punwani, I Peat, M Hayat, N Khalid, E Frankel, H Macgowan, A Braka, A Byfield, J Brooks, A Kashif, R Archer, S Akkaya, M Sood, S Akther, O Birkett, T Kadri, S Abdulmula, P Psefteli, S Chadha, G Reese, C Mark, M Asunramu, T Hess, S Mehta, C Tsang, K Syed, A Alocious, M Steinruecke, M Ashley, R Newton, J Wainwright, J Ayathamattam, H Wong, Z Elahi, M Fleming, T Ali, D Lloyd, K Brooks, C Kwon, A Mckerrell, T Jamadar, M D Barcelona, D Polluk, J Kwon, F Henry, S Ayirookuzhi, M Mwipatayi, M Cheruiyot, S Adam, T Cruickshank, K Gollub, M Revell, S Taiwo, A Atchade, A Harewood, A Niina, D Bosanquet, O Mckeon-Williams, T Szakmany, E Badhams, N Christensen, J Sammut, N Tay, J M Pollok, D Raptis, M Varcada, S Ganesananthan, R Chandrasekar, A Deshpande, L P Ghoora, G Hogg, S Staubli, M Banerjee, S Sunil Menon, A Sanz Pena, R Rana, A Mohamed, P Crabtree, A Flower, A Banerjee, Y Talabi, N Bishop, N Kupfuwa, D Ilangovan, H Qureshi, I Minty, R Baron, L Greasley, P Birkenhead, R Baron, M Shahid, N Rajasivam, W L Chu, N Punnoose, V Sundaresan, M El-Galley, K Prasansapakit, S Mitra, M Garlick, M Prakash, H Gao, C Parmar, F Lee, A Aich, A Ismail, M Kirupaharan, S Khan, K Patel, D Mclaverty, G Hammerton, H Walford, H Roberts, P Cautivo, H Abdel Kader, A Kimble, K Manaf, R Mackonochie, V Mitchell, S Ghaznavi, A Taheem, E Firth, H Chandler, K Eldessouki, J Fyfe, M Bhat, J Cavlin, E Pearce, S Patterson, C Johnson, K El-Badawi, M Gariballa, E Hale, H Younis, E Brackenbury, A Chappell, T Poulton, E Dawson, A Murray, I Mcallister, A Hassan, B Cunningham, T Chan-A-Sue, N Rajpal, P Keane, K Walsh, L Mcgeoghan, M Neeson, H Brown, S M Adams, S Ladha, A Walsh, M Alradhawi, R Tarighi, M I Miah, K Dawas, A Mojadady, A Priestman-Degano, S K Vellore Sasikumar, B Suresh, M Fornasiero, D Csvila, A Kumar, S T Adil, H S Adil, U Kataria, R Jaibaji, N Patel, A Goch, P Quaye, A Gupte, H Mustafa, V Otti, D Dewantoro, M Ali, M Sood, H Wright, L Abusheba, L Wong, M Docksey, A Abdinasir, M O Karim, V Kolaityte, M Vasileiadou Pelling, A Abdel Basit, E Pearson, E Hughes, A S Millington, L Taylor, C Borg, K Jagic, A Waheed, K Singh, V James, T Chowdhury, A Saxena, A Georgiou, T Jones, C Carpenter, I Hughes, Z Aloul, C Hanna-Davies, A Jacob, A Puthiyakunnel Saji, A Qureshi, Z Ulfin, M Dunlop, R Woods, N Hill, N Mohamed, A Vora, H Asharaf, E Maye, U Arif, S Srinivasan, S Prasad, B John, V Shivanand, A Singh, A Zahid, E Stewart, M Raketla, O Kokoricha, D Karwa, J Crisp, R Shehadeh, J Lee, B Webb, S Lepping, F Yusuf, E Pak, B Soo, P Pemmasani, S Goyal, A B Binti Azad Bashir, S Komolafe, A Naeem, H R Anbananthan, L K Au Yong, S Mookerjee, N Ward, M Aniq, J Davis, A Al-Sukaini, J Anthony, J Boyle, A Laird, D Speake, C Beagan, S Stewart, O El-Koubani, R Chan, M Viswanath, S Janssens, I Shah, V Nguyen, E Mckee, C W Ng, M Balać, D I Suresh Kumar, S Seeva Balan, R Kirk, B Miles, L A Kovacic, V G Collins, J Low, W Sim, S J Chua, N Narayanan, T Y Lee, Cmn Lo, M Chauhan, J Elkafsi, S Rasul, R Yammine, Z Sattar, D Dixit, N Rahman, J Davies, S Dindyal, L Osborne, L Nip, N Wilding de Miranda, A Stevens, B Al-Diri, A Lim, G Kallikas, Y Kim, M Anjum, P Jeyapahan, M Hakim, S Singh, E Jamileh, C Sohrabi, S H Baek, N Sadik, A Mohammed, D Aje, R Clifford, C O’halloran, K Mahajan, N Darke, S Lloyd, M Mlotshwa, K Melhuish, J Derex-Briggs, M Bell, P Varma, K Fray, Y Garg, K Datta, T Mantel-Cooper, M Goodfellow, B Kazi, N Sievers, K Telford, Z R Almansoor, K Craddock, J L Tan, L St John, A Singhania, S Dosani, S Mughal, N Bokhari, L Brooks, I Laid, A Lala, C S Ong, S Wakefield, S Phillips, H Unwin.

## Supplementary Material

znaf005_Supplementary_Data

## Data Availability

The data that support the findings of this study are available upon request from the corresponding author. The data are not publicly available due to privacy or ethical restrictions.
